# Surface Layer Alteration of Multi-Oxide Silicate Glasses at a Near-Neutral pH in the Presence of Citric and Tartaric Acid

**DOI:** 10.1021/acs.langmuir.1c02378

**Published:** 2022-01-13

**Authors:** Juho Yliniemi

**Affiliations:** Fibre and Particle Engineering Research Unit, Faculty of Technology, University of Oulu, Pentti Kaiteran katu 1, Oulu 90014, Finland

## Abstract

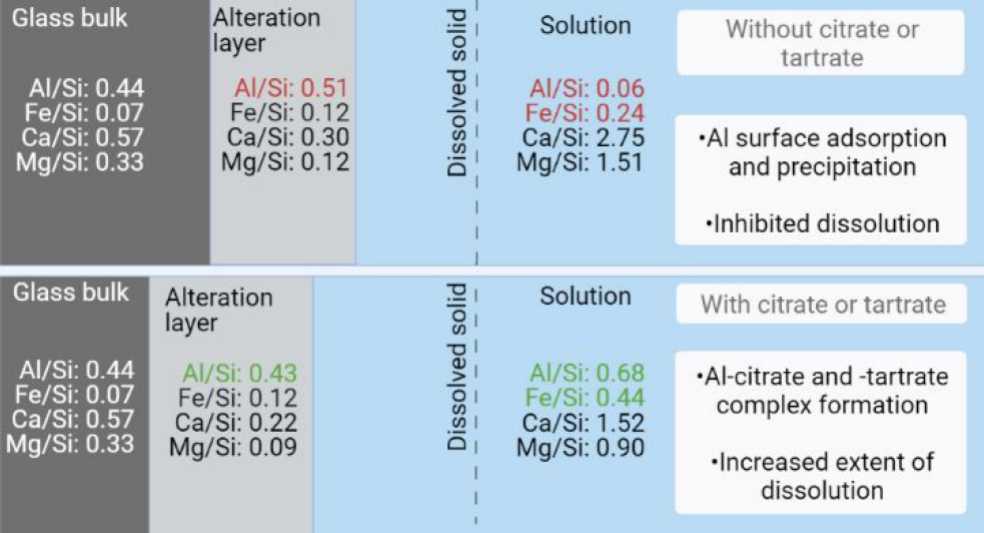

This study aimed at determining the chemical alterations occurring at the surface of multi-oxide silicate glasses in the presence of organic ligands—citrate and tartrate—at a near-neutral pH. Batch surface titration experiments for basaltic glass and blast furnace slag (BFS) were conducted in the range of 6.4 < pH < 8 to investigate the element release, and speciation and solid phase saturation were modeled with PHREEQC software. Surface sensitive XPS and zeta potential measurements were used to characterize the alterations occurring on the surface. The results show that, while Al/Si and Fe/Si surface molar ratios of the raw materials increase at a near-neutral pH, the presence of organic ligands prevents the accumulation of Al and Fe on the surface and increases their concentration in the solution, particularly at pH 6.4. The Al- and Fe-complexing ligands decrease the effective concentration of these cations in the solution, consequently decreasing the surface cation/Si ratio, which destabilizes the silicate surface and increases the extent of dissolution by 300% within the 2 h experiment. Based on the thermodynamic modeling, 1:1 metal-to-ligand complexes are the most prevalent aqueous species under these experimental conditions. Moreover, changes in Ca/Si and Mg/Si surface ratios are observed in the presence of organic ligands; the direction of the change depends on the type of ligand and pH. The coordination of Al and Fe on the surface is different depending on the ligand and pH. This study provides a detailed description of the compositional changes occurring between the surface of multi-oxide silicate materials and the solution in the presence of citrate and tartrate. The surface layer composition is crucial not only for understanding and controlling the dissolution of these materials but also for determining the activated surface complexes and secondary minerals that they evolve into.

## 1 Introduction

Various industrial materials, such as glasses, cements, and ceramics, consist of multi-oxide silicates. In contact with the solution, the solid silicate surface can undergo a number of different reactions, such as dissolution, leaching, adsorption, and precipitation. For research fields such as nuclear waste stabilization in glasses,^1^ geosciences,^2^ reactivity of cements and supplementary cementitious materials,^3^ and biosolubility of glasses,^4^ to mention just a few, it is central to understand the mechanisms involved. In particular, it is crucial to understand the alterations occurring on the solid surface.

When in contact with water, a leaching of cations from the silicate surface occurs by proton–metal exchange reactions, the depth of which depends on the solid and solution properties and the experimental conditions.^2,5−7^ At 2 < pH < 5, all cations are typically leached, while Si dissolves only at a very low rate. At 5 < pH < 9, mostly mono- and divalent cations (e.g., Na^+^, Ca^2+^, Mg^2+^) are leached, while trivalent cations, such as Al^3+^, are not detected in the solution.^5,6^ At pH > 9, lesser amounts of divalent cations are leached, but the dissolution of Si tetrahedra is promoted by the nucleophilic attack of OH^–^ ions, particularly at pH > 12. As Si is the main network former component, the dissolution rate of silicate glasses mainly depends on the polymerization degree (connectivity) of the SiO_4_ tetrahedral units and Si content,^8^ although factors such as pH, solid surface area to solution volume ratio, accumulation of Si in solution, and precipitation of secondary phase have an effect as well. The release of Si can be taken as an estimation of the extent of the dissolution of the raw material in cases when re-precipitation of Si can be excluded.

The leaching of cations will create a new metal-depleted, Si-rich surface layer, which is often referred to as an “alteration layer”, “leached layer”, or “gel”. The surface of this layer can have a negative, neutral, or positive surface charge depending on the solution pH and if specific adsorption of elements on the surface occurs.^5,6^ The solid surface is most stable when the surface charge is close to neutral and, correspondingly, the most unstable when the charge is highly negative or positive. For simple compounds, such as SiO_2_, Al_2_O_3_, and Ca(OH)_2_, the pH at which the surface charge is neutral is also the pH at which the dissolution rate is the lowest.^6,9^ Typically, the Si-rich materials have a negative charge at acidic and near-neutral pH due to the depletion of positive cations from the non-bridging oxygen sites and deprotonation of the surface >Si–OH (silanol) groups, where “>” represents the surface. However, due to the negative surface charge, the cations present in the solution may adsorb to the Si-rich surface, which consequently neutralizes the surface charge, stabilizes the Si tetrahedra network, and decreases the rate and extent of dissolution.^7,10^

Basaltic glass is one of the multi-oxide silicate materials that is of interest in various fields of science.^5,11−13^ Basaltic glass dissolution is governed by the Al releasing exchange reactions between one Al^3+^ and three protons at 3 < pH < 11.^11^ The dissolution rate mimics the solubility of the Al-hydroxide mineral (gibbsite): the minimum rate is around 6 < pH < 8, while the rate sharply increases under acidic conditions (pH < 4.5).^13^ Under alkaline conditions (pH > 9), the Al is released by OH^–^ ions, which promotes the dissolution mechanism and the formation of the aqueous Al(OH)_4_^–^ species.

The low dissolution rate of basaltic glass at a near-neutral pH is related to adsorption of Al^3+^ on the negative silicate surface and the precipitation of Al-(hydr)oxides.^5,14^ In a study by Okhrimenko et al., nearly all of the potentially leached Al was adsorbed on the surface of the basaltic glass and formed stable surface complexes,^5^ while Houston et al.^14^ also found that Al-hydroxides and aluminosilicates may precipitate, explaining the missing Al in the leachate and the low solubility of basalt glass at 6 < pH < 8. The organic ligands that can form soluble complexes with Al, such as citrate and tartrate, have been observed to increase the Al-containing silicate glass dissolution rate, particularly under acidic conditions (pH < 5.5).^11,12,15^

However, while increased dissolution rates in the presence of organic ligands have been observed,^11,12^ the experiments have relied mostly on the analysis of the elements release into the solution; moreover, data about the effect of organic ligands on surface alteration layer composition and solution speciation^16^ are limited. Furthermore, even though citrate and tartrate have been reported to increase the stone wool (∼basaltic glass) dissolution rate, no experimental data for tartaric acid have been provided.^12^ The evolution of the Al/Si, Fe/Si, Ca/Si, and Mg/Si ratios on the alteration layer of multi-oxide glass is important to determine the dissolution progress, the surface adsorption of elements, and the surface-enhanced precipitation of the solid phases. For example, with a certain Al/Si surface ratio, the precipitation of zeolites may occur, which will be accompanied by the resumption of the long-term dissolution of glass.^17^ How these ratios vary as the effective concentration (chemical activity) of aqueous cation changes due to the complex formation with organic ligands is therefore of importance.

To generalize, dissolution reactions can be considered to be based on the attack of H^+^/H_2_O/OH^–^ to the reactive surface groups under acidic/neutral/alkaline pH conditions. The attack leads to the formation of surface complexes that mediate the detachment of cations. The overall dissolution rate will slow down or stop completely once the surface layer attains equilibrium with the solution, i.e., when the chemical affinity for the surface layer dissolution reaction approaches zero;^18^ at near-neutral pH, particular importance is related surface-adsorbed Al.^19^ However, also other anions, such as organic ligands, could form surface complexes. The hypothesis is that ligands could participate in the dissolution reaction in two ways: forming a surface complex that can enhance or inhibit cation release and/or forming aqueous complexes that contribute to increase the total concentrations of metals in solution by reducing the cation activity (e.g., Al^3+^, Fe^2+/3+^, Ca^2+^, Mg^2+^), consequently lowering the ion activity product (IAP) of the precipitating phases and the adsorption of ions on the silicate surface. By changing the effective concentration of cations in the solution through the aqueous complex formation with organic ligands, the dissolution process will continue.

This study will focus on the surface chemistry and surface compositional changes of two multi-oxide silicate materials, namely, basaltic glass and ground granulated blast furnace slag—at a near-neutral pH (6–8) and in the presence of citrate and tartrate. The purpose is to report the experimental results and to use them to further illuminate the effect of organic ligands on the composition of the alteration layer and the solution.

## 2 Experimental Section

Two amorphous CaO–MgO–Al_2_O_3_–FeO–SiO_2_ materials were used in this study: basaltic glass and ground granulated blast furnace slag. The basaltic glass (**BG**, Na_0.13_K_0.02_Ti_0.03_Mg_0.33_Fe^II^_0.07_Ca_0.57_Al_0.44_SiO_3.85_) was made of stone wool fibers produced without organic resin or oils, which are typically added on stone wool products. The fibers were crushed by loading the material into a steel cylinder (4 cm in diameter) and applying a 20 ton force for 1 min with a hydraulic press. The crushed material was collected and sieved to <45 μm. The blast furnace slag sample (**BFS**, Na_0.03_K_0.02_Ti_0.03_Mg_0.44_Fe^III^_0.02_Ca_1.23_Al_0.32_SiO_4.47_) was from Finnsementti, Finland, with the product name “KJ400”. The iron in basalt glass is mostly Fe^2+^ due to the reducing conditions during the manufacturing process.^20^ Based on the literature,^21,22^ iron in BFS is expected to be mainly metallic Fe^0^ but is reported here as Fe^3+^ (i.e., Fe_2_O_3_) for easier comparison with the materials used in previous studies.

The chemical compositions as oxides of raw materials and their physical properties are presented in Table S1. Based on the XRD analysis (Supporting Information, Figure S1), both raw materials are amorphous, with only trace amounts of crystalline or nanocrystalline minerals.

### 2.1 Titration Experiments

The suspensions of BG and BFS were titrated using the Mettler Toledo T5 with a sealed cell equipped with a pH electrode, which was stirred constantly with a propeller and purged with N_2_ gas to prevent contact with atmospheric CO_2_ and to remove O_2_ gas from the water. The experiments were carried out at room temperature (23 °C). Details of the titration are presented in the Supporting Information. The initial solid-to-water ratio of the suspension was 20 (i.e., 50 g of ultradeionized Milli-Q water and 2.5 g of raw material). The solid surface area to solution volume ratio was approximately 17,500 and 27,500 m^–1^ for BG and BFS, respectively. The pH was monitored throughout the experiments. The pH stabilized in 5 min to 10.0 and 11.2 for BG and BFS, respectively, and these values were considered as the immersion pH (pH_i) for these materials. No background electrolyte was used in the titrations to avoid complicating the experiments and to avoid possible interference with the ligands.

Each suspension was titrated with an acid, as shown in Table 1. The HCl solution was a stock solution by FF Chemicals, with 0.5 M concentration. The citric and tartaric acid solutions were prepared from reagent grade chemicals (citric acid by TCI and d-(−)-tartaric acid by Merck) to form solutions with a concentration of 0.16 and 0.25 M, respectively. The ionic strength at the end of the titrations varied from a minimum of 0.04 mmol/L (BG_i) to a maximum of 227.4 mmol/L (BFS_CA_6). Replicate titrations were done for the selected samples, and the variation in acid consumption was on the order of ±0.01 mL.

**Table 1:** Sample Design and Acid Consumption during the Titration Experiments

sample code	acid	target pH	acid consumption (mL)	added protons (mol)
BG_i		pH of immersion (10.0)		
BG_8	0.5 M HCl	8	0.091	4.55 × 10^–5^
BG_CA_8	0.16 M citric acid	8	0.122	5.86 × 10^–5^
BG_TA_8	0.25 M tartaric acid	8	0.086	4.28 × 10^–5^
BG_6	0.5 M HCl	6.4	0.163	8.15 × 10^–5^
BG_CA_6	0.16 M citric acid	6.4	0.441	2.11 × 10^–4^
BG_TA_6	0.25 M tartaric acid	6.4	0.238	1.19 × 10^–4^
				
BFS_i		pH of immersion (11.2)		
BFS_8	0.5 M HCl	8	5.830	2.92 × 10^–3^
BFS_CA_8	0.16 M citric acid	8	4.760	2.28 × 10^–3^
BFS_TA_8	0.25 M tartaric acid	8	5.069	2.53 × 10^–3^
BFS_6	0.5 M HCl	6.4	19.117	9.56 × 10^–3^
BFS_CA_6	0.16 M citric acid	6.4	21.549	1.03 × 10^–2^
BFS_TA_6	0.25 M tartaric acid	6.4	20.445	1.02 × 10^–2^

### 2.2 Analytical Methods

After the titrations, the suspensions were filtered through Pall 0.45 μm filters (Super 450, polyethersulfone, Ø 50 mm). The solids were washed with 100 mL of Milli-Q water, and then, the leachates were acidified to pH < 2 by adding concentrated HNO_3_ dropwise with a pipet. The concentrations of Si, Al, Fe, Ca, Mg, and Ti in the filtrates were analyzed using ICP-MS and the standard EN ISO 11885.^23^

Equation 1 can be used to evaluate if the release of element *i* is congruent with respect to the dissolution of Si
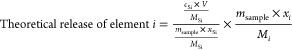
1where *c*_Si_ is the concentration of Si in the solution based on ICP-MS analysis (g/L), *V* is the final volume of the solution (L), *M*_Si_ and *M*_*i*_ are the molar masses of the Si and element *i* (g/mol), *m*_sample_ is the mass of the raw material (g), and *x*_*i*_ is the mass fraction of element *i* in the raw material based on XRF analysis (−). The first term after the equal sign indicates the Si moles in the solution divided by the total Si moles in the sample (i.e., it gives the estimation of the extent of the dissolution of the raw material, which is then used as a factor to calculate how many moles of element *i* should be dissolved in the case of a congruent dissolution).

Furthermore, the equivalent leached layer thickness of element *i*, noted as *e*_*i*_, is calculated by eq 2 as in ref (7)
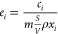
2where *c*_*i*_ is the concentration of *i* (g/m^3^), *m* is the mass of the raw material (kg), *S* is the surface area of the raw material (m^2^/kg), *V* is the final volume of the solution (m^3^), ρ is the density of the raw material (g/m^3^), and *x*_*i*_ is the mass fraction of element *i* in the raw material (−). The difference between the dissolved layer thickness of Si and the leached layer thickness of element *i*, for example, Ca, gives an estimation of the depth of the leached layer of the element.

### 2.3 Zeta Potential Measurements

The zeta potentials of the samples were measured using Zetasizer Pro Blue Label (Malvern Panalytical, UK) and ZS Xplorer software v.1.3.2.27. Details of the measurement are presented in the Supporting Information. The zeta potential was measured 10–30 min after the end of the titration experiments. As these raw materials do not remain stable under the experimental conditions and constant proton–metal exchange reactions do occur, the pH of the samples at the time of the zeta potential measurement was higher than the target pH at the end of the titration. The increase of pH was most pronounced for the BFS samples with a target pH of 6.4, for which the pH increase was on the order of 1 pH unit. For the BG samples, the pH increase was on the order of 0.1.

### 2.4 X-ray Photoelectron Spectroscopy (XPS)

The filtrated solids were dried overnight at 90 °C and stored in a desiccator until XPS analysis. The Thermo Fisher Scientific ESCALAB 250Xi XPS System was used, using a monochromatic Al Kα X-ray source with an energy of 1486.68 eV. A pass energy of 150 eV and a step size of 0.5 or 1.0 eV were used for scans. The spectra were analyzed using Avantage software v.5.976. All of the spectra were calibrated by assigning the characteristic adventitious carbon C 1s peak energy to 284.8 eV.

### 2.5 Thermodynamic Modeling (PHREEQC)

The speciation of the aqueous species, the metal–ligand complexes, and the saturation indexes of the possible precipitates were calculated using PHREEQC thermodynamic modeling software. The PCHatches_18.dat database^24^ was used and modified by adding citrate and tartrate complexes from NIST46 – Critically selected stability constants of metal complexes, v.8.0.^25^ The temperature and ionic strength of the stability constants were 25 °C and 0.1, respectively, or the closest values available in the NIST46 database. The log *K* values for hydrolysis and complex formation reactions can be found in the Supporting Information (Table S2).

## 3 Results and Discussion

### 3.1 Element Concentrations in the Solution and the Leached Layer Thickness

**Figure 1:**
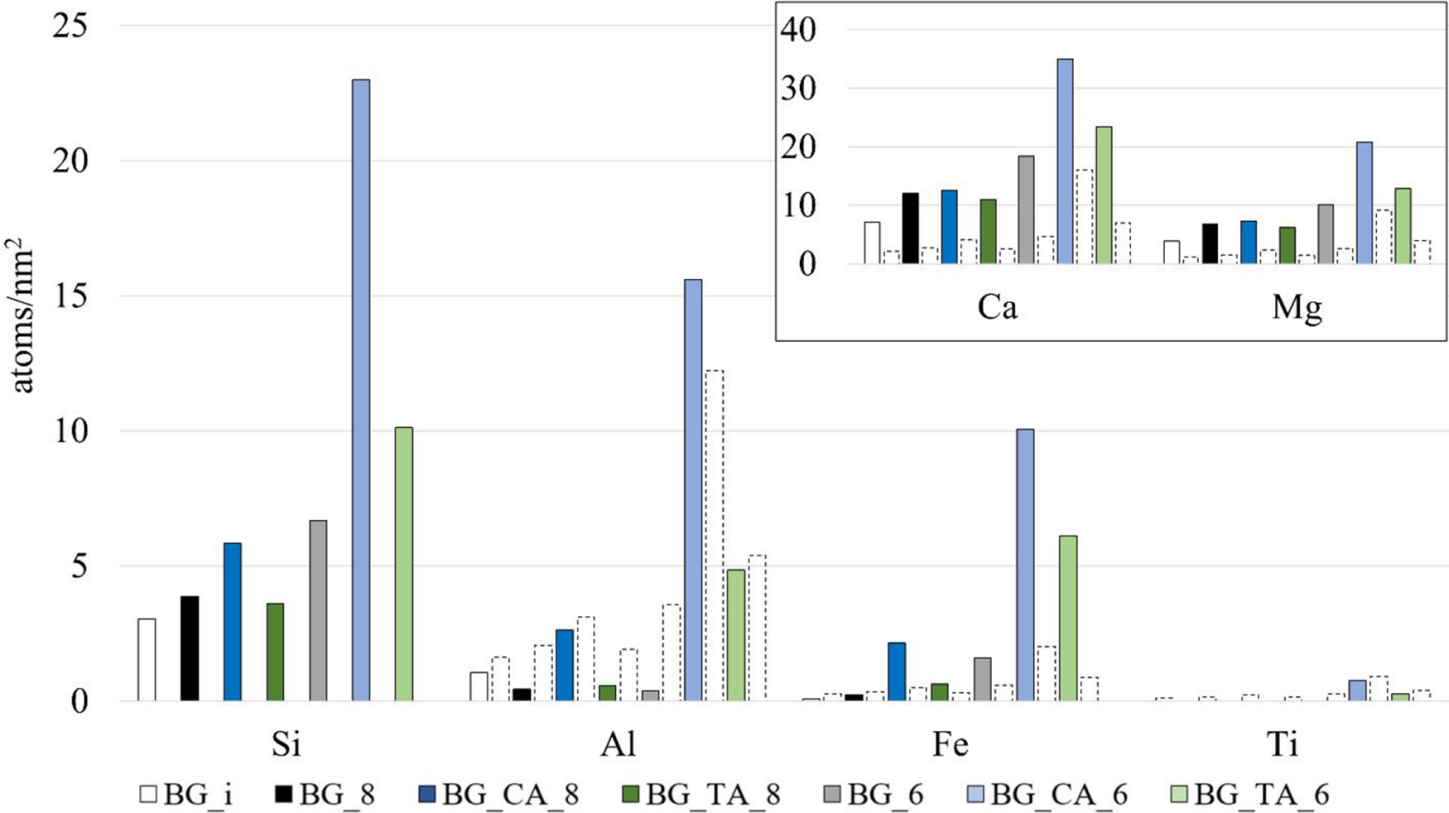
Element release during basalt glass titration experiments. The white columns with dashed outline on the right side of each colored column represent the theoretical release of the element, as calculated by eq 1. If the colored column is higher than the white dashed column, the element is released more in relation to Si, and if the colored column is lower than the white dashed column, the element is released less in relation to Si.

Figure 1 shows the element concentrations in the filtrate after basalt glass titrations. The results are normalized to the BET surface area of the raw materials. The release of Si is only slightly higher at pH 8 than at pH 10 (the pH of immersion, BG_i), which shows that the extent of the dissolution of the basalt glass is similar at pH 10 after 5 min of mixing (the mixing time of the BG_i sample) as it is at pH 8 after 124 min of mixing, demonstrating the low solubility of basalt glass at pH 8. Titration to pH 8 with citric acid (BG_CA_8) increases the Si dissolution by ∼50% in comparison to titration with HCl, whereas tartaric acid does not increase Si dissolution at pH 8 in comparison to HCl. Titration to pH 6.4 with HCl (BG_6) increases the Si dissolution ∼70% in comparison to the titration to pH 8 with HCl. An immense effect was observed by citric acid: the Si dissolution is over 300% higher with citric acid at pH 6.4 compared to HCl.

The Al concentration in the leachate was lower at pH 8 and 6.4 than at the pH of immersion with HCl, indicating an adsorption or a precipitation of Al on the glass surface at this pH range.^5,14^ With citric acid, the Al release is nearly congruent in relation to Si at pH 8, and at pH 6.4, Al is released proportionally more in relation to Si. A nearly congruent release of Al at pH 6.4 is observed also with tartaric acid, showing the clear effect of these acids for Al release at this pH.

The release of Fe is nearly congruent in relation to Si at pH 8 with HCl and tartaric acid, but over 400% higher Fe leaching is detected in the presence of citric acid. At pH 6.4, Fe is leached with all acids, but a significantly higher release is observed with citric and tartaric acids compared to HCl. A nearly congruent release of Ti was observed with citric and tartaric acids at pH 6.4, but Ti was not detected in any of the other filtrates.

High leaching of Ca and Mg was detected for all samples. The leaching of Ca and Mg is higher at pH 6.4 than at pH 8. The preferential release of alkaline earth cations at this pH range is consistent with the proton–metal exchange reactions, as explained in the Introduction. Citrate and tartrate increase the Ca and Mg concentration in the solution; however, the Ca/Si and Mg/Si ratios in the solution are *lower* with citrate and tartare than with HCl (Supporting Information, Table S2), which demonstrates that organic ligands could proportionally increase the Si and Al release and thus depict more closely congruent dissolution, or decrease Ca and Mg release, or increase the adsorption or precipitation of the Ca and Mg phases.

**Figure 2:**
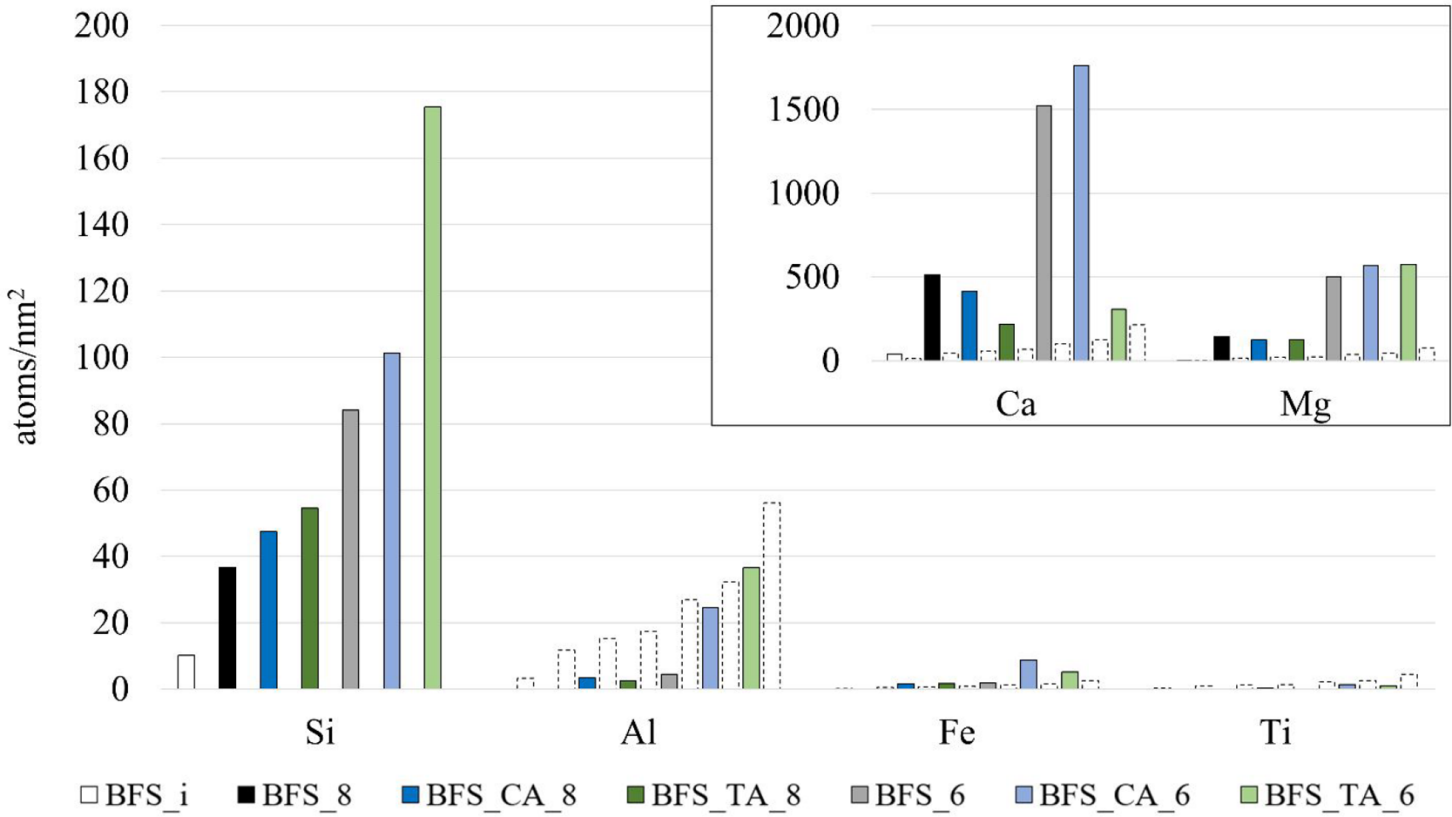
Element concentrations in the solution after blast furnace slag titration. The white columns with dashed outline on the right side of each colored column represent the theoretical release of the element, as calculated by eq 1. If the colored column is higher than the white bar, the element is released incongruently and more in relation to Si, and if the colored column is lower than the white bar, the element is released incongruently and less in relation to Si.

Figure 2 shows the dissolution and leaching of elements for the BFS samples. The extent of dissolution is significantly higher for BFS than for BG. The higher element release from BFS compared to BG is even more pronounced in the case of Ca and Mg, which can be explained by the lower degree of polymerization of the silica network. The degree of polymerization can be represented by NBO/T, the number of non-bridging oxygens (NBO) per tetrahedral-network-forming ion (T), as done previously with similar glass compositions.^26^ The NBO/T of BG and BFS are 1.2 and 2.4, respectively (Supporting Information, Table S1). BFS contains significantly more Ca and Mg and less Si compared to BG, which is depicted by the higher NBO/T value corresponding to a lower degree of polymerization in the silica network and to a higher extent of dissolution within the duration of the experiments.

Otherwise, similar trends in element releases are observed for both BFS and BG: element releases are higher at pH 6.4 than at pH 8, and citric and tartaric acid increase the Ca and Mg concentrations but decrease the Ca/Si and Mg/Si ratios in the solution. However, tartaric acid is more effective in increasing the extent of dissolution of BFS than citric acid, whereas the opposite is observed for BG. It is interesting to note that, even though the proton consumption for the BFS samples titrated to pH 6.4 was on the same level with all acids (Table 1), the extent of the dissolution with tartaric acid was higher. This demonstrates that tartaric acid enhances the dissolution process of BFS at this pH, which is not solely dependent on the proton–metal exchange reactions. Citric acid was not as effective in releasing Al from BFS at pH 8 as it was for BG. Similarly, Ti in the BFS samples was released only in the presence of citric and tartaric acids, which was also the case for the BG samples.

**Figure 3:**
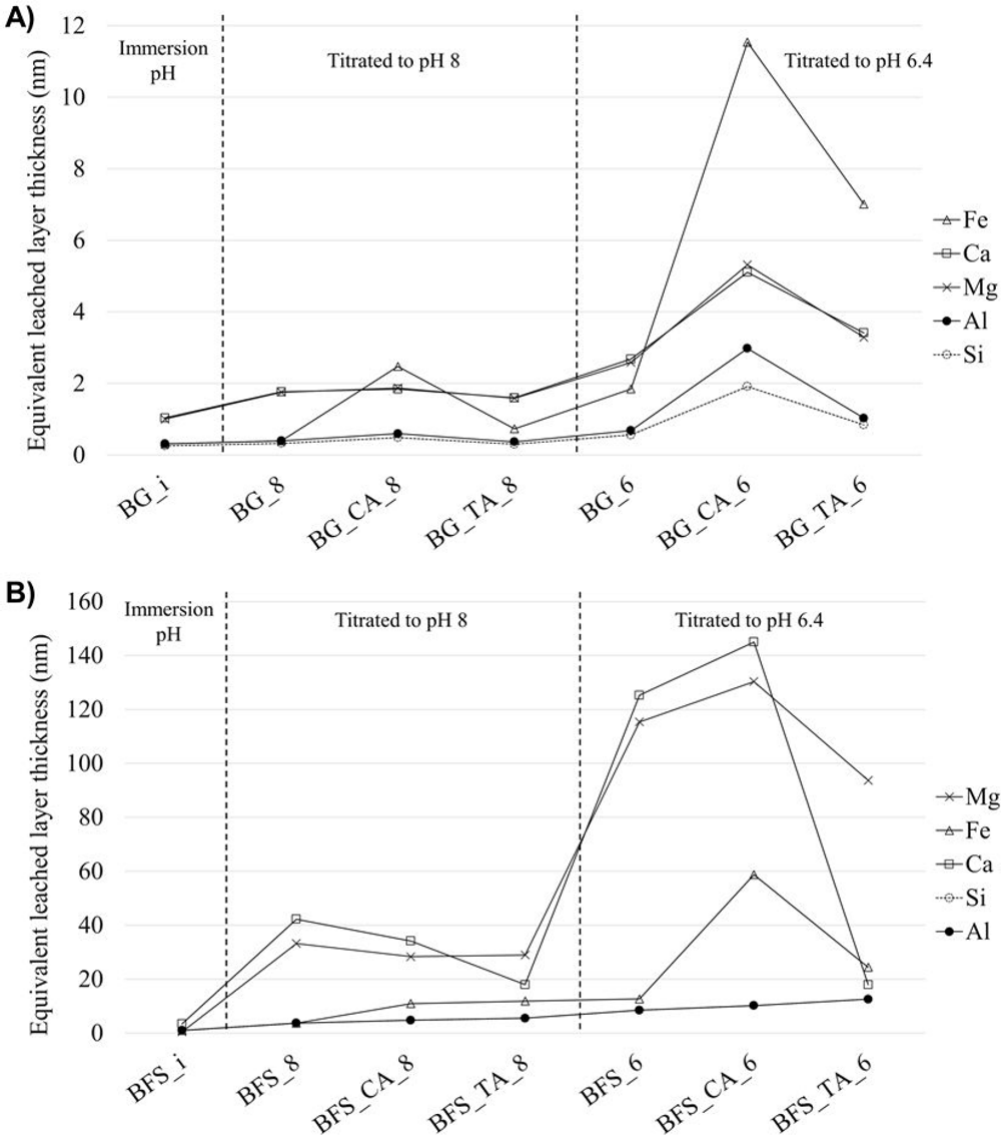
Equivalent leached layer thickness calculated according to eq 2.

The leaching and dissolution of elements can be recalculated into an equivalent leached layer thickness using eq 2. The equivalent leached layer thickness gives an indication of how deep from the raw material surface the elements have leached or dissolved. The results are shown in Figure 3. The elements are leached deeper from the surface with a more acidic pH. Citric acid strongly affects the leached layer thickness for Fe. The leached layer thickness of Ca and Mg has a similar trend for both raw materials—except for BFS with tartaric acid, for which the calculated leached layer thickness is lower for Ca than for Mg. The low calculated leached layer thickness for Ca is likely due to the precipitation of Ca phases, which will be discussed in later sections. The adsorption and precipitation of the element and the consequent lower element concentration in the leachate will affect the calculations based on eq 2. Thus, for example, the low leached layer thickness of Al does not represent the actual leaching depth of Al due to the readsorption and precipitation of Al on the silicate surface.

The maximum thickness of the leached layer for BFS is ∼140 nm, whereas, for BG, the maximum depth is only ∼12 nm, clearly showing how the raw material composition affects how deep from the surface the proton–metal exchange reactions can leach elements. As explained earlier, BFS consists of a less polymerized silica network compared to BG. The cation leaching creates continuous channels between the parallel chains of Q^2^ Si units (using the Q^*n*^ notion, where *n* = 0–4 refers to the connectivity of the SiO_4_ tetrahedral unit), which favors the diffusion of aqueous protons and water in the structure.^27,28^ Snellings^6^ observed similar leached layer thicknesses for Ca and Mg as reported here using HCl and silicate glasses with a similar composition as BG in this study, giving confidence for the replicability of the experiments.

### 3.2 XPS

#### 3.2.1 Element Ratios on the Surface

**Figure 4:**
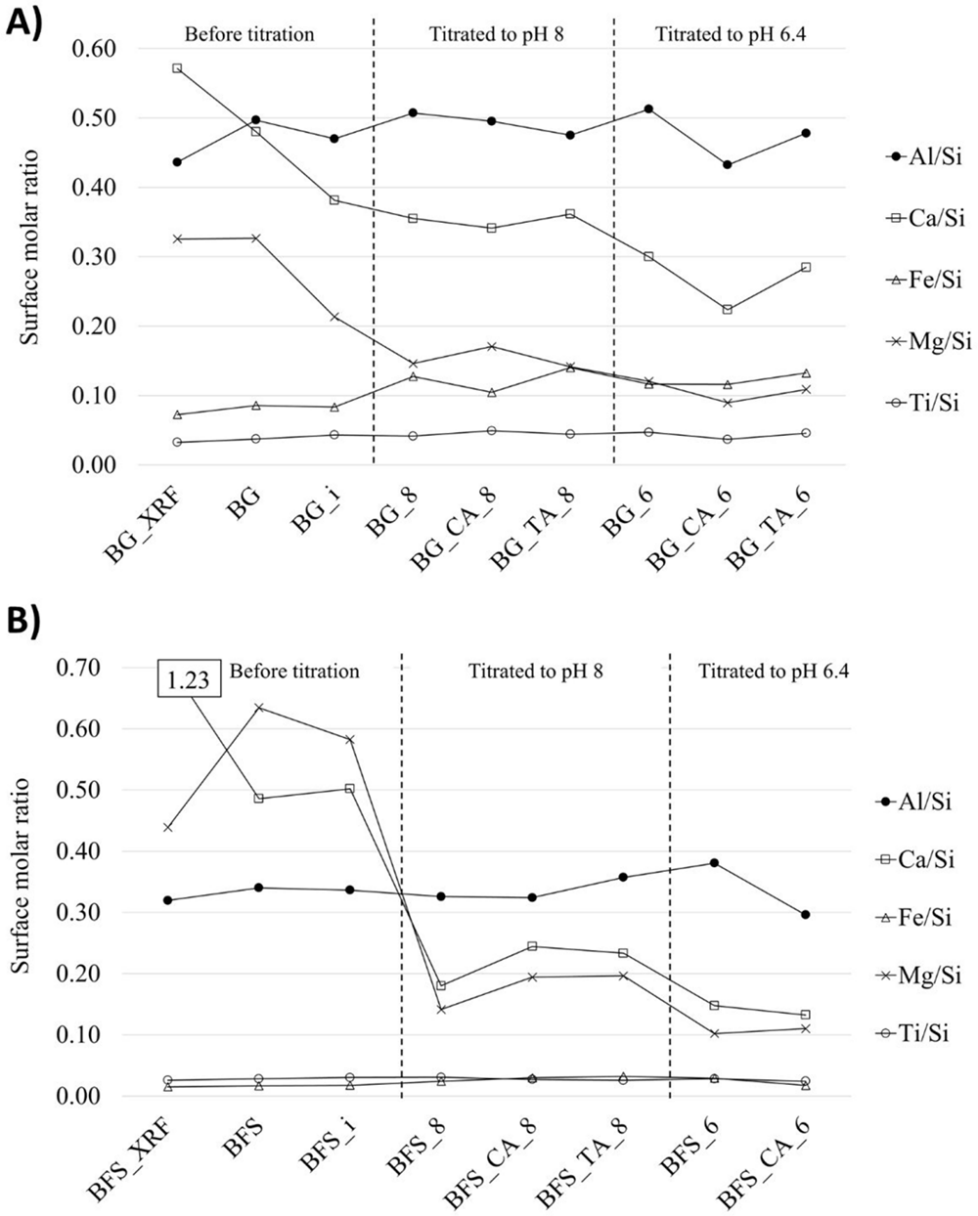
XPS analysis of the surface of basalt glass blast furnace slag before and after the titration experiments. “BG_XRF” and “BFS_XRF” indicate the molar ratio calculated based on XRF analysis. Sample BFS_TA_6 was not analyzed. The Ca/Si ratio of BFS_XRF was 1.23 (i.e., outside of the plotted graph and thus marked as “1.23” in the text box).

The surface composition of the samples was analyzed by XPS, and the molar ratios (at. %) of the elements in relation to Si were calculated (Figure 4). The XPS method gives very sensitive compositional information about the ratios of different elements at the surface (i.e., ≤10 nm). The molar ratios of the bulk of the material were calculated based on the XRF data named as “BG_XRF” and “BFS_XRF”. The full XPS data sets are provided in the Supporting Information (Table S4).

There is less Ca and more Al on the surface of BG compared to its bulk, whereas other elements are present in similar concentrations on the surface and in the bulk. Also, in the case of BFS, there is a difference in the bulk and surface Ca/Si and Mg/Si ratios. At the immersion pH, the surface Ca/Si, Mg/Si, and Al/Si ratios decrease, in line with the ICP-MS results, which showed leaching of Ca, Mg, and Al. The further titration to pH 8 with HCl increases the Al/Si and Fe/Si surface ratios of BG, indicating adsorption and/or precipitation of Al and Fe on the surface. In contrast, the Ca/Si and Mg/Si ratios decrease, indicating increased leaching to the solution. At pH 8, citric acid slightly decreases the Al/Si and Fe/Si surface ratios and increases the Mg/Si ratio. An increase in the Ca/Si ratio and Mg/Si ratio of certain samples with citrate and tartrate is observed, indicating the adsorption or precipitation of Ca and Mg on the surface of those samples. Another possible explanation for the increased Ca/Si and Mg/Si ratios would be a decreased concentration of Si on the surface; however, as lower concentrations of Ca and Mg in solution were determined by ICP-MS (Figures 1 and 2), the adsorption of Ca and Mg is a more likely explanation.

After the titration to pH 6.4 with HCl, the surface Al/Si ratio increases as a sign of more pronounced Al adsorption and/or precipitation on the surface. In contrast, Ca and Mg are leached to a higher extent, while the Fe/Si ratio is not affected significantly by a pH change between 8 and 6.4 with HCl. A strong effect by citric acid is observed at pH 6.4, which decreases the Al/Si ratio, a result that is in line with the high Al concentration in the filtrate based on the ICP-MS results. Moreover, Ca and Mg are present in lower concentrations on the surface in the presence of citric acid.

The Ti/Si ratio between pH 10 and 6.4 is in the range of 0.03–0.05 and 0.02–0.03 for BG and BFS, respectively. There is a slight decrease in the Ti/Si ratio with citric acid at pH 6.4 for both raw materials, suggesting that citric acid can influence Ti release at pH ∼6.4, which is in line with the ICP-MS results.

#### 3.2.2 High-Resolution XPS Spectra

**Figure 5:**
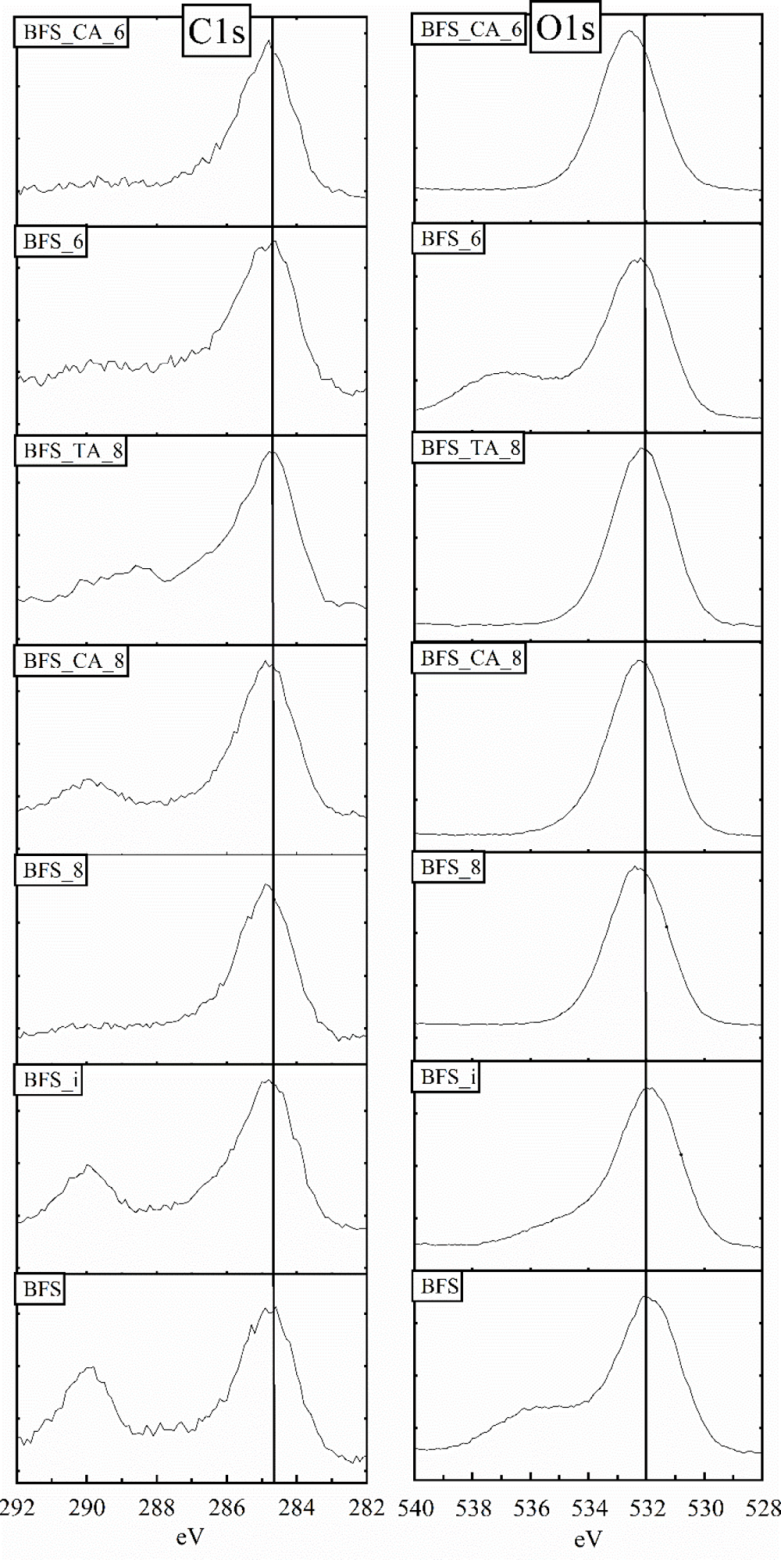
XPS C 1s and O 1s spectra of the BFS samples. The vertical lines are added to guide the eye across the spectra.

Figure 5 show the XPS C 1s and O 1s spectra of the BFS samples. XPS C 1s and O 1s spectra for BG samples are provided in the Supporting Information (Figure S3). The peak at 284.8 eV of the C 1s spectra is present in all of the samples caused by adventitious carbon. The C 1s spectra of both BFS and BG show a carbonate peak at 290 eV,^29^ which is consistent with the CaCO_3_ identified by the XRD analysis (Figure S1). As the carbonate signal for BG is weak and CaCO_3_ was not detected by XRD, the possible CaCO_3_ content on the BG surface is low. The short immersion of BFS in water did not dissolve all of the CaCO_3_ from the BFS surface, as the carbonate signal is still present in the C 1s spectra of BFS_i. In contrast, the carbonate signal is not present in the C 1s spectra of the BG_i sample, indicating the dissolution of the CaCO_3_ traces from the BG surface.

When BFS is titrated to pH 8 with HCl, the carbonate signal at ∼290 eV disappears, indicating the dissolution of CaCO_3_. It is noted that the dissolution of carbonate will increase the overall proton consumption of the samples titrated to pH 8 (Table 1). A signal at ∼290 eV is present for BFS_CA_8, but it is not likely that it would be due to the presence of CaCO_3_, as Ca leached extensively for this sample (Figure 2) and the carbonate signal was not detected in the BFS_8 sample. Instead, the signal at ∼290 eV could indicate the presence of carboxylate groups of citrate.^30,31^ BFS_TA_8, and BG_TA_8 to a lesser extent, exhibits a broad feature between 288 and 290 eV, which could be assigned to the carbon atoms in the O—C=O and C=O groups of tartaric acid.^30,31^ Moreover, a broad shoulder peak at 286.6 eV is present in the C 1s spectra of BFS_TA_8 and BG_TA_8, which could be ascribed to the carbon atoms in the C—OH groups of tartaric acid.^30,31^ The BG_CA_6 and BG_TA_6 samples show minor features around 289 eV, indicating the possible presence of citrate and tartrate on the surface. In addition, a shoulder peak at 285.6 eV in C 1s spectra of BG_CA_6 could be due to the CH_2_ groups of citrate.^32^ A table of functional groups in C 1s spectra and fitting for the BFS_TA_8 sample are provided in Supporting Information, Table S4.

The O 1s spectra of all samples show a peak between 531 and 532 eV, which can be ascribed to the oxygen found mainly in the silicate and aluminate groups.^33,34^ The broad feature observed at 536 eV in the O 1s spectra of BFS, BFS_i, and BFS_6 is expected to be due to the Na KLL Auger peak.^35^ A shift toward a higher O 1s binding energy is observed for the titrated samples, which is most pronounced for the BG_CA_6 and BFS_CA_6 samples. The increased O 1s binding energy can be explained by the interjection rule.^36^ Based on the raw material composition and minerology, Ca and Mg are the main network modifying elements in the raw material (i.e., most of the non-bridging oxygen bonds are Si–O–Ca and Si–O–Mg).^37^ As Ca and Mg are leached during the titrations, the remaining Si–O^–^ groups are (partly) balanced by protons, and Si–O–H bonds are formed. Due to the electronegativity differences^38^ between Ca, Mg, and H, the O–H bond is less ionic compared to the O–Ca or O–Mg bond, and thus according to the interjection rule, the binding energy of O 1s increases (refer to ref (36) and the Supporting Information in ref (39) for a more detailed rationale). The same is also true in cases where Al or Fe act as a network modifier. The overall cation/Si surface ratio is the lowest for BG_CA_6 and BFS_CA_6 (Figure 4); thus, the change of O–M bonds to O–H bonds is highest for those samples, and consequently, the increase in the binding energy of O 1s is highest for these samples.

**Figure 6:**
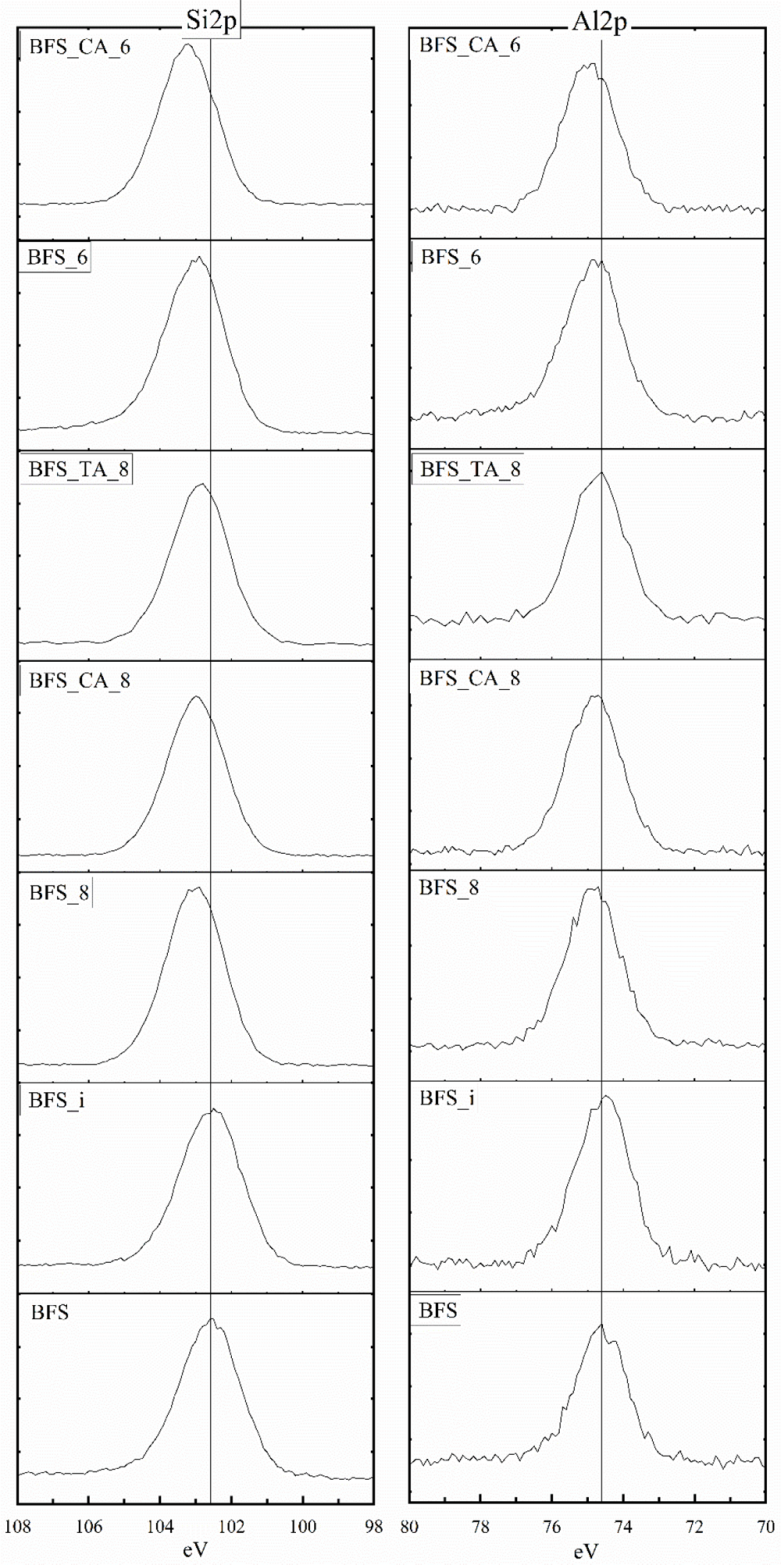
XPS Si 2p and Al 2p spectra of BFS before and after the titration experiments. The vertical lines are added to guide the eye across the spectra.

The Si 2p and Al 2p XPS spectra of the samples are shown in Figure 6 and Figure S4. The peak position of Si 2p is at 102.3 and 102.6 eV for BG and BFS, respectively, which is in good agreement with other multi-oxide silicate materials in the literature.^36,40^ The main forms of Si in both raw materials are Si bonded with bridging oxygen (Si–O–Si) and Si bonded with non-bridging oxygen (Si–O–M).

There is an increase in the binding energy of Si 2p for the titrated samples. The greatest shift observed for samples titrated with citric acid to pH 6.4. There are two possible explanations for the observed shift. During the experiments, the less polymerized Si species (Q^0^, Q^1^, and Q^2^) are liberated faster in comparison to their more polymerized (Q^3^ and Q^4^) counterparts.^41^ Consequently, the remaining surface would enrich in a higher polymerized Si as the dissolution proceeds forward. The Si 2p binding energy of each Si species is slightly different with more polymerized Si species being located at higher binding energy values.^42^ As a consequence, as the dissolution proceeds and the surface becomes enriched with more polymerized Si species, the Si 2p signal of the more polymerized Si intensifies, and the Si 2p signal of the less polymerized Si species diminishes.

Another possible explanation is that, during the titration, the metals on the surface of the Si–O–M groups are replaced by the proton–metal exchange reactions of >Si–O–H, >Si–O^–^, or >Si–OH_2_^+^, with the protonation state depending on the pH. The intense cation leaching creates continuous channels between the silicate units, which favors the diffusion of aqueous protons in the structure.^27,28^ As a result of the generated strains and intense hydration, the residual Si polymers collapse into a more compact structure, forming new Si–O–Si bonds, which would increase the Si 2p binding energy in comparison to the Si–O–Ca and Si–O–H structures. However, it is questionable if this could occur in the limited duration of the experiments.^6,43^

The main peak in the Al 2p spectra in all samples is around 74.4 eV, while a shoulder peak of 73.9 eV for BG, BFS, BG_i, BG_TA_8, and BG_6 is observed. After titration, a shift toward higher binding energy is observed, and the shoulder peak at ∼73.9 eV diminishes, particularly for samples with citric acid. In general, the Al 2p binding energy of tetrahedral Al is lower than that of octahedral Al, for which the binding energies are in the range of 73.2–74.4 and 73.9–74.8 eV, respectively.^44^ This is because the Al–O bond in an octahedral coordination is longer than that in a tetrahedral coordination; consequently, the Al–O bond in an octahedral coordination will be more ionic in nature and the binding energy higher.

In a previous study,^45^ Al was found to exist predominantly in a tetrahedral coordination in anhydrous stone wool (∼basaltic glass), but pentahedral and octahedral coordinated Al were also detected based on NMR spectroscopy. Al in anhydrous BFS can exist in tetrahedral and octahedral coordination.^46,47^ Houston et al. concluded that Al adsorbed on an amorphous silica surface is mainly tetrahedrally coordinated at pH 6.4–8.2.^14^ They proposed that tetrahedral Al adsorbs on >Si–OH sites as an inner-sphere bidentate coordination complex on amorphous silica, while the detected octahedral Al is due to the surface-enhanced precipitation of Al-hydroxides. It is noted that the shift observed here is only ∼0.4 eV, which casts doubt about the extent of the conclusion that can be drawn from this. However, a similar observation was made by Barr et al.^44^ They assumed that the reason for the minor shift is due to the amphoteric nature of gibbsite, which would mitigate the shift. In this study, the shift toward higher Al 2p binding energy for the samples with citric acid indicates an increased amount of octahedral Al and, consequently, an increased amount of Al-hydroxides on the surface.

Interpretation and discussion about Fe 2p spectra of the samples are presented in Supporting Information, Figure S2. Shortly, mostly Fe^2+^, but also some Fe^3+^, is detected on the surfaces of the samples after the titrations.

### 3.3 Zeta Potential Measurements

**Figure 7:**
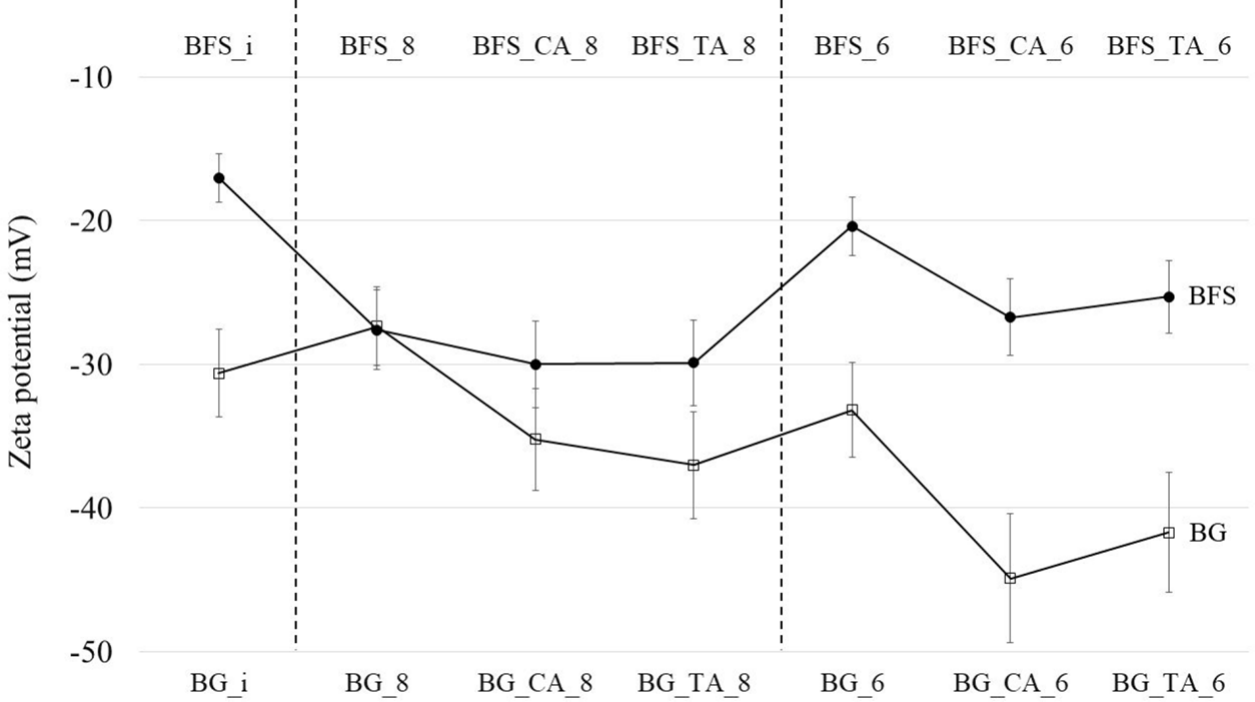
Zeta potentials of the basalt glass and blast furnace slag samples. The error bars presenting ±10% are based on the error of the standard samples of the zeta potential. The standard deviations of the triplicate measurements of the samples are less than the ±10% error. The pH of certain samples at the time of the zeta potential measurement was higher than the target pH at the end of the titration (please see section 2.3).

The zeta potentials of the samples after titration are shown in Figure 7. The measured zeta potentials depend on three main factors: (1) the type and extent to which cations are released to the solution and mainly formation of negatively charged >Si–O^–^ groups (also other surface groups such as >Al–O(H) will change the surface charge properties^5^); (2) the physical (re)adsorption of cations on the material surface; and (3) electrolyte composition (e.g., acid type).^48,49^ Overall, basalt glass has a more negative zeta potential compared to BFS, as the measured zeta potentials generally decrease with an increasing SiO_2_ content of glasses,^6^ although also >Al–O(H) surface groups contribute. As the Al content is higher in BG than in BFS, there are likely more >Al–O(H) surface groups on BG than on BFS and consequently a more negative zeta potential.

The zeta potential of BG is more positive at pH 8 than at pH 10 (BG_i), which indicates an adsorption of cations on the surface at pH 8, although the zeta potential would be more positive toward the isoelectric point even without specific cation adsorption.^5,6^ In contrast, the zeta potential of BFS is more negative at pH 8 than at pH 11.2 (BFS_i), which indicates a lower concentration of cations on the surface. Both observations are in line with the XPS results. The positive cations Al^3+^ and Fe^2+/3+^ are physically adsorbed and/or precipitated on the BG surface at pH 8 based on the higher Al/Si and Fe/Si ratios (Figure 4), thus creating a more positive zeta potential, whereas no change is detected in the Al/Si and Fe/Si ratios for BFS between pH 11 and 8. At the same time, the surface Ca/Si and Mg/Si ratios of BFS decrease drastically, consequently creating a more negative zeta potential.

The zeta potential is more negative for both materials if titrated with citric and tartaric acid than with HCl. The more negative zeta potential can be attributed to the combined effect of (1) lower cation/Si surface ratios for the citric and tartaric acid samples which consequently increase the number of negatively charged >Si–O^–^ groups and/or removal of >Al–O(H) surface groups due to dissolution; (2) the presence of negatively charged citrate^3–^ and tartrate^2–^ ions in the solution; and (3) the complexation of cations with citrate and tartrate in the solution, which could to some extent counteract the effect of electrolytes on the zeta potential.^49,50^

At pH 6.4, the zeta potential becomes more positive than that at pH 8 for both raw materials, which indicates that the strong specific adsorption of Al^3+^ and, to a lesser extent that of Fe^2+^, takes place on the negatively charged >Si–O^–^ groups, which is in accordance with the XPS results. Al^3+^ and Fe^2+^ ions can reverse the zeta potential more than the Ca^2+^ and Mg^2+^ ions due to their smaller hydrated radius and higher charge; thus, less Ca^2+^ and Mg^2+^ can be adsorbed on the surface compared to Al^3+^ and Fe^2+/3+^. The effect of citric and tartaric acid on lowering the zeta potential is more pronounced at pH 6.4 than at pH 8, which is again in line with the XPS data and thermodynamic modeling, as shown in the next section.

### 3.4 Thermodynamic Modeling (PHREEQC)

The adsorption of Al on the silica surface has been studied and modeled in detail, for example, in refs (5 and 14), and is thus not modeled here. The adsorption of aqueous Al on the silica surface can form stable surface complexes, which inhibits the extent of dissolution. However, the precipitation of gibbsite and other phases is crucial, particularly once the Al surface site coverage exceeds a certain limit (e.g., 8% in the case of amorphous silica^14^). Here, the thermodynamic calculations are used to determine how leached metal speciation and saturation indexes of solid phases change in the presence of citrate and tartrate.

**Figure 8:**
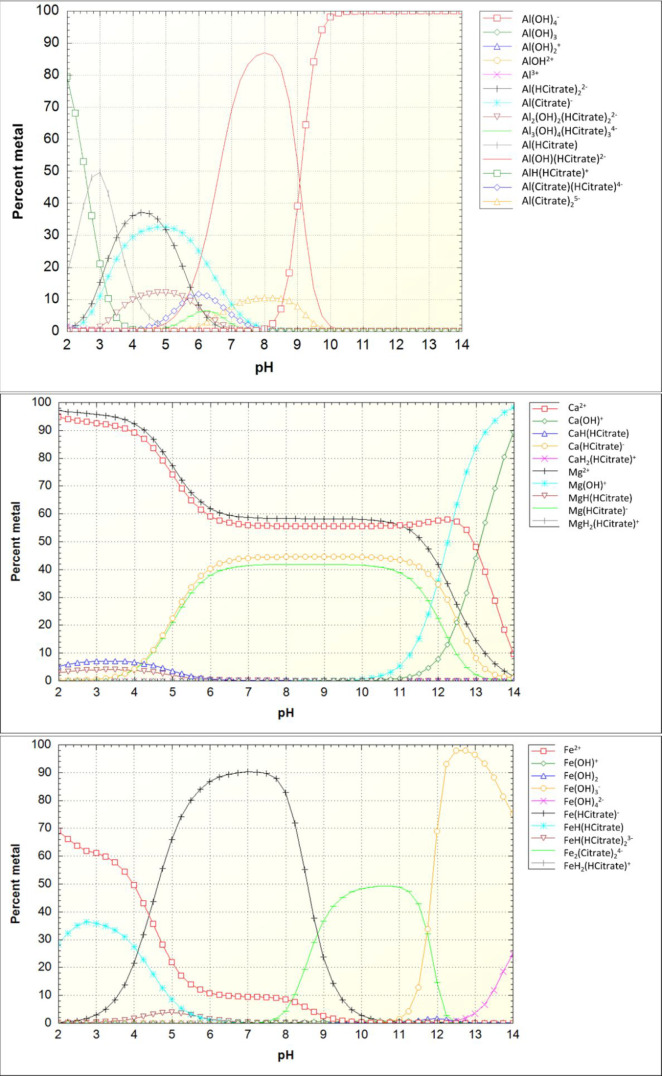
Metal speciation in the presence of citrate as a function of pH that is modeled with concentrations of the sample BG_CA_6. Total concentrations (as mmol/kg_water_): Al, 0.44; Ca, 0.99; Mg, 0.59; Fe^2+^, 0.28; citrate, 1.39.

**Figure 9:**
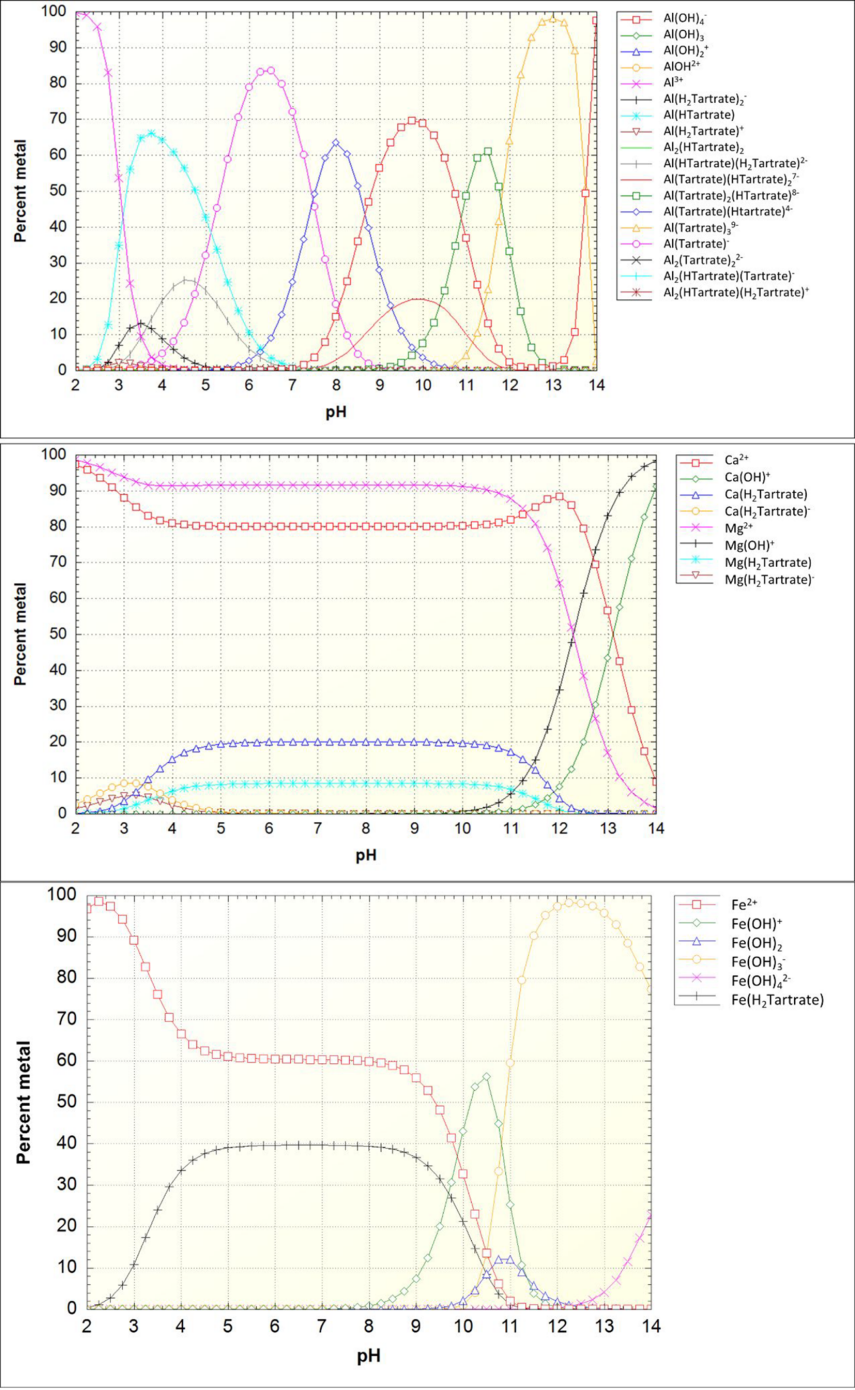
Metal speciation in the presence of tartrate as a function of pH that is modeled with element concentrations of the sample BFS_TA_8. Total concentrations (as mmol/kg_water_): Al, 0.72; Ca, 9.06; Mg, 5.23; Fe^2+^, 0.0736; tartrate, 22.9.

Figures 8 and 9 show the speciation of aqueous Al^3+^, Fe^2+^, Ca^2+^, and Mg^2+^ in the presence of citrate and tartrate as a function of the pH calculated according to the concentrations of elements in the leachates of the selected samples. Only Fe^2+^ was modeled, as it is not expected to have a significant amount of iron oxidized into Fe^3+^ during the relatively short experimental time. As seen in Figures 1 and 2, soluble iron is found as leached into the solution which would support the idea that iron is majorly (or solely) as Fe^2+^ and not as insoluble Fe^3+^. Citrate and tartrate chemistry with Al is diverse, and the NIST database notes several possible complexes for those.^25^ At a near-neutral pH, the Al(OH)(Citrate)^2–^ and Al(Tartrate)^−^ complexes are the most prevalent species for Al. Citrate can also form complexes with Fe^2+^ within the experimental pH, whereas for tartrate only one Fe^2+^ complex is acknowledged by the NIST database. The prevalent Fe^2+^–citrate complex formation is in line with the observed deep leached layer depth of Fe in the presence of citric acid.

The saturation indexes of citrate and tartrate salts were calculated with PHREEQC using the detected concentrations of ions present in the leachates, or if the release of ions is lower than congruent with respect to Si, then the theoretical concentration is used (eq 1). Based on the saturation indexes (Supporting Information, Figure S5), Ca_3_(HCitrate)_2_ and Ca- and Fe-tartrate salts could precipitate. The solution was supersaturated with respect to these complexes, but as no information about the precipitation kinetics is known, the modeling provides only the potential conditions for this. McKinnon et al.^51^ observed that the precipitation of Ca- and Fe-tartrates in supersaturated solutions was initiated within 4 min and reached its maximum rate after 2 h; thus, the precipitation of tartrate salts during the experiments conducted here is plausible. The precipitation of Fe-tartrate salt on the surface of the raw materials is supported by the high Fe/Si surface ratio and features around 288 eV in the C 1s spectra of the BG_TA_6 and BFS_TA_8 samples. Earlier work showed that the point of zero charge of similar materials is in the range from 7 to 9.^5,6^ At pH below the point of zero charge, the surface can be assumed to be positively charged and thus could attract negatively charged ligands and form surface complexes. However, formation of surface complexes or precipitation of citrate and tartrate salts cannot be exclusively confirmed by the experiments.

Also, the saturation indexes of selected solid phases were calculated with PHREEQC (Figure S5). As citric and tartaric acid favor the formation of soluble Al complexes, they decrease the saturation indexes of gibbsite and amorphous Al(OH)_3_. In the case of BG, which has a higher Al_2_O_3_ content, amorphous Al(OH)_3_ and gibbsite could precipitate based on the thermodynamical calculations despite the presence of citrate and tartrate. Based on the XPS Al 2p spectra, an increase in the octahedral Al coordination occurs for these samples, which is the coordination state of Al in gibbsite, thus supporting the thermodynamic modeling results. Houston et al.^14^ also observed the precipitation of the aluminosilicate phases based on NMR spectroscopy, which would also be likely in this study due to the similar experimental conditions. Moreover, due to the more complex chemistry involved here, the formation of several different Ca-, Mg-aluminosilicate phases, or their amorphous counterparts, is possible. The full saturation index list of phases included in the PCHatches_18.dat database is included in the Supporting Information (Table S5).

### 3.5 Aqueous Solution Chemistry

The dissolution of multi-oxide silicate glasses at a near-neutral pH is governed by the proton–metal exchange reactions. Equation 3 shows the leaching of Ca from a simplified composition of BG:
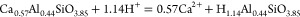
3In a similar way, Mg is leached. The leaching of Ca and Mg would form new surface >Si–O(H) groups and a Ca- and Mg-poor surface layer, with the depth and surface group protonation state depending on the pH, glass structure, and duration of the reaction. Simultaneously but at a lower rate, Al is leached:
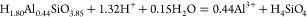
4In a similar fashion, Fe can be leached out—the dissolution rate depending on if it is present as a network modifier or as a network former in the silicate structure. The released Al, Fe, Ca, and Mg form a pseudoequilibrium involving the elements present in the bulk of the glass, the elements present in the alteration layer of the glass, and the elements present in the solution. The surface >Si–O^–^ and >Si–OH groups attract cations—particularly Al^3+^ and Fe—due to their small radius and high charge—consequently stabilizing the surface silica groups and inhibiting dissolution. The adsorption of Al on the silanol sites is proposed^14^ to follow eq 5:
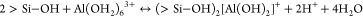
5Moreover, surface-enhanced precipitation of Al-hydroxide follows eq 6:
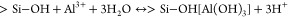
6In addition, the bulk precipitation of aluminosilicate phases is likely under these conditions^14^ and many aluminosilicate phases are supersaturated under these experimental conditions (Supporting Information, Table S5). In a similar fashion, Fe could adsorb on the silica surface, form Fe-silicates, or precipitate as Fe-hydroxide through a surface-enhanced precipitation mechanism.

The addition of citrate and tartrate changes the reactions shown in eqs 5 and 6 and the precipitation reactions of aluminosilicates through forming soluble complexes with aqueous Al,^25^ for example, as in the following:
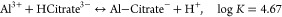
7
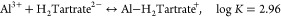
8The Al-complex formation decreases the effective concentration of available Al^3+^ for reactions in eqs 5 and 6 and for precipitation of aluminosilicate phases. As the extent of the dissolution of multi-oxide glasses is inhibited by these reactions at a near-neutral pH, based on the results presented here and in the literature,^5,11,14^ an increase in the extent of dissolution is observed when citrate and tartrate are present, as they shift the direction of the reactions in eqs 5 and 6 on the left-hand side of the equations. In a similar fashion, leaching, adsorption, precipitation, and complex formation reactions involving other cations can influence the extent and rate of dissolution. For example, Ca may stabilize negatively charged silica surface groups:
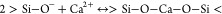
9By decreasing the effective concentration of aqueous Ca^2+^, the reaction in eq 9 proceeds on the left-hand side of the reaction, thus labilizing >Si–O^–^ groups and increasing the extent of dissolution.

In addition, both organic ligands can form insoluble Ca-salts, which can possibly precipitate on the surface of the raw materials. This is likely occurring for BFS for its higher Ca content compared to BG. Based on ICP-MS, the surface Ca/Si ratio, the XPS C 1s spectra, and the thermodynamic modeling, Ca-tartrate salt precipitated on the surface of BFS. However, the extent of dissolution of BFS is nevertheless the highest for samples with tartrate, which indicates that the Ca-tartrate salt precipitation does not inhibit BFS dissolution under these experimental conditions but tartrate is beneficial to increase BFS dissolution by its higher Ca-complexing capability (Figures 8 and 9).

## 4 Conclusions

The effects of citric and tartaric acid on the dissolution and surface alteration layer of basalt glass (BG) and blast furnace slag (BFS) at a near-neutral pH were investigated using batch titrations. The results demonstrated that the leached Al and Fe are adsorbed or precipitated as hydroxides or silicates on the surface of the multi-oxide raw materials based on the XPS analysis and thermodynamic modeling. In the presence of citrate and tartrate, soluble Al^3+^ and Fe^2+^ complexes are formed, consequently increasing the Al and Fe concentrations in the solution and decreasing the Al/Si and Fe/Si ratios in the surface alteration layer of the raw materials. In the presence of citrate, the Al/Si and Fe/Si ratios in the alteration layer become close to the stoichiometric composition of the bulk of the glass. Moreover, tartrate decreases Al/Si and Fe/Si ratios in the alteration layer but to a lesser extent compared to citrate. The lower overall surface cation/Si ratio, which was observed also as a more negative zeta potential, labilizes silicate groups and promotes dissolution of the raw materials. Up to a 300% increase in the extent of dissolution of these materials was observed by the organic ligands within the 2 h duration of the experiments. In the case of the Al-rich raw material (here BG), the main influence is gained by adding citrate, which can form soluble complexes with Al and limit the effective concentration of Al for surface reactions. Despite the presence of citrate, octahedrally coordinated Al precipitated as amorphous hydroxide on the surface of BG. However, for Ca-rich BFS, a higher impact is gained by tartrate, which can form Ca complexes. Although Ca-tartrate salt precipitated on the surface of BFS based on XPS data and thermodynamic modeling, this did not inhibit the further dissolution of BFS under these experimental conditions. Overall, the effect of citrate and tartrate on the Ca/Si and Mg/Si ratios in alteration layer ratios is lower compared to those of Al/Si and Fe/Si; instead, an increase in Ca/Si and Mg/Si ratios is detected for certain samples. To conclude, it was shown that there is a synergistic effect by protons, H_2_O, and organic ligands in the dissolution process of multi-oxide silicates. These results deepen the understanding of the surface behavior of multi-oxide glasses in the presence of organic ligands, which is important for many applications and illuminates not only the chemical interactions between the bulk glass, alteration layer, and solution but also how organic ligands affect the chemical reactions involved.

## Research Data

Research data associated with this article can be accessed at 10.23729/132faa37-0917-4a7f-a0a9-9199be7a0f74.

## Supporting Information Available

XRF composition, log *K* constants, element ratios in solution, XPS data, aqueous speciation and saturation indexes, XPS data, and XRD graphs (PDF)
