# Supplementary material for: Surface Layer Alteration of Multi-Oxide Silicate Glasses at a Near-Neutral pH in the Presence of Citric and Tartaric Acid
Source: Langmuir. 2022 Jan 13;38(3):987–1000. doi: 10.1021/acs.langmuir.1c02378 (PMC8793144; doi:10.1021/acs.langmuir.1c02378)
Supplement: Supplementary file 1 — la1c02378_si_001.pdf [file la1c02378_si_001.pdf]

Supporting Information for

Surface Layer Alteration of Multi-Oxide Silicate Glasses at a Near-Neutral pH in the Presence of Citric and Tartaric Acid

Juho Yliniemi[a*]

[a]Fibre and Particle Engineering Research Unit, Faculty of Technology, Pentti Kaiteran katu 1, 90014 University of Oulu, Finland.

* juho.yliniemi@oulu.fi

Titration experiment details:

The automated titration parameters of the Mettler Toledo T5 were the following: a pre-stir of 10 s, a pre-dispense of 0.0001 mL, a minimum dosage of 1 µL/min, a maximum dosage of 0.01 mL/min and 1 mL/min for BG and BFS, respectively, a pH control band of 0.001 pH units, and a stirring speed of 30%. The samples reached their target pH in 1-20 minutes, depending on the sample, after which the automation kept the pH at the target pH by adding small increments of acid as the pH rose due to the metal-proton exchange reactions. The duration of the titration experiments was 124 mins, after which the mixing was stopped, and the sample was removed for further analysis. Prior to experiments, the pH electrode was calibrated using the standard solution with a pH of 4, 7, and 10. The water and cell were purged with N_2 gas for 5-10 minutes prior to the experiments, and then the cell was opened, and the raw material was quickly added.

Zeta potential measurement details:

After the titration, 1 mL of the sample suspension was diluted with 14 mL of Milli-Q water to obtain a suitable particle concentration for the zeta potential measurement. The elements leaching from the raw materials during the titrations created a specific ionic strength, which increased with a lower pH due to the addition of acids and the leaching of elements, but no additional background electrolytes were used in the measurements to avoid complications with the experiments. The water-diluted suspensions were manually mixed and transferred to a DTS 1070 disposable folded capillary cell. Zeta potential measurements were carried out as triplicates at a constant temperature of 25°C, with a temperature equilibration time of 120 s and a 60 s pause between each measurement, using automatically optimized measurement parameters and a general-purpose analysis model. The calculation of the zeta potential was carried out using Smoluchowski's theory, which was adapted to electrophoretic light scattering measurements. The zeta potential was averaged over 3 subsequent measurements taking ~7 min in total.

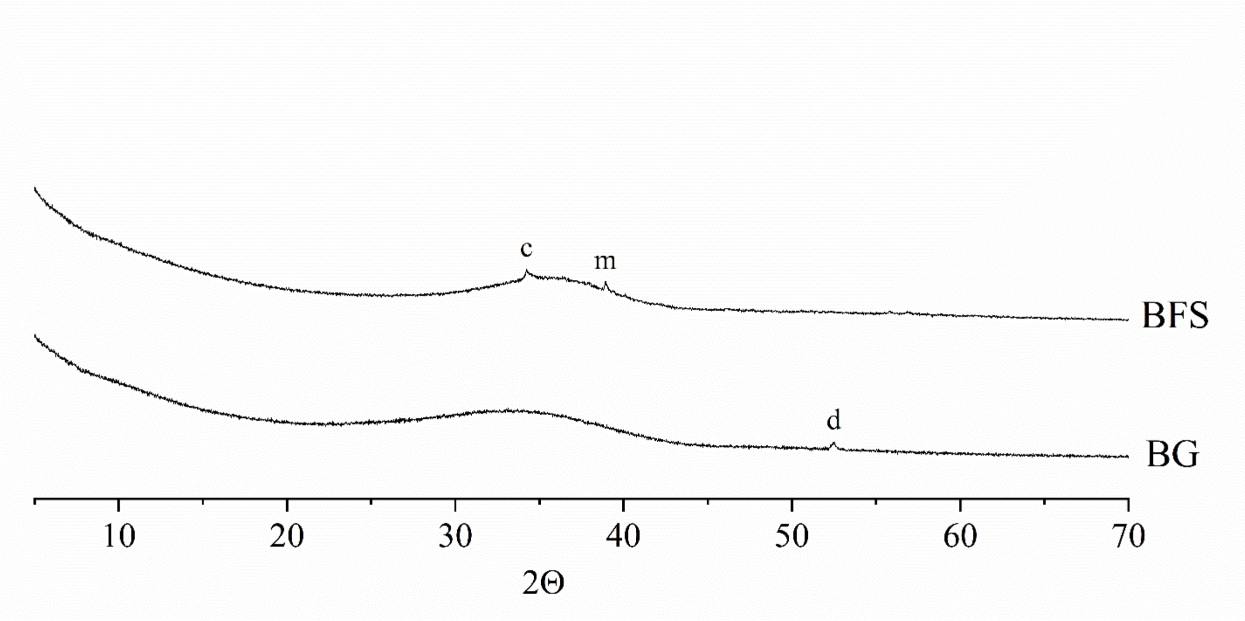

Figure S1. The XRD patterns of the raw materials. The main signals of certain minerals are marked with different letters – c: calcite, pdf# 04-077-2808; m: merwinite, pdf# 00-035-0591; d: diopside, pdf# 01-076-2531 or spinel, pdf# 04-005-7126.

XRD-analysis:

BFS contained traces of calcite ($CaCO_3$), which had likely formed during the storing of BFS[1,2], and merwinite ($Ca_3Mg(SiO_4)_2$), which is sometimes detected in BFS[3]. The x-ray diffractogram of basalt glass contained a weak signal at 52° 2θ, which could be either diopside ($CaMgSi_2O_6$) or spinel ($MgAl_2O_4$); both have been identified in the stone wool samples[4,5], but the identification was uncertain, as only this one signal at 52° 2θ was detected.

XPS Fe2p spectra:

The relatively low signal to noise ratio in the Fe2p spectra for BG – and particularly for BFS – is due to the small concentration of Fe in the raw materials. Nevertheless, it is possible to acquire information on the oxidation state of Fe with the help of charge transfer satellite signals[6]. The $Fe2p_{3/2}$ signal for Fe^{2+} is between 709.1 eV (for FeO) and 710.8 eV (for olivine), while the $Fe2p_{3/2}$ signal for Fe^{3+} is between 710.2 eV (for FeOOH) and 712.3 eV (for andradite)[6,7]. The $Fe2p_{3/2}$ satellites for Fe^{2+} and Fe^{3+} are at 715 eV and 719 eV, respectively[6]. In general, the Fe2p3/2 binding energies in silicate minerals are higher than in Fe-oxides[6,7]. The wide hump around 725 eV is due to $Fe2p_{1/2}$ signal of Fe^{2+} and Fe^{3+} [6].

The most intense signal for BG is at 711.8 eV, but a broad shoulder peak is also visible at ~715 eV. Therefore, the Fe2p spectra indicate that the Fe present on the BG surface is mainly Fe^{2+}, which is in line with the thermal history of BG[8]. The XPS spectra of BG_i remain similar as that of BG indicating no change in the Fe oxidation states during the 5 min immersion in water. However, the spectra of BG_8, and particularly those of BG_CA_8 and BG_TA_8, exhibit narrower shape around its main signal at 711.8 eV due to lower intensity of $Fe2p_{3/2}$ satellite peak around 715 eV. The disappearance of charge transfer satellites from the spectrum indicates presence of both Fe^{2+} and Fe^{3+} as reported in an earlier study[6]. For BG_6 and BG_CA_6, the charge transfer satellite at 715 eV appears again (more pronounced for BG_CA_6), but not for BG_TA_8, indicating that the Fe^{2+} is the main form present in BG_6 and BG_CA_6, but a mix of both Fe^{2+} and Fe^{3+} are present in BG_TA_6.

Regarding BFS, previous studies[9,10] have found that Fe in BFS is mainly present as Fe^0, for which the binding energy value is between 706.3 and 707.7 eV[6]. However, the signal intensity on that range is low in this study, meanwhile the main signal is at ~711 eV, with a features also at ~715 eV and ~725 eV, which indicates that the Fe detected here is in oxidized form, for example as FeS or within the silicate network, rather than as metallic Fe[11]. This is not necessarily in contradiction with earlier studies as the Fe^0-rich particles could still possess most of the mass of the Fe detected by XRF analysis. Both oxidation states of Fe are present in other titrated BFS samples – with a pronounced concentration of Fe^{2+} in samples BFS_CA_8, BFS_TA_8, and BFS_6 indicated by the more distinguishable charge transfer satellite signal at 715 eV.

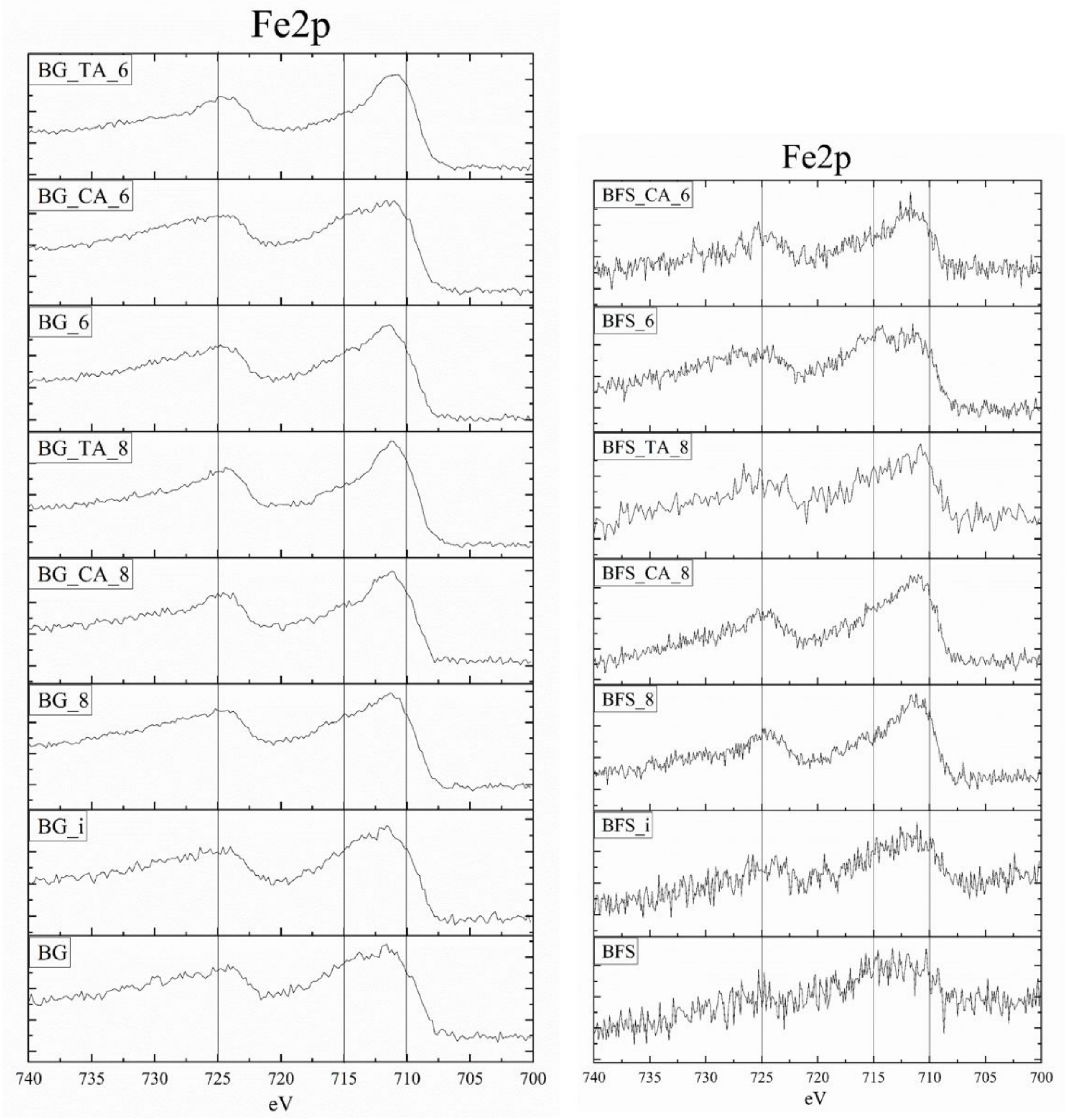

Figure S2. The Fe2p XPS spectra of BG. Vertical lines at 710 eV, 715 eV, and 725 eV are shown to guide the eye across spectra. Sample BFS_TA_6 was not analyzed.

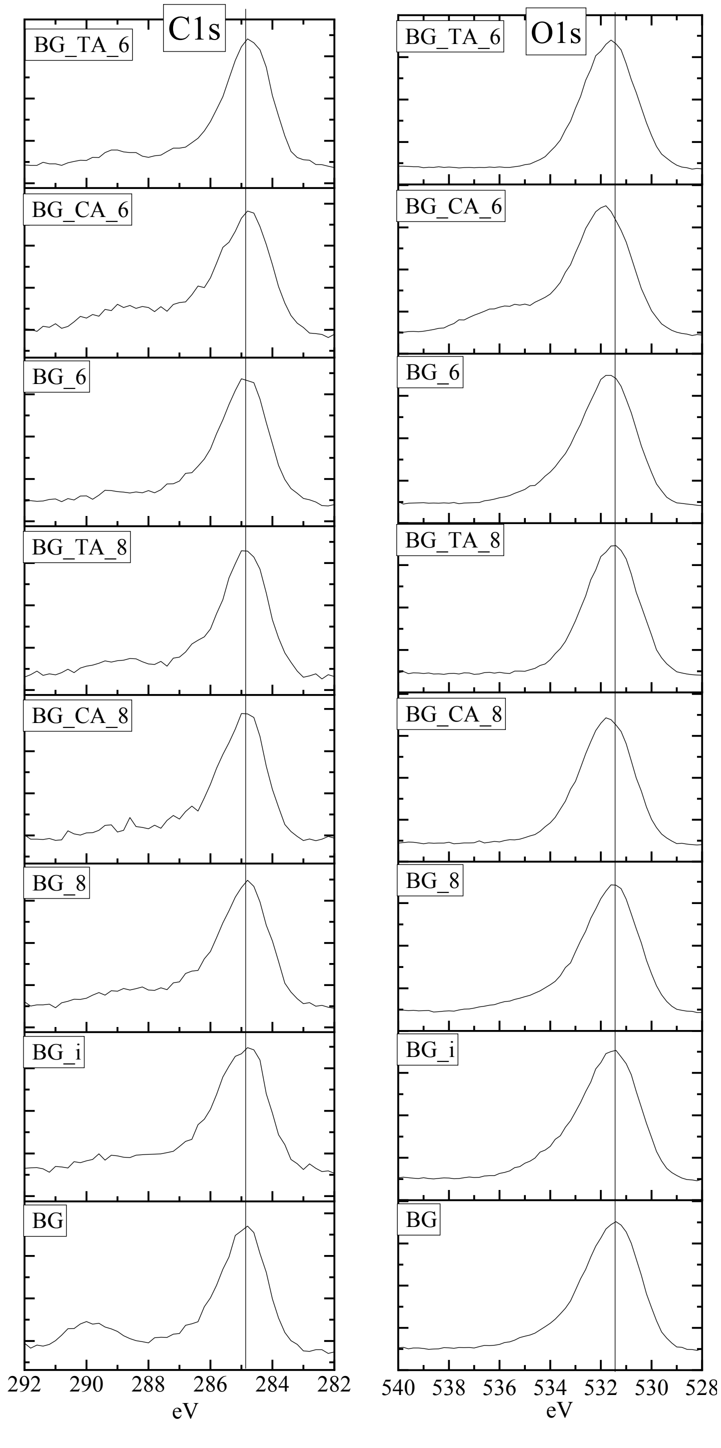

Figure S3. XPS C1s and O1s spectra of the BG samples. The vertical lines are added to guide the eye across the spectra.

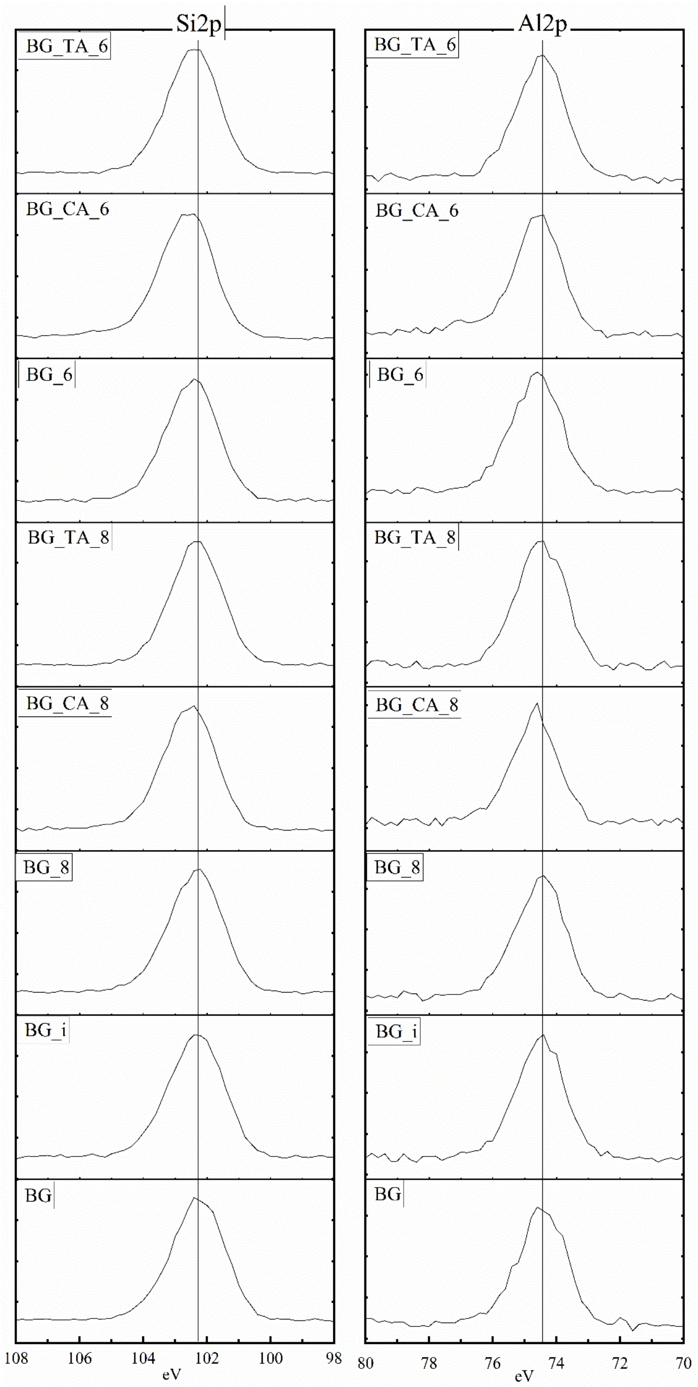

Figure S4. XPS Si2p and Al2p spectra of BG before and after the titration experiments. The vertical lines are added to guide the eye across the spectra.

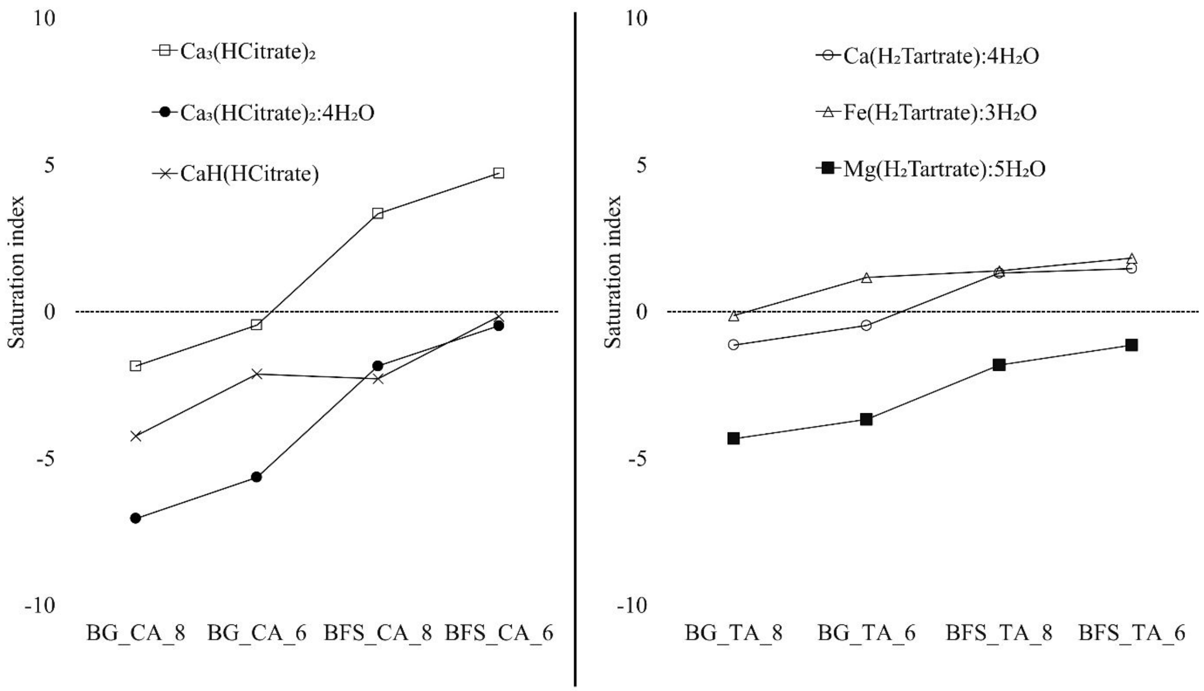

Figure S5. The saturation indexes of citrate and tartrate salts for the samples calculated with PHREEQC.

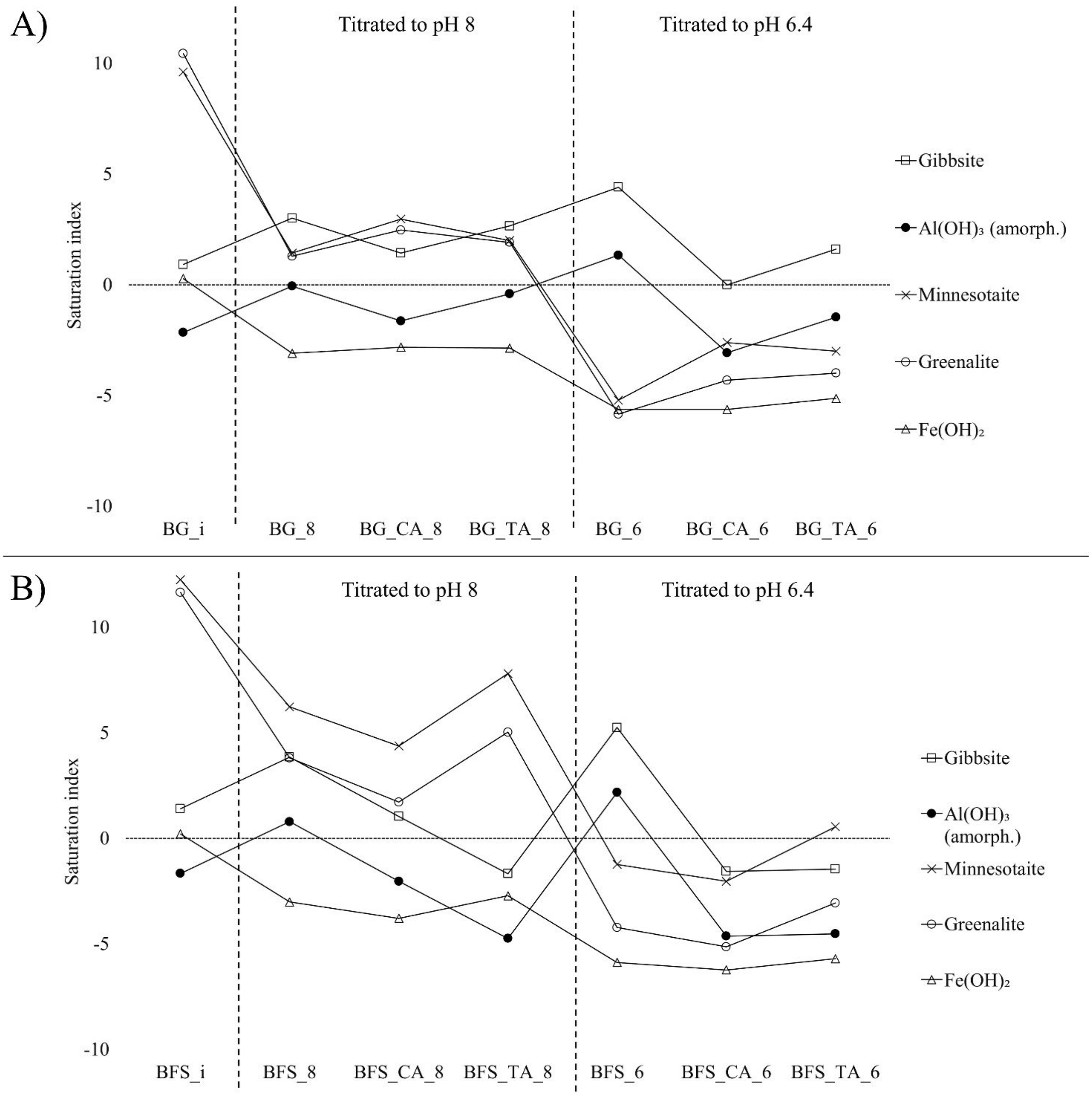

Figure S6. The saturation indexes of selected solid phases for the samples calculated with PHREEQC.

The saturation indexes of minnesotaite and greenalite – two Fe-silicate minerals that have been observed[12] to precipitate in sorption experiments at a similar pH as used here. The saturation indexes of

both phases are positive at pH_i and pH 8; thus, the precipitation of those, or their amorphous counterparts, would be possible based on the thermodynamic calculations. $Fe(OH)_2$ is not expected to precipitate, except in alkaline conditions. However, that XPS analysis indicated traces of Fe^{3+} in some samples, which would precipitate as $Fe(OH)_3$, FeOOH, or as their amorphous counterparts at this pH range. Nevertheless, Fe^{3+} is expected to represent only a trace amount of the total dissolved Fe.

Table S1. Chemical composition and physical properties of basalt glass (BG) and ground granulated blast furnace slag (BFS). The iron content is reported in both samples as Fe2O3 for easier comparison with the materials used in previous studies.

	**SiO_2**	**Al_2O_3**	**Fe_2O_3**	**CaO**	**MgO**	**Na_2O**	**K_2O**	**TiO_2**	**SO_3**	**Density (g/cm^3)**	**BET (m^2/g)**	**Surface area to solution volume (m^{-1})**	**NBO/T***
**BG**	41.1	15.2	4	21.9	9	2.7	0.7	1.8	0.3	2.91	0.35	17500	1.2
**BFS**	33.7	9.2	0.7	38.6	9.9	0.6	0.7	1.2	3.2	2.93	0.55	27500	2.4

*Calculated as in Durdziński et al. Fly ash as an assemblage of model Ca–Mg–Na-aluminosilicate glasses, Cement and Concrete Research, 2015.

Raw material characterization:

The chemical compositions of the raw materials were determined using a 4 kV wavelength dispersive x-ray fluorescence (XRF) spectrometer (PANalytical AxiosmAX). XRF analyses were performed from melt fused samples using x-ray flux type 66:34 (66 wt% $Li_2B_4O_7$ and 34 wt% $LiBO_2$), and x-ray diffraction (XRD) analysis was performed with a Rigaku Smartlab diffractometer (9kW Cu x-ray source) in the range of 5-70° 2θ using a 5°2θ/min scan speed.

The densities of the raw materials were measured with a helium pycnometer (Micrometrics, USA). The specific surface area measurement was based on the physical adsorption of N_2 gas molecules on a solid surface using a physisorption analyzer (ASAP 2020, Micrometrics), and the results were reported using BET relationship. Approximately 1 g of raw material was degassed in a vacuum (< 300 µmHg) for 2 h at 300 °C. The surface area was determined at liquid nitrogen temperature (77 K) and in the relative pressure range of $0.05 < P/P_0 < 0.3$. As the surface areas of the raw materials are small, the uncertainty of the specific surface area method used here is in the order of ±0.05 m^2/g.

Table S2. log K values used in PHREEQC. The data is organized as in the PCHatches.dat-file. For full database list please see https://www.usgs.gov/software/phreeqc-version-3

```
H+ + H2Tartrate-2 = H(H2Tartrate)-
        log_k        3.97
        delta_h      -1.6
```

    #   logK source: NIST46, version 8. T and ionic strength: 25 and 0.1
    #   delta_h source: NIST46, version 8. T and ionic strength: 25 and 0.1

H+ + H(H2Tartrate)- = H2(H2Tartrate)
    log_k   2.82
    delta_h   -3.4
    #   logK source: NIST46, version 8. T and ionic strength: 25 and 0.1
    #   delta_h source: NIST46, version 8. T and ionic strength: 25 and 0.1

H2Tartrate-2 = HTartrate-3 + H+
    log_k   -12.43
    #   log_k source: pKa3 from https://drugcentral.org/drugcard/2566?q=tartaric%20acid

HTartrate-3 = Tartrate-4 + H+
    log_k   -12.97
    #log_k source: pKa4 from https://drugcentral.org/drugcard/2566?q=tartaric%20acid

Na+ + H2Tartrate-2 = Na(H2Tartrate)-
    log_k   0.5
    delta_h   -0.8
    #   logK source: NIST46, version 8. T and ionic strength: 25 and 0.1
    #   delta_h source: NIST46, version 8. T and ionic strength: 25 and 0.1

Na+ + H(H2Tartrate)- = NaH(H2Tartrate)
    log_k   0
    delta_h   -2
    #   logK source: NIST46, version 8. T and ionic strength: 25 and 0.1
    #   delta_h source: NIST46, version 8. T and ionic strength: 25 and 0.1

Ca+2 + H2Tartrate-2 = Ca(H2Tartrate)
    log_k   1.86
    delta_h   -8
    #   logK source: NIST46, version 8. T and ionic strength: 25 and 0.1
    #   delta_h source: NIST46, version 8. T and ionic strength: 25 and 0

Ca+2 + H(H2Tartrate)- = CaH(H2Tartrate)+
    log_k   1.2
    delta_h   -8
    #   logK source: NIST46, version 8. T and ionic strength: 25 and 0.1
    #   delta_h source: NIST46, version 8. T and ionic strength: 25 and 0

Mg+2 + H2Tartrate-2 = Mg(H2Tartrate)
    log_k   1.44
    #   logK source: NIST46, version 8. T and ionic strength: 25 and 0.1

Mg+2 + H(H2Tartrate)- = MgH(H2Tartrate)+
log_k 0.95
logK source: NIST46, version 8. T and ionic strength: 25 and 0.1

Al+3 + H2Tartrate-2 = Al(H2Tartrate)+
log_k 2.96
logK source: NIST46, version 8. T and ionic strength: 25 and 0.5

Al+3 + H2Tartrate-2 = Al(HTartrate) + H+
log_k 1.18
logK source: NIST46, version 8. T and ionic strength: 25 and 0.1

Al+3 + 3H2Tartrate-2 = Al(Tartrate)(HTartrate)2-7 + 4H+
log_k -15.92
logK source: NIST46, version 8. T and ionic strength: 25 and 0.1

2Al+3 + 2H2Tartrate-2 = Al2(HTartrate)2 + 2H+
log_k 3.89
logK source: NIST46, version 8. T and ionic strength: 25 and 0.5

Al2(HTartrate)2 + H+ = Al2(HTartrate)(H2Tartrate)+
log_k 2.61
logK source: NIST46, version 8. T and ionic strength: 25 and 0.5

Al(HTartrate) = Al(Tartrate)- + H+
log_k -5.15
logK source: NIST46, version 8. T and ionic strength: 25 and 0.1

Al+3 + 2H2Tartrate-2 = Al(H2Tartrate)2-
log_k 7.65
logK source: NIST46, version 8. T and ionic strength: 25 and 0.1

Al(H2Tartrate)2- = Al(HTartrate)(H2Tartrate)-2 + H+
log_k -3.72
logK source: NIST46, version 8. T and ionic strength: 25 and 0.1

Al(HTartrate)(H2Tartrate)-2 = Al(Tartrate)(HTartrate)-4 + 2H+
log_k -12.67
logK source: NIST46, version 8. T and ionic strength: 25 and 0.1

Al(Tartrate)(HTartrate)2-7 = Al(Tartrate)2(HTartrate)-8 + H+
log_k -10.89
logK source: NIST46, version 8. T and ionic strength: 25 and 0.1

Al(Tartrate)2(HTartrate)-8 = Al(Tartrate)3-9 + H+
log_k -12.7
logK source: NIST46, version 8. T and ionic strength: 25 and 0.1

Al2(HTartrate)2 = Al2(HTartrate)(Tartrate)- + H+
log_k -4.21
logK source: NIST46, version 8. T and ionic strength: 25 and 0.5

Al2(HTartrate)(Tartrate)- = Al2(Tartrate)2-2 + H+
log_k -5.83
logK source: NIST46, version 8. T and ionic strength: 25 and 0.5

Fe+2 + H2Tartrate-2 = Fe(H2Tartrate)
log_k 2.24
logK source: NIST46, version 8. T and ionic strength: 20 and 0.1

Fe+3 + H2Tartrate-2 = Fe(H2Tartrate)+
log_k 6.49
logK source: NIST46, version 8. T and ionic strength: 20 and 0.1

2Fe+3 + 2H2Tartrate-2 = Fe2(HTartrate)2 + 2H+
log_k 11.87
logK source: NIST46, version 8. T and ionic strength: 20 and 0.1. DL-mixture

3Fe+3 + 3H2Tartrate-2 = Fe3(Tartrate)3-3 + 6H+
log_k 9.5
logK source: NIST46, version 8. T and ionic strength: 20 and 0.1

Fe2(HTartrate)2 = Fe2(HTartrate)(Tartrate)- + H+
log_k -2.8
logK source: NIST46, version 8. T and ionic strength: 20 and 0.1. DL-mixture

Fe2(HTartrate)2 = Fe2(Tartrate)2-2 + 2H+
log_k -4.9
logK source: NIST46, version 8. T and ionic strength: 25 and 1

H+ + HCitrate-3 = H(HCitrate)-2
log_k 5.64
delta_h 1.9
logK source: NIST46, version 8. T and ionic strength: 25 and 0.1
delta_h source: NIST46, version 8. T and ionic strength: 25 and 0.1

2H+ + HCitrate-3 = H2(HCitrate)-

log_k 4.35
delta_h -3.1
logK source: NIST46, version 8. T and ionic strength: 25 and 0.1
delta_h source: NIST46, version 8. T and ionic strength: 25 and 0.1

3H+ + HCitrate-3 = H3(HCitrate)
log_k 2.9
delta_h -4.6
logK source: NIST46, version 8. T and ionic strength: 25 and 0.1
delta_h source: NIST46, version 8. T and ionic strength: 25 and 0.1

Na+ + HCitrate-3 = Na(HCitrate)-2
log_k 0.75
delta_h 1
logK source: NIST46, version 8. T and ionic strength: 25 and 0.1
delta_h source: NIST46, version 8. T and ionic strength: 25 and 0.1

Fe+2 + HCitrate-3 = Fe(HCitrate)-
log_k 4.4
logK source: NIST46, version 8. T and ionic strength: 25 and 0.1

Fe+2 + H(HCitrate)-2 = FeH(HCitrate)
log_k 2.9
logK source: NIST46, version 8. T and ionic strength: 25 and 0.1

Fe+2 + H2(HCitrate)- = FeH2(HCitrate)+
log_k 1.1
logK source: NIST46, version 8. T and ionic strength: 37 and 0.15

FeH(HCitrate) + HCitrate-3 = FeH(HCitrate)2-3
log_k 3.19
logK source: NIST46, version 8. T and ionic strength: 37 and 0.15
Fe+2

2Fe+2 + 2HCitrate-3 = Fe2(Citrate)2-4 + 2H+
log_k -5.4
logK source: NIST46, version 8. T and ionic strength: 37 and 0.15

Fe+3 + HCitrate-3 = Fe(HCitrate)
log_k 11.19
delta_h -28
logK source: NIST46, version 8. T and ionic strength: 25 and 0.1
delta_h source: NIST46, version 8. T and ionic strength: 25 and 0

Fe+3 + H(HCitrate)-2 = FeH(HCitrate)+
log_k 6.7
logK source: NIST46, version 8. T and ionic strength: 25 and 0.1

Fe(HCitrate) + H2O = FeOH(HCitrate)- + H+
log_k -2.7
logK source: NIST46, version 8. T and ionic strength: 25 and 0.1

2Fe+3 + 2HCitrate-3 + 2H2O = Fe2(OH)2(HCitrate)2-2 + 2H+
log_k 21.2
logK source: NIST46, version 8. T and ionic strength: 20 and 0.1

Ca+2 + HCitrate-3 = Ca(HCitrate)-
log_k 3.48
delta_h 1.2
logK source: NIST46, version 8. T and ionic strength: 25 and 0.1
delta_h source: NIST46, version 8. T and ionic strength: 25 and 0.1

Ca+2 + H(HCitrate)-2 = CaH(HCitrate)
log_k 2.07
logK source: NIST46, version 8. T and ionic strength: 25 and 0.1

Ca+2 + H2(HCitrate)- = CaH2(HCitrate)+
log_k 1
logK source: NIST46, version 8. T and ionic strength: 25 and 0.1

Mg+2 + HCitrate-3 = Mg(HCitrate)-
log_k 3.43
logK source: NIST46, version 8. T and ionic strength: 25 and 0.1

Mg+2 + H(HCitrate)-2 = MgH(HCitrate)
log_k 1.8
logK source: NIST46, version 8. T and ionic strength: 25 and 0.1

Mg+2 + H2(HCitrate)- = MgH2(HCitrate)+
log_k 0.7
logK source: NIST46, version 8. T and ionic strength: 25 and 0.1

Al+3 + HCitrate-3 = Al(HCitrate)
log_k 8.35
logK source: NIST46, version 8. T and ionic strength: 25 and 0.1

Al+3 + 2HCitrate-3 = Al(HCitrate)2-3
log_k 13.4

#	logK source: NIST46, version 8. T and ionic strength: 25 and 0.1

Al+3 + H(HCitrate)-2 = AlH(HCitrate)+
	log_k	5.31
	#	logK source: NIST46, version 8. T and ionic strength: 25 and 0.1

Al+3 + HCitrate-3 = Al(Citrate)- + H+
	log_k	4.67
	delta_h	41
	#	logK source: NIST46, version 8. T and ionic strength: 25 and 0.1
	#	delta_h source: NIST46, version 8. T and ionic strength: 25 and 0

Al(Citrate)- + H2O = Al(OH)(Citrate)-2 + H+
	log_k	-6.2
	#	logK source: NIST46, version 8. T and ionic strength: 25 and 0.5

Al(HCitrate)2-3 = Al(Citrate)(HCitrate)-4 + H+
	log_k	-6.1
	#	logK source: NIST46, version 8. T and ionic strength: 25 and 0.1

Al(HCitrate)2-3 = Al(Citrate)2-5 + 2H+
	log_k	-13.27
	#	logK source: NIST46, version 8. T and ionic strength: 25 and 0.1

2Al+3 + 2HCitrate-3 + 2H2O = Al2(OH)2(HCitrate)2-2 + 2H+
	log_k	12.69
	#	logK source: NIST46, version 8. T and ionic strength: 37 and 0.15

3Al+3 + 3HCitrate-3 + 4H2O = Al3(OH)4(HCitrate)3-4 + 4H+
	log_k	14.83
	#	logK source: NIST46, version 8. T and ionic strength: 25 and 0.2

3Al+3 + 3HCitrate-3 + 4H2O = Al3(OH)4(Citrate)3-7 + 7H+
	log_k	-10.91
	#	logK source: NIST46, version 8. T and ionic strength: 25 and 0.5

H4SiO4 + HCitrate-3 = H4SiO4(HCitrate)-3
	log_k	0.11
	#	logK source: NIST46, version 8. T and ionic strength: 25 and 0.5

Al+3 + H2O = AlOH+2 + H+
	log_k -4.9
	delta_h 13.2 kcal
Al+3 + 2H2O = Al(OH)2+1 + 2H+

```
        log_k -10.1
        delta_h  26.9 kcal
 Al+3 + 3H2O = Al(OH)3 + 3H+
        log_k -16.9
        delta_h  39.89 kcal
 Al+3 + 4H2O = Al(OH)4-1 + 4H+
        log_k -22.87
        delta_h  43.3 kcal

 Ca+2 + H2O = Ca(OH)+1 + H+
        log_k -12.6
        delta_h  14.535 kcal

 Fe+2 + H2O = Fe(OH)+1 + H+
        log_k -9.5
        delta_h  13.2 kcal
 Fe+2 + 2H2O = Fe(OH)2 + 2H+
        log_k -20.6
        delta_h  28.6 kcal
 Fe+2 + 3H2O = Fe(OH)3-1 + 3H+
        log_k -31
        delta_h  30.3 kcal
 Fe+2 + 4H2O = Fe(OH)4-2 + 4H+
        log_k -46
        delta_h  0 kcal

 3H4SiO4 = H3Si3O9-3 + 3H+ + 3H2O
        log_k -28.6
        delta_h 0 kcal
 H4SiO4 = H3SiO4-1 + H+
        log_k -9.81
        delta_h  6.12 kcal

 2H4SiO4 = H4Si2O7-2 + 2H+ + H2O
        log_k -19
        delta_h 0 kcal
 4H4SiO4 = H4Si4O12-4 + 4H+ + 4H2O
        log_k -36.3
        delta_h 0 kcal
 2H4SiO4 = H5Si2O7-1 + H+ + H2O
        log_k -8.1
        delta_h 0 kcal
 3H4SiO4 = H5Si3O10-3 + 3H+ + 2H2O
        log_k -27.5
```

delta_h 0 kcal
4H4SiO4 = H5Si4O12-3 + 3H+ + 4H2O
log_k -25.5
delta_h 0 kcal

H2O + Mg+2 = MgOH+1 + H+
log_k -11.79
delta_h 15.419 kcal
PHASES:
Ca3(HCitrate)2:4H2O
Ca3(HCitrate)2:4H2O = 3Ca+2 + 2HCitrate-3 + 4H2O

log_k -11.84
logK source: # Calculated based on solubility in Table 6.12 Performance of Cement - based Materials in Aggressive Aqueous Environments, RILEM State - of - the - Art Report TC 211-PAE (Alexander M, De Belie N, Bertron A (eds)). Springer, Dordrecht, Netherlands, pp. 145.
CaH(HCitrate)
CaH(HCitrate) = Ca+2 + H+ + HCitrate-3
log_k -11.39
delta_h 0 kcal
data from NIST46, version 8. T=25C

Ca3(HCitrate)2
Ca3(HCitrate)2 = 3Ca+2 + 2HCitrate-3
log_k -17.03
delta_h 0 kcal
#data from NIST46, version 8. T=25C

Ca(H2Tartrate):4H2O
Ca(H2Tartrate):4H2O = Ca+2 + H2Tartrate-2 + 4H2O
log_k -5.98
logK source: Calculated based on solubility in Bertron A and Duchesne J (2013) Attack of cementitious materials by organic acids in agricultural and agrofood effluents. In Performance of Cement - based Materials in Aggressive Aqueous Environments, RILEM State - of - the - Art Report TC 211-PAE (Alexander M, De Belie N, Bertron A (eds)). Springer, Dordrecht, Netherlands, pp. 131-173.
Mg(H2Tartrate):5H2O
Mg(H2Tartrate):5H2O = Mg+2 + H2Tartrate-2 + 5H2O
log_k -3.03
logK source: Calculated based on solubility in Bertron A and Duchesne J (2013) Attack of cementitious materials by organic acids in agricultural and agrofood effluents. In Performance of Cement - based Materials in Aggressive Aqueous Environments, RILEM State - of - the - Art Report TC 211-PAE (Alexander M, De Belie N, Bertron A (eds)). Springer, Dordrecht, Netherlands, pp. 131-173.
Fe(H2Tartrate):3H2O
Fe(H2Tartrate):3H2O = Fe+2 + H2Tartrate-2 + 3H2O
log_k -8.23
#logK source: Cayot P, Guzun-Cojocaru T and Cayot N (2013) Iron fortification of milk and dairy products. In Handbook of Food Fortification and Health: From Concepts to Public Health Applications (Preedy VR, Srirajaskanthan R and Patel VB (eds)). Springer, New York, 2013, vol. 1, pp. 75-89.

Table S3. Molar ratios in solution calculated based on ICP-MS data.

	BG_XRF*	BG_i	BG_8	BG_CA_8	BG_TA_8	BG_6	BG_CA_6	BG_TA_6
Al/Si	0.44	0.35	0.12	0.45	0.15	0.06	0.68	0.48
Fe/Si	0.07	0.02	0.06	0.37	0.18	0.24	0.44	0.60
Ca/Si	0.57	2.34	3.11	2.16	3.03	2.75	1.52	2.31
Mg/Si	0.33	1.28	1.77	1.25	1.72	1.51	0.90	1.27
	BFS_XRF*	BFS_i	BFS_8	BFS_CA_8	BFS_TA_8	BFS_6	BFS_CA_6	BFS_TA_6
Al/Si	0.32	n.d.	n.d.	0.07	0.05	0.05	0.24	0.21
Fe/Si	0.02	n.d.	n.d.	0.03	0.03	0.02	0.09	0.03
Ca/Si	1.23	4.11	14.02	8.76	4.00	18.12	17.41	1.75
Mg/Si	0.44	0.19	3.95	2.60	2.31	5.98	5.60	3.27

*element ratios of the bulk glass. Calculated based on XRF data.

n.d. under detection limit

Table S4. XPS analysis results as atomic-% and C1s spectra functional groups and fittings for BFS_TA_8.

Name	BG	BG_i	BG_8	BG_CA_8	BG_TA_8	BG_6	BG_CA_6	BG_TA_6
Al2p	7.18	7.38	7.67	8.04	7.28	8.05	7.38	7.72
C1s	8.78	10.84	11.09	8.94	12.01	11.11	9.96	10.74
Ca2p	6.94	5.99	5.37	5.54	5.54	4.71	3.82	4.60
Cl2p	0.12	0.05	0.04	0.08	0.06	0.07	0.05	0.10
Fe2p	1.24	1.31	1.93	1.70	2.15	1.83	1.98	2.14
K2p	0.46	0.21	0.14	0.21	0.24	0.14	0.14	0.11
Mg1s	4.72	3.35	2.21	2.77	2.17	1.89	1.53	1.76
Na1s	0.26	0.01	0.01	0.01	0.05	0.07	0.01	0.04
O1s	54.80	54.36	55.67	55.42	54.31	55.54	57.26	55.74
P2p	0.21	0.07	0.12	0.23	0.15	0.14	0.17	0.13
S2p	0.29	0.06	0.00	0.02	0.03	0.00	0.01	0.03
Si2p	14.45	15.70	15.12	16.23	15.32	15.70	17.07	16.15
Ti2p	0.54	0.68	0.63	0.80	0.68	0.74	0.63	0.74
	BFS	BFS_i	BFS_8	BFS_CA_8	BFS_TA_8	BFS_6	BFS_CA_6	BFS_TA_6
Al2p	4.69	4.57	6.17	5.74	6.09	7.27	5.99	n/a
C1s	8.22	10.01	7.68	9.02	10.39	6.37	7.06	n/a
Ca2p	6.70	6.82	3.41	4.33	3.98	2.82	2.68	n/a
Cl2p	0.06	0.16	0.03	0.03	0.03	0.03	0.04	n/a
Fe2p	0.23	0.24	0.46	0.53	0.55	0.56	0.35	n/a

K2p	0.31	0.19	0.14	0.13	0.10	0.15	0.26	n/a
Mg1s	8.75	7.91	2.68	3.44	3.35	1.95	2.23	n/a
Na1s	0.63	0.36	0.02	0.08	0.01	0.02	0.08	n/a
O1s	55.65	55.18	59.72	58.35	57.89	61.04	60.46	n/a
P2p	0.09	0.16	0.03	0.05	0.06	0.14	0.05	n/a
S2p	0.49	0.42	0.15	0.11	0.06	0.03	0.09	n/a
Si2p	13.79	13.58	18.94	17.71	17.05	19.10	20.23	n/a
Ti2p	0.39	0.41	0.58	0.48	0.44	0.54	0.49	n/a
	BG	BG_i	BG_8	BG_CA_8	BG_TA_8	BG_6	BG_CA_6	BG_TA_6
Al/Si	0.50	0.47	0.51	0.50	0.48	0.51	0.43	0.48
Fe/Si	0.09	0.08	0.13	0.10	0.14	0.12	0.12	0.13
Ca/Si	0.48	0.38	0.36	0.34	0.36	0.30	0.22	0.28
Mg/Si	0.33	0.21	0.15	0.17	0.14	0.12	0.09	0.11
Ti/Si	0.04	0.04	0.04	0.05	0.04	0.05	0.04	0.05
	BFS	BFS_i	BFS_8	BFS_CA_8	BFS_TA_8	BFS_6	BFS_CA_6	BFS_TA_6
Al/Si	0.34	0.34	0.33	0.32	0.36	0.38	0.30	n/a
Fe/Si	0.02	0.02	0.02	0.03	0.03	0.03	0.02	n/a
Ca/Si	0.49	0.50	0.18	0.24	0.23	0.15	0.13	n/a
Mg/Si	0.63	0.58	0.14	0.19	0.20	0.10	0.11	n/a
Ti/Si	0.03	0.03	0.03	0.03	0.03	0.03	0.02	n/a

Table S4b. Functional groups in C1s spectra

	C1s				
eV	284.8	285.6	286.6	288-289	290
Corresponding functional group	C-C	CH_2	C-OH	O-C=O, C=O	CO_3, COO^-

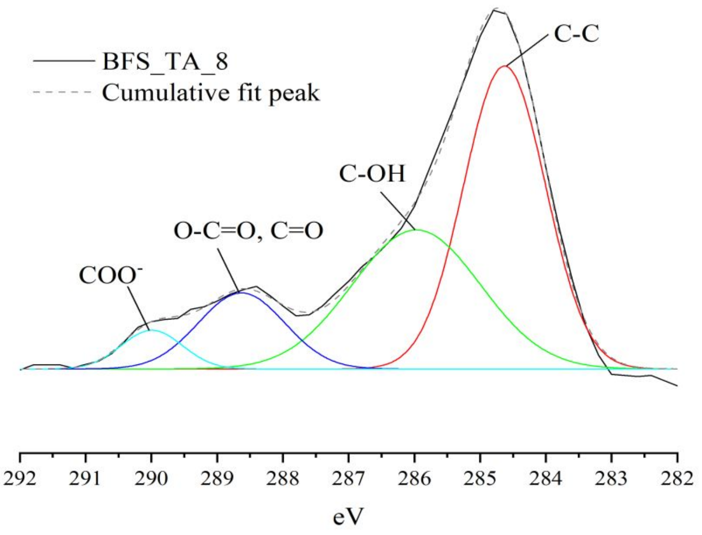

Figure S4b. Fitting of C1s for BFS_TA_8.

Table S5. Distribution of aqueous species and saturation indexes of solid phases as modelled with PHREEQC-software. Results organized as in PHREEQC. pH was set as charged in the modelling.

Sample name			BG_i					
temp		22						
pH		10						
units	mg/kgw							
density		0.997						
Si		2.439231	mg/kgw					
Al		46.20951	umol/kgw					
Fe(2)		7.68536	umol/kgw					
Ca		8.130769	mg/kgw					
Mg		2.710256	mg/kgw					
Ti(4)		3.46432	umol/kgw					
Citrate		0	mol/kgw					
water		1	kg					
Elements	Molality							
Al		4.62E-05						
Ca		2.03E-04						
Fe(2)		7.69E-06						
Mg		1.12E-04						
Si		4.06E-05						
Ti(4)		3.46E-06						
Distribution of species								
					log	log	log	mole V
	Species		Molality	Activity	Molality	Activity	Gamma	cm³/mol
	OH-		8.20E-05	7.95E-05	-4.086	-4.099	-0.013	0
	H+		1.03E-10	1.00E-10	-9.987	-10	-0.013	0
	H2O		5.55E+01	1.00E+00	1.744	0	0	18.06
Al		4.62E-05						
	Al(OH)4-		4.62E-05	4.48E-05	-4.335	-4.348	-0.013	0
	Al(OH)3		4.44E-09	4.44E-09	-8.353	-8.353	0	0
	Al(OH)2+		3.61E-12	3.50E-12	-11.443	-11.456	-0.013	0
	AlOH+2		7.91E-17	7.01E-17	-16.102	-16.154	-0.052	0
	Al+3		9.16E-22	6.99E-22	-21.038	-21.156	-0.118	0

Ca		2.03E-04					
	Ca+2		2.03E-04	1.80E-04	-3.694	-3.746	-0.052	0
	Ca(OH)+		3.62E-07	3.52E-07	-6.441	-6.454	-0.013	0
Fe(2)		7.69E-06						
	Fe(OH)+		5.07E-06	4.92E-06	-5.295	-5.308	-0.013	0
	Fe+2		2.20E-06	1.95E-06	-5.658	-5.71	-0.052	0
	Fe(OH)2		3.00E-07	3.00E-07	-6.523	-6.523	0	0
	Fe(OH)3-		1.20E-07	1.16E-07	-6.923	-6.936	-0.013	0
	Fe(OH)4-2		2.20E-12	1.95E-12	-11.658	-11.71	-0.052	0
H(0)		1.46E-31						
	H2		7.30E-32	7.30E-32	-31.137	-31.137	0	0
Mg		1.12E-04						
	Mg+2		1.10E-04	9.78E-05	-3.958	-4.01	-0.052	0
	MgOH+		1.25E-06	1.22E-06	-5.902	-5.915	-0.013	0
O(0)		1.65E-31						
	O2		8.24E-32	8.24E-32	-31.084	-31.084	0	0
Si		4.06E-05						
	H3SiO4-		2.39E-05	2.32E-05	-4.622	-4.635	-0.013	0
	H4SiO4		1.66E-05	1.66E-05	-4.779	-4.779	0	0
	H5Si2O7-		2.27E-08	2.20E-08	-7.645	-7.658	-0.013	0
	H2SiO4-2		9.99E-09	8.86E-09	-8	-8.053	-0.052	0
	H4Si2O7-2		3.12E-09	2.77E-09	-8.506	-8.558	-0.052	0
	H5Si3O10-3		1.91E-12	1.46E-12	-11.72	-11.837	-0.118	0
	H3Si3O9-3		1.52E-13	1.16E-13	-12.82	-12.937	-0.118	0
	H5Si4O12-3		3.17E-15	2.42E-15	-14.499	-14.616	-0.118	0
	H4Si4O12-4		6.21E-16	3.84E-16	-15.207	-15.416	-0.209	0
Ti(4)		3.46E-06						
	Ti(OH)4		3.46E-06	3.47E-06	-5.46	-5.46	0	0

Saturation indices

	Phase	SI**	log	IAP	log
	afwillite	-7.89	39.2	47.1	Ca3Si2O4(OH)6
	akermanite	-6.83	38.94	45.77	Ca2MgSi2O7
	Al(OH)3(amorph.)	-2.15	8.84	11	Al(OH)3
	amesite-14a	13.53	44.89	31.36	Mg2Al2SiO5(OH)4
	amrph.silica	-2.06	-4.78	-2.72	SiO2
	andalusite	-4.09	12.91	17	Al2SiO5
	anorthite	-3.26	24.38	27.64	CaAl2(SiO4)2
	anthophyllite	4.77	73.7	68.93	Mg7Si8O22(OH)2
	antigorite	120.52	302.52	182	Mg24Si17O42.5(OH)31
	beidellit-ca	-0.95	5.75	6.7	Ca0.165Al2.33Si3.67O10(OH)2
	beidellit-mg	-0.98	5.71	6.69	Mg0.165Al2.33Si3.67O10(OH)2
	boehmite	-0.97	8.84	9.81	AlO2H
	Ca(OH)2	-6.78	16.25	23.04	Ca(OH)2

ca-al	-7.95	29.16	37.11	CaAl2SiO6
ca-p	1.32	21.52	20.2	CaAl2Si2.6O12.4H6.4
Ca2Al2O13H16	-8.43	50.2	58.63	Ca2Al2O13H16
Ca2Al2SiO15H16	-3.63	45.42	49.05	Ca2Al2SiO15H16
Ca2SiO4	-10.18	27.73	37.91	Ca2SiO4
Ca3Al2O12H12	-12.93	66.45	79.38	Ca3Al2O12H12
Ca3Al2Si0.5O12H10	-9.41	64.06	73.47	Ca3Al2Si0.5O12H10
Ca3SiO5	-30.81	43.98	74.79	Ca3SiO5
Ca4Al2O20H26	-19.59	82.71	102.3	Ca4Al2O20H26
CaO	-16.72	16.25	32.97	CaO
chabazite	1.25	14.83	13.58	CaAl2Si4O12:6H2O
chalcedony	-1.22	-4.78	-3.55	SiO2
chamosite-7a	9.8	41.49	31.69	Fe2Al2SiO5(OH)4
chrysotile	6.4	38.41	32.01	Mg3Si2O5(OH)4
chs(0.8)	-2.81	8.22	11.03	Ca0.8SiO5H4.4
clinochl-14a	14.73	83.3	68.57	Mg5Al2Si3O10(OH)8
clinochl-7a	11.36	83.3	71.94	Mg5Al2Si3O10(OH)8
clinozoisite	0	44.7	44.71	Ca2Al3Si3O12(OH)
cordier.anh	-11.6	43.46	55.07	Mg2Al4Si5O18
cordier.hydr	-9.09	43.46	52.55	Mg2Al4Si5O18:H2O
corundum	-4.15	17.69	21.84	Al2O3
crist.beta_amorph	-1.76	-4.78	-3.02	SiO2
cristobalite	-1.31	-4.78	-3.47	SiO2
csh(1.1)	-3.56	13.1	16.66	Ca1.1SiO7H7.8
csh(1.8)	-7.93	24.48	32.41	Ca1.8SiO9H10.4
daphnite-14a	23.49	74.8	51.31	Fe5Al2Si3O10(OH)8
daphnite-7a	20.09	74.8	54.71	Fe5Al2Si3O10(OH)8
diaspore	-0.1	8.84	8.95	AlHO2
diopside	1.41	22.69	21.28	CaMgSi2O6
enstatite	-0.44	11.21	11.65	MgSiO3
epistilbite	-0.24	5.27	5.5	CaAl2Si6O16:5H2O
fayalite	4.48	23.8	19.32	Fe2SiO4
Fe(OH)2	0.28	14.29	14.01	Fe(OH)2
FeO	0.63	14.29	13.66	FeO
ferrosilite	1.98	9.51	7.53	FeSiO3
forsterite	-1.35	27.2	28.55	Mg2SiO4
foshagite	-8.73	50.68	59.41	Ca4Si3O9(OH)2:0.5H2O
gehlenite	-12.11	45.42	57.53	Ca2Al2SiO7
gibbsite	0.92	8.84	7.93	Al(OH)3
greenalite	10.44	33.31	22.87	Fe3Si2O5(OH)4
grossular	-2	52.11	54.11	Ca3Al2(SiO4)3
gyrolite	-2.37	18.17	20.54	Ca2Si3O7(OH)2:1.5H2O
H2(g)	-28	-31.14	-3.14	H2
halloy	-2.35	8.13	10.48	Al2Si2O9H4

hedenbergite	1.15	20.99	19.84	CaFe(SiO3)2
hercynite	4.3	31.98	27.68	FeAl2O4
heulandite-ca	-0.98	0.49	1.47	CaAl2Si7O18:6H2O
hillebrandite	-4.83	27.73	32.55	Ca2SiO3(OH)2:0.167H2O
hydrogarnet	-14.35	66.45	80.8	Ca3Al2O6:6H2O
hydrotalcite	6.31	81.65	75.34	Mg4Al2O17H20
jennite	-28.99	117.61	146.6	Ca9Si6O32H22
kaolinite	-0.97	-38.25	-37.29	Al2Si2O5(OH)4
katoite	-7.22	61.67	68.89	Ca3Al2SiO12H8
kyanite	-3.81	12.91	16.72	Al2SiO5
larnite	-11.69	27.73	39.42	Ca2SiO4
laumontite	-0.58	14.83	15.4	CaAl2Si4O12:4H2O
lawsonite	1.2	24.38	23.19	CaAl2Si2O7(OH)2:H2O
margarite	-1.04	42.07	43.12	CaAl4Si2O10(OH)2
merwinite	-13.89	55.19	69.08	MgCa3(SiO4)2
Mg(OH)2	-1.06	15.99	17.05	Mg(OH)2
minnesotaite	9.61	23.75	14.15	Fe3Si4O10(OH)2
monticellite	-2.67	27.47	30.13	CaMgSiO4
montmor-ca	0.26	3.61	3.35	Ca0.165Mg0.33Al1.67Si4O10(OH)2
montmor-mg	0.29	3.57	3.28	Mg0.495Al1.67Si4O10(OH)2
O2(g)	-28.14	-31.08	-2.95	O2
okenite	1.62	6.7	5.07	CaSi2O4(OH)2:H2O
phillipsite-ca	0.45	10.05	9.6	CaAl2Si5O14:5H2O
prehnite	1.95	35.86	33.91	Ca2Al2Si3O10(OH)2
pseudo	-2.72	11.48	14.2	CaSiO3
pyrophyllite	-2.78	-1.43	1.35	Al2Si4O10(OH)2
quartz	-0.73	-4.78	-4.05	SiO2
rankinite	-13.37	39.2	52.57	Ca3Si2O7
ripidolit-14a	19.65	79.9	60.25	Mg3Fe2Al2Si3O10(OH)8
ripidolit-7a	16.26	79.9	63.64	Mg3Fe2Al2Si3O10(OH)8
saponite-ca	8.72	36.03	27.32	Ca0.165Mg3Al0.33Si3.67O10(OH)2
saponite-h	7.02	33.35	26.33	H0.33Mg3Al0.33Si3.67O10(OH)2
saponite-mg	8.69	35.99	27.3	Mg3.165Al0.33Si3.67O10(OH)2
scolecite	4.47	19.61	15.14	CaAl2Si3O10:3H2O
sepiolite	1.52	-38.75	-40.28	Mg2Si3O7.5OH:3H2O
sillimantite	-4.45	12.91	17.36	Al2SiO5
spinel	-5.4	33.68	39.07	Al2MgO4
stratlingite	-3.91	45.42	49.33	Ca2Al2Si1O15H16
talc	6.89	28.85	21.97	Mg3Si4O10(OH)2
tobermorite(11A)	-14.23	52.6	66.83	Ca5Si6H11O22.5
tobermorite(14A)	-11.72	52.6	64.32	Ca5Si6H21O27.5
tobermorite(9A)	-17.19	52.6	69.79	Ca5Si6H6O20
tremolite	11.55	74.23	62.67	Ca2Mg5Si8O22(OH)2
wairakite	-5.09	14.83	19.92	CaAl2Si4O12:2H2O

wollastonite	-2.31	11.48	13.79	CaSiO3
xonotlite	1.4	68.85	67.45	Ca6Si6O17(OH)2
yugawaralite	-1.55	2.63	4.18	Ca0.5AlSi3O8:2H2O
zoisite	-0.13	44.7	44.84	Ca2Al3(SiO4)3OH

BG_8

temp	22		
pH	8		
pe	4		
density	0.997		
Si	3.109635		
Al	58.90983	umol/kgw	
Fe(2)	9.79763	umol/kgw	
Ca	13.8206		
Mg	4.750831		
Ti(4)	4.41646	umol/kgw	
Citrate	0	mol/kgw	
water	1	#	kg
Elements	Molality		
Al	5.89E-05		
Ca	3.45E-04		
Fe(2)	9.80E-06		
Mg	1.96E-04		
Si	5.18E-05		
Ti(4)	4.42E-06		

Distribution of species

					log	log	log	mole V
	Species		Molality	Activity	Molality	Activity	Gamma	cm³/mol
	OH-		8.26E-07	7.95E-07	-6.083	-6.099	-0.016	0
	H+		1.04E-08	1.00E-08	-7.984	-8	-0.016	0
	H2O		5.55E+01	1.00E+00	1.744	0	0	18.06
Al		5.89E-05						
	Al(OH)4-		5.83E-05	5.62E-05	-4.234	-4.251	-0.016	0
	Al(OH)3		5.56E-07	5.56E-07	-6.255	-6.255	0	0
	Al(OH)2+		4.55E-08	4.38E-08	-7.342	-7.358	-0.016	0
	AlOH+2		1.02E-10	8.78E-11	-9.991	-10.056	-0.065	0
	Al+3		1.23E-13	8.75E-14	-12.911	-13.058	-0.147	0
Ca		3.45E-04						
	Ca+2		3.45E-04	2.97E-04	-3.462	-3.528	-0.065	0
	Ca(OH)+		6.03E-09	5.81E-09	-8.22	-8.236	-0.016	0
Fe(2)		9.80E-06						
	Fe+2		9.58E-06	8.24E-06	-5.019	-5.084	-0.065	0
	Fe(OH)+		2.16E-07	2.08E-07	-6.666	-6.682	-0.016	0

	Fe(OH)2		1.27E-10	1.27E-10	-9.897	-9.897	0	0
	Fe(OH)3-		5.09E-13	4.90E-13	-12.293	-12.31	-0.016	0
	Fe(OH)4-2		9.58E-20	8.24E-20	-19.019	-19.084	-0.065	0
H(0)		1.46E-27						
	H2		7.29E-28	7.30E-28	-27.137	-27.137	0	0
Mg		1.96E-04						
	Mg+2		1.95E-04	1.68E-04	-3.709	-3.774	-0.065	0
	MgOH+		2.17E-08	2.09E-08	-7.663	-7.679	-0.016	0
O(0)		1.65E-39						
	O2		8.24E-40	8.24E-40	-39.084	-39.084	0	0
Si		5.18E-05						
	H4SiO4		5.10E-05	5.10E-05	-4.292	-4.292	0	0
	H3SiO4-		7.39E-07	7.12E-07	-6.131	-6.148	-0.016	0
	H5Si2O7-		2.15E-09	2.07E-09	-8.668	-8.684	-0.016	0
	H2SiO4-2		3.16E-12	2.72E-12	-11.5	-11.566	-0.065	0
	H4Si2O7-2		3.03E-12	2.60E-12	-11.519	-11.584	-0.065	0
	H5Si3O10-3		5.89E-17	4.20E-17	-16.23	-16.377	-0.147	0
	H3Si3O9-3		4.68E-18	3.34E-18	-17.33	-17.477	-0.147	0
	H5Si4O12-3		3.01E-19	2.14E-19	-18.522	-18.669	-0.147	0
	H4Si4O12-4		6.20E-22	3.40E-22	-21.208	-21.469	-0.261	0
Ti(4)		4.42E-06						
	Ti(OH)4		4.42E-06	4.42E-06	-5.355	-5.355	0	0

Saturation indices

Phase	SI**	log	IAP	log
afwillite	-18.27	28.83	47.1	Ca3Si2O4(OH)6
akermanite	-17.19	28.59	45.77	Ca2MgSi2O7
Al(OH)3(amorph.)	-0.06	10.94	11	Al(OH)3
amesite-14a	10.69	42.04	31.36	Mg2Al2SiO5(OH)4
amrph.silica	-1.57	-4.29	-2.72	SiO2
andalusite	0.59	17.59	17	Al2SiO5
anorthite	-1.87	25.77	27.64	CaAl2(SiO4)2
anthophyllite	-17.69	51.24	68.93	Mg7Si8O22(OH)2
antigorite	38.45	220.45	182	Mg24Si17O42.5(OH)31
beidellit-ca	5.1	11.8	6.7	Ca0.165Al2.33Si3.67O10(OH)2
beidellit-mg	5.07	11.76	6.69	Mg0.165Al2.33Si3.67O10(OH)2
boehmite	1.13	10.94	9.81	AlO2H
Ca(OH)2	-10.57	12.47	23.04	Ca(OH)2
ca-al	-7.05	30.06	37.11	CaAl2SiO6
ca-p	3	23.2	20.2	CaAl2Si2.6O12.4H6.4
Ca2Al2O13H16	-11.8	46.83	58.63	Ca2Al2O13H16
Ca2Al2SiO15H16	-6.51	42.54	49.05	Ca2Al2SiO15H16

Ca2SiO4	-17.25	20.65	37.91	Ca2SiO4
Ca3Al2O12H12	-20.08	59.3	79.38	Ca3Al2O12H12
Ca3Al2Si0.5O12H10	-16.32	57.15	73.47	Ca3Al2Si0.5O12H10
Ca3SiO5	-41.67	33.12	74.79	Ca3SiO5
Ca4Al2O20H26	-30.53	71.77	102.3	Ca4Al2O20H26
CaO	-20.5	12.47	32.97	CaO
chabazite	3.61	17.19	13.58	CaAl2Si4O12:6H2O
chalcedony	-0.74	-4.29	-3.55	SiO2
chamosite-7a	7.74	39.42	31.69	Fe2Al2SiO5(OH)4
chrysotile	-3.92	28.09	32.01	Mg3Si2O5(OH)4
chs(0.8)	-5.34	5.69	11.03	Ca0.8SiO5H4.4
clinochl-14a	1.57	70.14	68.57	Mg5Al2Si3O10(OH)8
clinochl-7a	-1.8	70.14	71.94	Mg5Al2Si3O10(OH)8
clinozoisite	0.19	44.89	44.71	Ca2Al3Si3O12(OH)
cordier.anh	-8.31	46.76	55.07	Mg2Al4Si5O18
cordier.hydr	-5.8	46.76	52.55	Mg2Al4Si5O18:H2O
corundum	0.05	21.88	21.84	Al2O3
crist.beta_amorph	-1.27	-4.29	-3.02	SiO2
cristobalite	-0.82	-4.29	-3.47	SiO2
csh(1.1)	-7.23	9.43	16.66	Ca1.1SiO7H7.8
csh(1.8)	-14.25	18.16	32.41	Ca1.8SiO9H10.4
daphnite-14a	12.28	63.59	51.31	Fe5Al2Si3O10(OH)8
daphnite-7a	8.88	63.59	54.71	Fe5Al2Si3O10(OH)8
diaspore	2	10.94	8.95	AlHO2
diopside	-5.16	16.11	21.28	CaMgSi2O6
enstatite	-3.72	7.93	11.65	MgSiO3
epistilbite	3.1	8.6	5.5	CaAl2Si6O16:5H2O
fayalite	-1.78	17.54	19.32	Fe2SiO4
Fe(OH)2	-3.09	10.92	14.01	Fe(OH)2
FeO	-2.74	10.92	13.66	FeO
ferrosilite	-0.91	6.62	7.53	FeSiO3
forsterite	-8.39	20.16	28.55	Mg2SiO4
foshagite	-22.4	37.01	59.41	Ca4Si3O9(OH)2:0.5H2O
gehlenite	-14.99	42.54	57.53	Ca2Al2SiO7
gibbsite	3.01	10.94	7.93	Al(OH)3
greenalite	1.29	24.16	22.87	Fe3Si2O5(OH)4
grossular	-7.69	46.42	54.11	Ca3Al2(SiO4)3
gyrolite	-8.47	12.07	20.54	Ca2Si3O7(OH)2:1.5H2O
H2(g)	-24	-27.14	-3.14	H2
halloy	2.82	13.3	10.48	Al2Si2O9H4
hedenbergite	-5.04	14.8	19.84	CaFe(SiO3)2
hercynite	5.12	32.8	27.68	FeAl2O4
heulandite-ca	2.85	4.31	1.47	CaAl2Si7O18:6H2O
hillebrandite	-11.9	20.65	32.55	Ca2SiO3(OH)2:0.167H2O

hydrogarnet	-21.5	59.3	80.8	Ca3Al2O6:6H2O
hydrotalcite	-4.55	70.79	75.34	Mg4Al2O17H20
jennite	-60.1	86.5	146.6	Ca9Si6O32H22
kaolinite	4.2	-33.09	-37.29	Al2Si2O5(OH)4
katoite	-13.88	55.01	68.89	Ca3Al2SiO12H8
kyanite	0.87	17.59	16.72	Al2SiO5
larnite	-18.77	20.65	39.42	Ca2SiO4
laumontite	1.78	17.19	15.4	CaAl2Si4O12:4H2O
lawsonite	2.58	25.77	23.19	CaAl2Si2O7(OH)2:H2O
margarite	4.54	47.66	43.12	CaAl4Si2O10(OH)2
merwinite	-28.03	41.06	69.08	MgCa3(SiO4)2
Mg(OH)2	-4.83	12.23	17.05	Mg(OH)2
minnesotaite	1.43	15.58	14.15	Fe3Si4O10(OH)2
monticellite	-9.73	20.41	30.13	CaMgSiO4
montmor-ca	3.84	7.2	3.35	Ca0.165Mg0.33Al1.67Si4O10(OH)2
montmor-mg	3.88	7.16	3.28	Mg0.495Al1.67Si4O10(OH)2
O2(g)	-36.14	-39.08	-2.95	O2
okenite	-1.18	3.89	5.07	CaSi2O4(OH)2:H2O
phillipsite-ca	3.29	12.9	9.6	CaAl2Si5O14:5H2O
prehnite	0.04	33.95	33.91	Ca2Al2Si3O10(OH)2
pseudo	-6.02	8.18	14.2	CaSiO3
pyrophyllite	3.36	4.72	1.35	Al2Si4O10(OH)2
quartz	-0.25	-4.29	-4.05	SiO2
rankinite	-23.74	28.83	52.57	Ca3Si2O7
ripidolit-14a	7.27	67.52	60.25	Mg3Fe2Al2Si3O10(OH)8
ripidolit-7a	3.87	67.52	63.64	Mg3Fe2Al2Si3O10(OH)8
saponite-ca	-0.72	26.59	27.32	Ca0.165Mg3Al0.33Si3.67O10(OH)2
saponite-h	-1.8	24.54	26.33	H0.33Mg3Al0.33Si3.67O10(OH)2
saponite-mg	-0.75	26.55	27.3	Mg3.165Al0.33Si3.67O10(OH)2
scolecite	6.34	21.48	15.14	CaAl2Si3O10:3H2O
sepiolite	-4.55	-44.82	-40.28	Mg2Si3O7.5OH:3H2O
sillimantite	0.23	17.59	17.36	Al2SiO5
spinel	-4.96	34.11	39.07	Al2MgO4
stratlingite	-6.79	42.54	49.33	Ca2Al2Si1O15H16
talc	-2.46	19.51	21.97	Mg3Si4O10(OH)2
tobermorite(11A)	-30.22	36.61	66.83	Ca5Si6H11O22.5
tobermorite(14A)	-27.71	36.61	64.32	Ca5Si6H21O27.5
tobermorite(9A)	-33.18	36.61	69.79	Ca5Si6H6O20
tremolite	-10.94	51.74	62.67	Ca2Mg5Si8O22(OH)2
wairakite	-2.73	17.19	19.92	CaAl2Si4O12:2H2O
wollastonite	-5.61	8.18	13.79	CaSiO3
xonotlite	-18.37	49.08	67.45	Ca6Si6O17(OH)2
yugawaralite	0.12	4.3	4.18	Ca0.5AlSi3O8:2H2O
zoisite	0.06	44.89	44.84	Ca2Al3(SiO4)3OH

temp	22	
pH	8	
pe	4	
density	0.997	
Si	4.675145	
Al	2.025896	
Fe(2)		
Ca	14.41503	
Mg	5.06474	
Ti(4)	0	
Citrate	0.000388	
water	1	kg
Elements	Molality	
Al	7.51E-05	
Ca	3.60E-04	
Citrate	3.88E-04	
Fe(2)	6.14E-05	
Mg	2.08E-04	
Si	7.78E-05	

Distribution of species

		Species	Molality	Activity	log Molality	log Activity	log Gamma	mole V cm³/mol
		OH-	8.35E-07	7.95E-07	-6.079	-6.099	-0.021	0
		H+	1.05E-08	1.00E-08	-7.979	-8	-0.021	0
		H2O	5.55E+01	1.00E+00	1.744	0	0	18.06
Al	7.51E-05							
		Al(OH)(Citrate)-2	7.13E-05	5.89E-05	-4.147	-4.23	-0.083	0
		Al(OH)4-	1.57E-06	1.49E-06	-5.805	-5.826	-0.021	0
		Al(Citrate)2-5	1.07E-06	3.23E-07	-5.97	-6.491	-0.521	0
		Al(Citrate)-	9.79E-07	9.33E-07	-6.009	-6.03	-0.021	0
		Al(Citrate)(HCitrate)-4	1.03E-07	4.77E-08	-6.988	-7.321	-0.333	0
		Al(OH)3	1.48E-08	1.48E-08	-7.83	-7.83	0	0
		Al2(OH)2(HCitrate)2-2	3.31E-09	2.73E-09	-8.481	-8.564	-0.083	0
		Al3(OH)4(HCitrate)3-4	1.92E-09	8.89E-10	-8.718	-9.051	-0.333	0
		Al(OH)2+	1.22E-09	1.17E-09	-8.912	-8.933	-0.021	0
		Al(HCitrate)2-3	9.25E-10	6.01E-10	-9.034	-9.221	-0.188	0
		Al3(OH)4(Citrate)3-7	1.70E-10	1.62E-11	-9.77	-10.791	-1.021	0
		Al(HCitrate)	5.28E-11	5.28E-11	-10.277	-10.277	0	0
		AlOH+2	2.83E-12	2.34E-12	-11.548	-11.631	-0.083	0
		Al+3	3.59E-15	2.33E-15	-14.445	-14.633	-0.188	0
		AlH(HCitrate)+	2.19E-16	2.09E-16	-15.66	-15.68	-0.021	0
Ca	3.60E-04							

	Ca+2		2.85E-04	2.35E-04	-3.546	-3.629	-0.083	0
	Ca(HCitrate)-		7.51E-05	7.15E-05	-4.125	-4.145	-0.021	0
	CaH(HCitrate)		1.21E-08	1.21E-08	-7.917	-7.917	0	0
	Ca(OH)+		4.82E-09	4.60E-09	-8.317	-8.337	-0.021	0
	CaH2(HCitrate)+		5.66E-19	5.40E-19	-18.247	-18.268	-0.021	0
Citrate		3.88E-04						
	HCitrate-3		1.56E-04	1.01E-04	-3.807	-3.994	-0.188	0
	Ca(HCitrate)-		7.51E-05	7.15E-05	-4.125	-4.145	-0.021	0
	Al(OH)(Citrate)-2		7.13E-05	5.89E-05	-4.147	-4.23	-0.083	0
	Fe(HCitrate)-		4.17E-05	3.97E-05	-4.38	-4.401	-0.021	0
	Mg(HCitrate)-		3.98E-05	3.80E-05	-4.4	-4.421	-0.021	0
	Al(Citrate)2-5		1.07E-06	3.23E-07	-5.97	-6.491	-0.521	0
	Al(Citrate)-		9.79E-07	9.33E-07	-6.009	-6.03	-0.021	0
	H(HCitrate)-2		5.32E-07	4.39E-07	-6.274	-6.358	-0.083	0
	Fe2(Citrate)2-4		2.14E-07	9.94E-08	-6.669	-7.002	-0.333	0
	Al(Citrate)(HCitrate)-4		1.03E-07	4.77E-08	-6.988	-7.321	-0.333	0
	H4SiO4(HCitrate)-3		1.54E-08	1.00E-08	-7.812	-7.999	-0.188	0
	CaH(HCitrate)		1.21E-08	1.21E-08	-7.917	-7.917	0	0
	FeH(HCitrate)		5.44E-09	5.44E-09	-8.265	-8.265	0	0
	MgH(HCitrate)		3.85E-09	3.85E-09	-8.414	-8.414	0	0
	Al2(OH)2(HCitrate)2-2		3.31E-09	2.73E-09	-8.481	-8.564	-0.083	0
	Al3(OH)4(HCitrate)3-4		1.92E-09	8.89E-10	-8.718	-9.051	-0.333	0
	FeH(HCitrate)2-3		1.32E-09	8.54E-10	-8.881	-9.069	-0.188	0
	Al(HCitrate)2-3		9.25E-10	6.01E-10	-9.034	-9.221	-0.188	0
	Al3(OH)4(Citrate)3-7		1.70E-10	1.62E-11	-9.77	-10.791	-1.021	0
	Al(HCitrate)		5.28E-11	5.28E-11	-10.277	-10.277	0	0
	H2(HCitrate)-		2.41E-16	2.30E-16	-15.618	-15.639	-0.021	0
	AlH(HCitrate)+		2.19E-16	2.09E-16	-15.66	-15.68	-0.021	0
	CaH2(HCitrate)+		5.66E-19	5.40E-19	-18.247	-18.268	-0.021	0
	MgH2(HCitrate)+		1.68E-19	1.60E-19	-18.774	-18.795	-0.021	0
	FeH2(HCitrate)+		4.73E-20	4.51E-20	-19.325	-19.346	-0.021	0
	H3(HCitrate)		8.20E-26	8.20E-26	-25.086	-25.086	0	0
Fe(2)		6.14E-05						
	Fe(HCitrate)-		4.17E-05	3.97E-05	-4.38	-4.401	-0.021	0
	Fe+2		1.89E-05	1.56E-05	-4.724	-4.807	-0.083	0
	Fe(OH)+		4.13E-07	3.93E-07	-6.385	-6.405	-0.021	0
	Fe2(Citrate)2-4		2.14E-07	9.94E-08	-6.669	-7.002	-0.333	0
	FeH(HCitrate)		5.44E-09	5.44E-09	-8.265	-8.265	0	0
	FeH(HCitrate)2-3		1.32E-09	8.54E-10	-8.881	-9.069	-0.188	0
	Fe(OH)2		2.40E-10	2.40E-10	-9.62	-9.62	0	0
	Fe(OH)3-		9.73E-13	9.27E-13	-12.012	-12.033	-0.021	0
	Fe(OH)4-2		1.89E-19	1.56E-19	-18.724	-18.807	-0.083	0
	FeH2(HCitrate)+		4.73E-20	4.51E-20	-19.325	-19.346	-0.021	0
H(0)		1.46E-27						

	H2		7.29E-28	7.30E-28	-27.137	-27.137	0	0
Mg		2.08E-04						
	Mg+2		1.69E-04	1.39E-04	-3.773	-3.857	-0.083	0
	Mg(HCitrate)-		3.98E-05	3.80E-05	-4.4	-4.421	-0.021	0
	MgOH+		1.82E-08	1.73E-08	-7.741	-7.762	-0.021	0
	MgH(HCitrate)		3.85E-09	3.85E-09	-8.414	-8.414	0	0
	MgH2(HCitrate)+		1.68E-19	1.60E-19	-18.774	-18.795	-0.021	0
O(0)		1.65E-39						
	O2		8.23E-40	8.24E-40	-39.084	-39.084	0	0
Si		7.78E-05						
	H4SiO4		7.67E-05	7.67E-05	-4.115	-4.115	0	0
	H3SiO4-		1.12E-06	1.07E-06	-5.95	-5.971	-0.021	0
	H4SiO4(HCitrate)-3		1.54E-08	1.00E-08	-7.812	-7.999	-0.188	0
	H5Si2O7-		4.90E-09	4.67E-09	-8.31	-8.33	-0.021	0
	H4Si2O7-2		7.13E-12	5.88E-12	-11.147	-11.23	-0.083	0
	H2SiO4-2		4.95E-12	4.09E-12	-11.305	-11.389	-0.083	0
	H5Si3O10-3		2.20E-16	1.43E-16	-15.658	-15.846	-0.188	0
	H3Si3O9-3		1.75E-17	1.13E-17	-16.758	-16.946	-0.188	0
	H5Si4O12-3		1.69E-18	1.09E-18	-17.773	-17.961	-0.188	0
	H4Si4O12-4		3.74E-21	1.73E-21	-20.428	-20.761	-0.333	0

Saturation indices

Phase	SI**	log	IAP	log
afwillite	-18.22	28.88	47.1	Ca3Si2O4(OH)6
akermanite	-17.12	28.65	45.77	Ca2MgSi2O7
Al(OH)3(amorph.)	-1.63	9.37	11	Al(OH)3
amesite-14a	7.55	38.91	31.36	Mg2Al2SiO5(OH)4
amrph.silica	-1.39	-4.12	-2.72	SiO2
andalusite	-2.38	14.62	17	Al2SiO5
anorthite	-4.77	22.87	27.64	CaAl2(SiO4)2
anthophyllite	-16.85	52.08	68.93	Mg7Si8O22(OH)2
antigorite	39.48	221.48	182	Mg24Si17O42.5(OH)31
beidellit-ca	2.06	8.76	6.7	Ca0.165Al2.33Si3.67O10(OH)2
beidellit-mg	2.04	8.73	6.69	Mg0.165Al2.33Si3.67O10(OH)2
boehmite	-0.45	9.37	9.81	AlO2H
Ca(OH)2	-10.67	12.37	23.04	Ca(OH)2
ca-al	-10.12	26.99	37.11	CaAl2SiO6
ca-p	0.21	20.41	20.2	CaAl2Si2.6O12.4H6.4
Ca2Al2O13H16	-15.15	43.48	58.63	Ca2Al2O13H16
Ca2Al2SiO15H16	-9.69	39.36	49.05	Ca2Al2SiO15H16
Ca2SiO4	-17.28	20.63	37.91	Ca2SiO4
Ca3(HCitrate)2	-1.85	-18.88	-17.03	Ca3(HCitrate)2
Ca3(HCitrate)2:4H2O	-7.04	-18.88	-11.84	Ca3(HCitrate)2:4H2O

Ca3Al2O12H12	-23.53	55.85	79.38	Ca3Al2O12H12
Ca3Al2Si0.5O12H10	-19.68	53.79	73.47	Ca3Al2Si0.5O12H10
Ca3SiO5	-41.8	33	74.79	Ca3SiO5
Ca4Al2O20H26	-34.08	68.22	102.3	Ca4Al2O20H26
CaH(HCitrate)	-4.23	-15.62	-11.39	CaH(HCitrate)
CaO	-20.6	12.37	32.97	CaO
chabazite	1.06	14.64	13.58	CaAl2Si4O12:6H2O
chalcedony	-0.56	-4.12	-3.55	SiO2
chamosite-7a	5.32	37	31.69	Fe2Al2SiO5(OH)4
chrysotile	-3.81	28.2	32.01	Mg3Si2O5(OH)4
chs(0.8)	-5.25	5.78	11.03	Ca0.8SiO5H4.4
clinochl-14a	-1.46	67.11	68.57	Mg5Al2Si3O10(OH)8
clinochl-7a	-4.83	67.11	71.94	Mg5Al2Si3O10(OH)8
clinozoisite	-4.21	40.5	44.71	Ca2Al3Si3O12(OH)
cordier.anh	-13.89	41.18	55.07	Mg2Al4Si5O18
cordier.hydr	-11.38	41.18	52.55	Mg2Al4Si5O18:H2O
corundum	-3.1	18.73	21.84	Al2O3
crist.beta_amorph	-1.1	-4.12	-3.02	SiO2
cristobalite	-0.65	-4.12	-3.47	SiO2
csh(1.1)	-7.17	9.49	16.66	Ca1.1SiO7H7.8
csh(1.8)	-14.26	18.15	32.41	Ca1.8SiO9H10.4
daphnite-14a	11.04	62.35	51.31	Fe5Al2Si3O10(OH)8
daphnite-7a	7.65	62.35	54.71	Fe5Al2Si3O10(OH)8
diaspore	0.42	9.37	8.95	AlHO2
diopside	-4.99	16.28	21.28	CaMgSi2O6
enstatite	-3.62	8.03	11.65	MgSiO3
epistilbite	0.91	6.41	5.5	CaAl2Si6O16:5H2O
fayalite	-1.05	18.27	19.32	Fe2SiO4
Fe(OH)2	-2.82	11.19	14.01	Fe(OH)2
FeO	-2.47	11.19	13.66	FeO
ferrosilite	-0.45	7.08	7.53	FeSiO3
forsterite	-8.38	20.17	28.55	Mg2SiO4
foshagite	-22.27	37.14	59.41	Ca4Si3O9(OH)2:0.5H2O
gehlenite	-18.17	39.36	57.53	Ca2Al2SiO7
gibbsite	1.44	9.37	7.93	Al(OH)3
greenalite	2.47	25.35	22.87	Fe3Si2O5(OH)4
grossular	-10.61	43.5	54.11	Ca3Al2(SiO4)3
gyrolite	-8.14	12.4	20.54	Ca2Si3O7(OH)2:1.5H2O
H2(g)	-24	-27.14	-3.14	H2
halloy	0.02	10.5	10.48	Al2Si2O9H4
hedenbergite	-4.51	15.33	19.84	CaFe(SiO3)2
hercynite	2.25	29.93	27.68	FeAl2O4
heulandite-ca	0.83	2.3	1.47	CaAl2Si7O18:6H2O
hillebrandite	-11.93	20.63	32.55	Ca2SiO3(OH)2:0.167H2O

hydrogarnet	-24.95	55.85	80.8	Ca3Al2O6:6H2O
hydrotalcite	-8.03	67.31	75.34	Mg4Al2O17H20
jennite	-59.95	86.65	146.6	Ca9Si6O32H22
kaolinite	1.4	-35.88	-37.29	Al2Si2O5(OH)4
katoite	-17.16	51.73	68.89	Ca3Al2SiO12H8
kyanite	-2.1	14.62	16.72	Al2SiO5
larnite	-18.79	20.63	39.42	Ca2SiO4
laumontite	-0.76	14.64	15.4	CaAl2Si4O12:4H2O
lawsonite	-0.31	22.87	23.19	CaAl2Si2O7(OH)2:H2O
margarite	-1.51	41.61	43.12	CaAl4Si2O10(OH)2
merwinite	-28.06	41.03	69.08	MgCa3(SiO4)2
Mg(OH)2	-4.91	12.14	17.05	Mg(OH)2
minnesotaite	2.97	17.12	14.15	Fe3Si4O10(OH)2
monticellite	-9.73	20.4	30.13	CaMgSiO4
montmor-ca	1.88	5.23	3.35	Ca0.165Mg0.33Al1.67Si4O10(OH)2
montmor-mg	1.92	5.19	3.28	Mg0.495Al1.67Si4O10(OH)2
O2(g)	-36.14	-39.08	-2.95	O2
okenite	-0.93	4.14	5.07	CaSi2O4(OH)2:H2O
phillipsite-ca	0.93	10.53	9.6	CaAl2Si5O14:5H2O
prehnite	-2.78	31.13	33.91	Ca2Al2Si3O10(OH)2
pseudo	-5.94	8.26	14.2	CaSiO3
pyrophyllite	0.92	2.27	1.35	Al2Si4O10(OH)2
quartz	-0.07	-4.12	-4.05	SiO2
rankinite	-23.69	28.88	52.57	Ca3Si2O7
ripidolit-14a	4.95	65.2	60.25	Mg3Fe2Al2Si3O10(OH)8
ripidolit-7a	1.56	65.2	63.64	Mg3Fe2Al2Si3O10(OH)8
saponite-ca	-0.86	26.46	27.32	Ca0.165Mg3Al0.33Si3.67O10(OH)2
saponite-h	-1.91	24.42	26.33	H0.33Mg3Al0.33Si3.67O10(OH)2
saponite-mg	-0.88	26.42	27.3	Mg3.165Al0.33Si3.67O10(OH)2
scolecite	3.62	18.76	15.14	CaAl2Si3O10:3H2O
sepiolite	-4.18	-44.46	-40.28	Mg2Si3O7.5OH:3H2O
sillimantite	-2.74	14.62	17.36	Al2SiO5
spinel	-8.2	30.88	39.07	Al2MgO4
stratlingite	-9.97	39.36	49.33	Ca2Al2Si1O15H16
talc	-2	19.97	21.97	Mg3Si4O10(OH)2
tobermorite(11A)	-29.67	37.16	66.83	Ca5Si6H11O22.5
tobermorite(14A)	-27.16	37.16	64.32	Ca5Si6H21O27.5
tobermorite(9A)	-32.62	37.16	69.79	Ca5Si6H6O20
tremolite	-10.14	52.54	62.67	Ca2Mg5Si8O22(OH)2
wairakite	-5.27	14.64	19.92	CaAl2Si4O12:2H2O
wollastonite	-5.53	8.26	13.79	CaSiO3
xonotlite	-17.92	49.53	67.45	Ca6Si6O17(OH)2
yugawaralite	-0.98	3.21	4.18	Ca0.5AlSi3O8:2H2O
zoisite	-4.34	40.5	44.84	Ca2Al3(SiO4)3OH

BFS_TA_8								
	temp	22						
	pH	8						
	pe	4						
	density	0.997						
	Si	63.61141						
	Al	723.88	umol/kgw					
	Fe(2)	4.11						
	Ca	362.95						
	Mg	127.222						
	Ti(4)	58.73861	umol/kgw					
	Tartrate	0.0229	mol/kgw					
	water	1	#	kg				
	Elements	Molality						
	Al	7.24E-04						
	Ca	9.06E-03						
	Fe(2)	7.36E-05						
	Mg	5.23E-03						
	Si	1.06E-03						
	Tartrate	2.29E-02						
	Ti(4)	5.87E-05						
Distribution of species								
	Species	Molality	Activity	log Molality	log Activity	log Gamma	mole V cm³/mol	
	OH-	1.00E-06	7.95E-07	-6	-6.1	-0.1	0	
	H+	1.26E-08	1.00E-08	-7.9	-8	-0.1	0	
	H2O	5.55E+01	9.99E-01	1.744	0	0	18.06	
Al 7.24E-04								
	Al(Tartrate)(HTartrate)2-7	6.89E-04	9.02E-09	-3.162	-8.045	-4.883	0	
	Al(Tartrate)2(HTartrate)-8	2.77E-05	1.16E-11	-4.557	-10.935	-6.378	0	
	Al(Tartrate)(HTartrate)-4	7.19E-06	1.83E-07	-5.143	-6.738	-1.594	0	
	Al(Tartrate)3-9	2.74E-08	2.32E-16	-7.563	-15.635	-8.072	0	
	Al(Tartrate)-	1.82E-08	1.44E-08	-7.741	-7.841	-0.1	0	
	Al(OH)4-	1.45E-09	1.16E-09	-8.838	-8.937	-0.1	0	
	Al(HTartrate)(H2Tartrate)-2	2.14E-10	8.56E-11	-9.669	-10.068	-0.399	0	
	Al(HTartrate)	2.00E-11	2.04E-11	-10.699	-10.691	0.008	0	
	Al(OH)3	1.12E-11	1.14E-11	-10.95	-10.942	0.008	0	
	Al(OH)2+	1.14E-12	9.02E-13	-11.945	-12.045	-0.1	0	
	Al2(Tartrate)2-2	3.22E-14	1.28E-14	-13.493	-13.891	-0.399	0	
	Al(H2Tartrate)2-	5.65E-15	4.49E-15	-14.248	-14.348	-0.1	0	

	AlOH+2		4.53E-15	1.81E-15	-14.344	-14.742	-0.399	0
	Al2(HTartrate)(Tartrate)-		1.09E-16	8.68E-17	-15.962	-16.061	-0.1	0
	Al(H2Tartrate)+		1.55E-17	1.23E-17	-16.811	-16.911	-0.1	0
	Al+3		1.42E-17	1.80E-18	-16.847	-17.744	-0.897	0
	Al2(HTartrate)2		1.38E-20	1.41E-20	-19.859	-19.851	0.008	0
	Al2(HTartrate)(H2Tartrate)+		7.22E-26	5.74E-26	-25.142	-25.241	-0.1	0
Ca		9.06E-03						
	Ca+2		7.43E-03	2.97E-03	-2.129	-2.528	-0.399	0
	Ca(H2Tartrate)		1.63E-03	1.66E-03	-2.788	-2.78	0.008	0
	Ca(OH)+		7.30E-08	5.80E-08	-7.137	-7.236	-0.1	0
	CaH(H2Tartrate)+		4.29E-08	3.41E-08	-7.368	-7.468	-0.1	0
Fe(2)		7.36E-05						
	Fe+2		4.84E-05	1.93E-05	-4.315	-4.714	-0.399	0
	Fe(H2Tartrate)		2.46E-05	2.51E-05	-4.609	-4.601	0.008	0
	Fe(OH)+		6.13E-07	4.87E-07	-6.213	-6.313	-0.1	0
	Fe(OH)2		2.91E-10	2.97E-10	-9.536	-9.528	0.008	0
	Fe(OH)3-		1.44E-12	1.15E-12	-11.841	-11.94	-0.1	0
	Fe(OH)4-2		4.83E-19	1.93E-19	-18.316	-18.715	-0.399	0
H(0)		1.43E-27						
	H2		7.16E-28	7.30E-28	-27.145	-27.137	0.008	0
Mg		5.23E-03						
	Mg+2		4.84E-03	1.94E-03	-2.315	-2.713	-0.399	0
	Mg(H2Tartrate)		3.91E-04	3.98E-04	-3.408	-3.4	0.008	0
	MgOH+		3.03E-07	2.41E-07	-6.519	-6.619	-0.1	0
	MgH(H2Tartrate)+		1.52E-08	1.21E-08	-7.818	-7.918	-0.1	0
O(0)		1.62E-39						
	O2		8.08E-40	8.23E-40	-39.093	-39.085	0.008	0
Si		1.06E-03						
	H4SiO4		1.04E-03	1.06E-03	-2.984	-2.976	0.008	0
	H3SiO4-		1.85E-05	1.47E-05	-4.732	-4.831	-0.1	0
	H5Si2O7-		1.12E-06	8.88E-07	-5.952	-6.051	-0.1	0
	H4Si2O7-2		2.80E-09	1.12E-09	-8.553	-8.951	-0.399	0
	H2SiO4-2		1.41E-10	5.63E-11	-9.851	-10.249	-0.399	0
	H5Si3O10-3		2.95E-12	3.74E-13	-11.53	-12.427	-0.897	0
	H5Si4O12-3		3.12E-13	3.96E-14	-12.506	-13.402	-0.897	0
	H3Si3O9-3		2.34E-13	2.97E-14	-12.63	-13.527	-0.897	0
	H4Si4O12-4		2.47E-15	6.28E-17	-14.608	-16.202	-1.594	0
Tartrate		2.29E-02						
	H2Tartrate-2		1.87E-02	7.47E-03	-1.728	-2.127	-0.399	0
	Ca(H2Tartrate)		1.63E-03	1.66E-03	-2.788	-2.78	0.008	0
	Al(Tartrate)(HTartrate)2-7		6.89E-04	9.02E-09	-3.162	-8.045	-4.883	0
	Mg(H2Tartrate)		3.91E-04	3.98E-04	-3.408	-3.4	0.008	0
	Al(Tartrate)2(HTartrate)-8		2.77E-05	1.16E-11	-4.557	-10.935	-6.378	0
	Fe(H2Tartrate)		2.46E-05	2.51E-05	-4.609	-4.601	0.008	0

	Al(Tartrate)(HTartrate)-4	7.19E-06	1.83E-07	-5.143	-6.738	-1.594	0
	HTartrate-3	2.19E-06	2.77E-07	-5.66	-6.557	-0.897	0
	H(H2Tartrate)-	8.82E-07	7.01E-07	-6.054	-6.154	-0.1	0
	CaH(H2Tartrate)+	4.29E-08	3.41E-08	-7.368	-7.468	-0.1	0
	Al(Tartrate)3-9	2.74E-08	2.32E-16	-7.563	-15.635	-8.072	0
	Al(Tartrate)-	1.82E-08	1.44E-08	-7.741	-7.841	-0.1	0
	MgH(H2Tartrate)+	1.52E-08	1.21E-08	-7.818	-7.918	-0.1	0
	Al(HTartrate)(H2Tartrate)-2	2.14E-10	8.56E-11	-9.669	-10.068	-0.399	0
	Tartrate-4	1.17E-10	2.97E-12	-9.933	-11.527	-1.594	0
	Al(HTartrate)	2.00E-11	2.04E-11	-10.699	-10.691	0.008	0
	H2(H2Tartrate)	4.61E-12	4.70E-12	-11.336	-11.328	0.008	0
	Al2(Tartrate)2-2	3.22E-14	1.28E-14	-13.493	-13.891	-0.399	0
	Al(H2Tartrate)2-	5.65E-15	4.49E-15	-14.248	-14.348	-0.1	0
	Al2(HTartrate)(Tartrate)-	1.09E-16	8.68E-17	-15.962	-16.061	-0.1	0
	Al(H2Tartrate)+	1.55E-17	1.23E-17	-16.811	-16.911	-0.1	0
	Al2(HTartrate)2	1.38E-20	1.41E-20	-19.859	-19.851	0.008	0
	Al2(HTartrate)(H2Tartrate)+	7.22E-26	5.74E-26	-25.142	-25.241	-0.1	0
Ti(4)	5.87E-05						
	Ti(OH)4	5.87E-05	5.98E-05	-4.231	-4.223	0.008	0

Saturation indices

Phase	SI**	log	IAP	log
afwillite	-12.63	34.46	47.1	Ca3Si2O4(OH)6
akermanite	-11.49	34.28	45.77	Ca2MgSi2O7
Al(OH)3(amorph.)	-4.74	6.26	11	Al(OH)3
amesite-14a	4.75	36.11	31.36	Mg2Al2SiO5(OH)4
amrph.silica	-0.25	-2.98	-2.72	SiO2
andalusite	-7.46	9.54	17	Al2SiO5
anorthite	-7.61	20.03	27.64	CaAl2(SiO4)2
anthophyllite	0.27	69.2	68.93	Mg7Si8O22(OH)2
antigorite	86.28	268.29	182	Mg24Si17O42.5(OH)31
beidellit-ca	-0.82	5.88	6.7	Ca0.165Al2.33Si3.67O10(OH)2
beidellit-mg	-0.84	5.85	6.69	Mg0.165Al2.33Si3.67O10(OH)2
boehmite	-3.56	6.26	9.81	AlO2H
Ca(H2Tartrate):4H2O	1.32	-4.66	-5.98	Ca(H2Tartrate):4H2O
Ca(OH)2	-9.57	13.47	23.04	Ca(OH)2
ca-al	-14.1	23.01	37.11	CaAl2SiO6
ca-p	-1.95	18.25	20.2	CaAl2Si2.6O12.4H6.4
Ca2Al2O13H16	-19.18	39.45	58.63	Ca2Al2O13H16
Ca2Al2SiO15H16	-12.57	36.48	49.05	Ca2Al2SiO15H16
Ca2SiO4	-13.94	23.97	37.91	Ca2SiO4
Ca3Al2O12H12	-26.45	52.93	79.38	Ca3Al2O12H12

Ca3Al2Si0.5O12H10	-22.03	51.44	73.47	Ca3Al2Si0.5O12H10
Ca3SiO5	-37.35	37.44	74.79	Ca3SiO5
Ca4Al2O20H26	-35.9	66.4	102.3	Ca4Al2O20H26
CaO	-19.5	13.47	32.97	CaO
chabazite	0.5	14.08	13.58	CaAl2Si4O12:6H2O
chalcedony	0.58	-2.98	-3.55	SiO2
chamosite-7a	0.42	32.11	31.69	Fe2Al2SiO5(OH)4
chrysotile	1.9	33.91	32.01	Mg3Si2O5(OH)4
chs(0.8)	-3.23	7.8	11.03	Ca0.8SiO5H4.4
clinochl-14a	1.45	70.02	68.57	Mg5Al2Si3O10(OH)8
clinochl-7a	-1.92	70.02	71.94	Mg5Al2Si3O10(OH)8
clinozoisite	-7.92	36.79	44.71	Ca2Al3Si3O12(OH)
cordier.anh	-18.35	36.72	55.07	Mg2Al4Si5O18
cordier.hydr	-15.84	36.72	52.55	Mg2Al4Si5O18:H2O
corundum	-9.33	12.51	21.84	Al2O3
crist.beta_amorph	0.04	-2.98	-3.02	SiO2
cristobalite	0.49	-2.98	-3.47	SiO2
csh(1.1)	-4.82	11.84	16.66	Ca1.1SiO7H7.8
csh(1.8)	-11.14	21.27	32.41	Ca1.8SiO9H10.4
daphnite-14a	8.7	60.01	51.31	Fe5Al2Si3O10(OH)8
daphnite-7a	5.31	60.01	54.71	Fe5Al2Si3O10(OH)8
diaspore	-2.69	6.26	8.95	AlHO2
diopside	-0.47	20.81	21.28	CaMgSi2O6
enstatite	-1.34	10.31	11.65	MgSiO3
epistilbite	2.63	8.13	5.5	CaAl2Si6O16:5H2O
fayalite	0.28	19.6	19.32	Fe2SiO4
Fe(H2Tartrate):3H2O	1.39	-6.84	-8.23	Fe(H2Tartrate):3H2O
Fe(OH)2	-2.72	11.29	14.01	Fe(OH)2
FeO	-2.37	11.29	13.66	FeO
ferrosilite	0.78	8.31	7.53	FeSiO3
forsterite	-4.96	23.6	28.55	Mg2SiO4
foshagite	-14.45	44.96	59.41	Ca4Si3O9(OH)2:0.5H2O
gehlenite	-21.05	36.48	57.53	Ca2Al2SiO7
gibbsite	-1.67	6.26	7.93	Al(OH)3
greenalite	5.03	27.91	22.87	Fe3Si2O5(OH)4
grossular	-10.11	44	54.11	Ca3Al2(SiO4)3
gyrolite	-2.52	18.02	20.54	Ca2Si3O7(OH)2:1.5H2O
H2(g)	-24	-27.14	-3.14	H2
halloy	-3.92	6.56	10.48	Al2Si2O9H4
hedenbergite	-1.03	18.81	19.84	CaFe(SiO3)2
hercynite	-3.88	23.8	27.68	FeAl2O4
heulandite-ca	3.69	5.15	1.47	CaAl2Si7O18:6H2O
hillebrandite	-8.59	23.97	32.55	Ca2SiO3(OH)2:0.167H2O
hydrogarnet	-27.87	52.93	80.8	Ca3Al2O6:6H2O

hydrotalcite	-9.69	65.65	75.34	Mg4Al2O17H20
jennite	-43.21	103.39	146.6	Ca9Si6O32H22
kaolinite	-2.54	-39.82	-37.29	Al2Si2O5(OH)4
katoite	-18.94	49.95	68.89	Ca3Al2SiO12H8
kyanite	-7.18	9.54	16.72	Al2SiO5
larnite	-15.45	23.97	39.42	Ca2SiO4
laumontite	-1.32	14.08	15.4	CaAl2Si4O12:4H2O
lawsonite	-3.15	20.03	23.19	CaAl2Si2O7(OH)2:H2O
margarite	-10.57	32.54	43.12	CaAl4Si2O10(OH)2
merwinite	-21.33	47.75	69.08	MgCa3(SiO4)2
Mg(H2Tartrate):5H2O	-1.81	-4.84	-3.03	Mg(H2Tartrate):5H2O
Mg(OH)2	-3.77	13.29	17.05	Mg(OH)2
minnesotaite	7.81	21.96	14.15	Fe3Si4O10(OH)2
monticellite	-6.35	23.78	30.13	CaMgSiO4
montmor-ca	1.8	5.15	3.35	Ca0.165Mg0.33Al1.67Si4O10(OH)2
montmor-mg	1.84	5.12	3.28	Mg0.495Al1.67Si4O10(OH)2
O2(g)	-36.14	-39.08	-2.95	O2
okenite	2.45	7.52	5.07	CaSi2O4(OH)2:H2O
phillipsite-ca	1.5	11.11	9.6	CaAl2Si5O14:5H2O
prehnite	-3.38	30.53	33.91	Ca2Al2Si3O10(OH)2
pseudo	-3.7	10.5	14.2	CaSiO3
pyrophyllite	-0.74	0.61	1.35	Al2Si4O10(OH)2
quartz	1.07	-2.98	-4.05	SiO2
rankinite	-18.11	34.47	52.57	Ca3Si2O7
ripidolit-14a	5.76	66.02	60.25	Mg3Fe2Al2Si3O10(OH)8
ripidolit-7a	2.37	66.02	63.64	Mg3Fe2Al2Si3O10(OH)8
saponite-ca	5.91	33.23	27.32	Ca0.165Mg3Al0.33Si3.67O10(OH)2
saponite-h	4.67	31	26.33	H0.33Mg3Al0.33Si3.67O10(OH)2
saponite-mg	5.9	33.2	27.3	Mg3.165Al0.33Si3.67O10(OH)2
scolecite	1.92	17.06	15.14	CaAl2Si3O10:3H2O
sepiolite	1.53	-38.75	-40.28	Mg2Si3O7.5OH:3H2O
sillimantite	-7.83	9.54	17.36	Al2SiO5
spinel	-13.28	25.8	39.07	Al2MgO4
stratlingite	-12.85	36.48	49.33	Ca2Al2Si1O15H16
talc	5.99	27.96	21.97	Mg3Si4O10(OH)2
tobermorite(11A)	-17.32	49.51	66.83	Ca5Si6H11O22.5
tobermorite(14A)	-14.82	49.51	64.32	Ca5Si6H21O27.5
tobermorite(9A)	-20.28	49.51	69.79	Ca5Si6H6O20
tremolite	6.9	69.57	62.67	Ca2Mg5Si8O22(OH)2
wairakite	-5.84	14.08	19.92	CaAl2Si4O12:2H2O
wollastonite	-3.29	10.5	13.79	CaSiO3
xonotlite	-4.47	62.98	67.45	Ca6Si6O17(OH)2
yugawaralite	-0.12	4.07	4.18	Ca0.5AlSi3O8:2H2O
zoisite	-8.05	36.79	44.84	Ca2Al3(SiO4)3OH

temp	22		
pH	6.4		
pe	4		
density	0.997		
Si	5.349379		
Al	101.3402	umol/kgw	
Fe(2)	2.551242		
Ca	20.98602		
Mg	6.995342		
Ti(4)	7.59746	umol/kgw	
Citrate	0	mol/kgw	
water	1	#	kg
Elements	Molality		
Al	1.01E-04		
Ca	5.24E-04		
Fe(2)	4.57E-05		
Mg	2.88E-04		
Si	8.90E-05		
Ti(4)	7.60E-06		

Distribution of species

	Species		Molality	Activity	log Molality	log Activity	log Gamma	mole V cm³/mol
	H+		4.17E-07	3.98E-07	-6.38	-6.4	-0.02	0
	OH-		2.09E-08	2.00E-08	-7.679	-7.699	-0.02	0
	H2O		5.55E+01	1.00E+00	1.744	0	0	18.06
Al		1.01E-04						
	Al(OH)2+		4.59E-05	4.38E-05	-4.338	-4.359	-0.02	0
	Al(OH)4-		3.71E-05	3.54E-05	-4.431	-4.451	-0.02	0
	Al(OH)3		1.40E-05	1.40E-05	-4.856	-4.855	0	0
	AlOH+2		4.21E-06	3.50E-06	-5.376	-5.457	-0.081	0
	Al+3		2.11E-07	1.39E-07	-6.676	-6.858	-0.182	0
Ca		5.24E-04						
	Ca+2		5.24E-04	4.35E-04	-3.281	-3.362	-0.081	0
	Ca(OH)+		2.24E-10	2.14E-10	-9.65	-9.67	-0.02	0
Fe(2)		4.57E-05						
	Fe+2		4.57E-05	3.79E-05	-4.34	-4.421	-0.081	0
	Fe(OH)+		2.52E-08	2.40E-08	-7.599	-7.62	-0.02	0

	Fe(OH)2		3.68E-13	3.68E-13	-12.435	-12.434	0	0
	Fe(OH)3-		3.74E-17	3.57E-17	-16.427	-16.447	-0.02	0
	Fe(OH)4-2		1.82E-25	1.51E-25	-24.741	-24.821	-0.081	0
H(0)		2.31E-24						
	H2		1.16E-24	1.16E-24	-23.937	-23.937	0	0
Mg		2.88E-04						
	Mg+2		2.88E-04	2.39E-04	-3.541	-3.622	-0.081	0
	MgOH+		7.83E-10	7.47E-10	-9.106	-9.127	-0.02	0
O(0)		0.00E+00						
	O2		0.00E+00	0.00E+00	-45.484	-45.484	0	0
Si		8.90E-05						
	H4SiO4		8.90E-05	8.90E-05	-4.051	-4.05	0	0
	H3SiO4-		3.27E-08	3.12E-08	-7.486	-7.506	-0.02	0
	H5Si2O7-		1.66E-10	1.58E-10	-9.781	-9.801	-0.02	0
	H4Si2O7-2		6.02E-15	5.00E-15	-14.22	-14.301	-0.081	0
	H2SiO4-2		3.60E-15	2.99E-15	-14.443	-14.524	-0.081	0
	H5Si3O10-3		5.38E-21	3.54E-21	-20.27	-20.451	-0.182	0
	H3Si3O9-3		4.27E-22	2.81E-22	-21.37	-21.551	-0.182	0
	H5Si4O12-3		4.79E-23	3.15E-23	-22.32	-22.502	-0.182	0
	H4Si4O12-4		2.64E-27	1.25E-27	-26.579	-26.902	-0.323	0
Ti(4)		7.60E-06						
	Ti(OH)4		7.60E-06	7.60E-06	-5.119	-5.119	0	0

Saturation indices

Phase	SI**	log	IAP	log
afwillite	-26.89	20.21	47.1	Ca3Si2O4(OH)6
akermanite	-25.82	19.95	45.77	Ca2MgSi2O7
Al(OH)3(amorph.)	1.34	12.34	11	Al(OH)3
amesite-14a	7.63	38.99	31.36	Mg2Al2SiO5(OH)4
amrph.silica	-1.33	-4.05	-2.72	SiO2
andalusite	3.64	20.63	17	Al2SiO5
anorthite	-1.62	26.02	27.64	CaAl2(SiO4)2
anthophyllite	-37.09	31.84	68.93	Mg7Si8O22(OH)2
antigorite	-30.58	151.42	182	Mg24Si17O42.5(OH)31
beidellit-ca	8.74	15.45	6.7	Ca0.165Al2.33Si3.67O10(OH)2
beidellit-mg	8.72	15.41	6.69	Mg0.165Al2.33Si3.67O10(OH)2
boehmite	2.53	12.34	9.81	AlO2H
Ca(OH)2	-13.6	9.44	23.04	Ca(OH)2
ca-al	-7.04	30.07	37.11	CaAl2SiO6
ca-p	3.39	23.59	20.2	CaAl2Si2.6O12.4H6.4
Ca2Al2O13H16	-15.07	43.56	58.63	Ca2Al2O13H16
Ca2Al2SiO15H16	-9.54	39.51	49.05	Ca2Al2SiO15H16

Ca2SiO4	-23.08	14.83	37.91	Ca2SiO4
Ca3Al2O12H12	-26.38	53	79.38	Ca3Al2O12H12
Ca3Al2Si0.5O12H10	-22.5	50.97	73.47	Ca3Al2Si0.5O12H10
Ca3SiO5	-50.53	24.26	74.79	Ca3SiO5
Ca4Al2O20H26	-39.86	62.44	102.3	Ca4Al2O20H26
CaO	-23.54	9.44	32.97	CaO
chabazite	4.34	17.92	13.58	CaAl2Si4O12:6H2O
chalcedony	-0.5	-4.05	-3.55	SiO2
chamosite-7a	5.7	37.39	31.69	Fe2Al2SiO5(OH)4
chrysotile	-12.58	19.43	32.01	Mg3Si2O5(OH)4
chs(0.8)	-7.53	3.5	11.03	Ca0.8SiO5H4.4
clinochl-14a	-10.15	58.42	68.57	Mg5Al2Si3O10(OH)8
clinochl-7a	-13.52	58.42	71.94	Mg5Al2Si3O10(OH)8
clinozoisite	-0.96	43.75	44.71	Ca2Al3Si3O12(OH)
cordier.anh	-7.59	47.47	55.07	Mg2Al4Si5O18
cordier.hydr	-5.08	47.47	52.55	Mg2Al4Si5O18:H2O
corundum	2.84	24.68	21.84	Al2O3
crist.beta_amorph	-1.03	-4.05	-3.02	SiO2
cristobalite	-0.58	-4.05	-3.47	SiO2
csh(1.1)	-10.33	6.33	16.66	Ca1.1SiO7H7.8
csh(1.8)	-19.47	12.94	32.41	Ca1.8SiO9H10.4
daphnite-14a	3.12	54.43	51.31	Fe5Al2Si3O10(OH)8
daphnite-7a	-0.28	54.43	54.71	Fe5Al2Si3O10(OH)8
diaspore	3.4	12.34	8.95	AlHO2
diopside	-10.76	10.52	21.28	CaMgSi2O6
enstatite	-6.52	5.13	11.65	MgSiO3
epistilbite	4.32	9.82	5.5	CaAl2Si6O16:5H2O
fayalite	-6.61	12.71	19.32	Fe2SiO4
Fe(OH)2	-5.63	8.38	14.01	Fe(OH)2
FeO	-5.28	8.38	13.66	FeO
ferrosilite	-3.2	4.33	7.53	FeSiO3
forsterite	-14.25	14.31	28.55	Mg2SiO4
foshagite	-33.81	25.6	59.41	Ca4Si3O9(OH)2:0.5H2O
gehlenite	-18.02	39.51	57.53	Ca2Al2SiO7
gibbsite	4.41	12.34	7.93	Al(OH)3
greenalite	-5.84	17.04	22.87	Fe3Si2O5(OH)4
grossular	-13.26	40.85	54.11	Ca3Al2(SiO4)3
gyrolite	-13.81	6.73	20.54	Ca2Si3O7(OH)2:1.5H2O
H2(g)	-20.8	-23.94	-3.14	H2
halloy	6.1	16.58	10.48	Al2Si2O9H4
hedenbergite	-10.12	9.72	19.84	CaFe(SiO3)2
hercynite	5.38	33.06	27.68	FeAl2O4
heulandite-ca	4.3	5.77	1.47	CaAl2Si7O18:6H2O
hillebrandite	-17.73	14.83	32.55	Ca2SiO3(OH)2:0.167H2O

hydrogarnet	-27.8	53	80.8	Ca3Al2O6:6H2O
hydrotalcite	-13.94	61.4	75.34	Mg4Al2O17H20
jennite	-85.96	60.64	146.6	Ca9Si6O32H22
kaolinite	7.48	-29.8	-37.29	Al2Si2O5(OH)4
katoite	-19.94	48.95	68.89	Ca3Al2SiO12H8
kyanite	3.91	20.63	16.72	Al2SiO5
larnite	-24.59	14.83	39.42	Ca2SiO4
laumontite	2.52	17.92	15.4	CaAl2Si4O12:4H2O
lawsonite	2.83	26.02	23.19	CaAl2Si2O7(OH)2:H2O
margarite	7.59	50.7	43.12	CaAl4Si2O10(OH)2
merwinite	-39.69	29.39	69.08	MgCa3(SiO4)2
Mg(OH)2	-7.87	9.18	17.05	Mg(OH)2
minnesotaite	-5.21	8.93	14.15	Fe3Si4O10(OH)2
monticellite	-15.57	14.57	30.13	CaMgSiO4
montmor-ca	5.64	9	3.35	Ca0.165Mg0.33Al1.67Si4O10(OH)2
montmor-mg	5.67	8.95	3.28	Mg0.495Al1.67Si4O10(OH)2
O2(g)	-42.54	-45.48	-2.95	O2
okenite	-3.73	1.34	5.07	CaSi2O4(OH)2:H2O
phillipsite-ca	4.27	13.87	9.6	CaAl2Si5O14:5H2O
prehnite	-2.5	31.41	33.91	Ca2Al2Si3O10(OH)2
pseudo	-8.81	5.39	14.2	CaSiO3
pyrophyllite	7.13	8.48	1.35	Al2Si4O10(OH)2
quartz	-0.01	-4.05	-4.05	SiO2
rankinite	-32.36	20.21	52.57	Ca3Si2O7
ripidolit-14a	-3.43	56.82	60.25	Mg3Fe2Al2Si3O10(OH)8
ripidolit-7a	-6.82	56.82	63.64	Mg3Fe2Al2Si3O10(OH)8
saponite-ca	-9.02	18.3	27.32	Ca0.165Mg3Al0.33Si3.67O10(OH)2
saponite-h	-9.59	16.74	26.33	H0.33Mg3Al0.33Si3.67O10(OH)2
saponite-mg	-9.04	18.26	27.3	Mg3.165Al0.33Si3.67O10(OH)2
scolecite	6.83	21.97	15.14	CaAl2Si3O10:3H2O
sepiolite	-9.91	-50.19	-40.28	Mg2Si3O7.5OH:3H2O
sillimantite	3.27	20.63	17.36	Al2SiO5
spinel	-5.21	33.86	39.07	Al2MgO4
stratlingite	-9.82	39.51	49.33	Ca2Al2Si1O15H16
talc	-10.63	11.33	21.97	Mg3Si4O10(OH)2
tobermorite(11A)	-43.94	22.89	66.83	Ca5Si6H11O22.5
tobermorite(14A)	-41.43	22.89	64.32	Ca5Si6H21O27.5
tobermorite(9A)	-46.9	22.89	69.79	Ca5Si6H6O20
tremolite	-30.31	32.36	62.67	Ca2Mg5Si8O22(OH)2
wairakite	-2	17.92	19.92	CaAl2Si4O12:2H2O
wollastonite	-8.4	5.39	13.79	CaSiO3
xonotlite	-35.13	32.33	67.45	Ca6Si6O17(OH)2
yugawaralite	0.73	4.91	4.18	Ca0.5AlSi3O8:2H2O
zoisite	-1.09	43.75	44.84	Ca2Al3(SiO4)3OH

	temp	22						
	pH	6.4						
	pe	4						
	density	0.997						
	Si	18.27679						
	Al	11.91964						
	Fe(2)	15.89286						
	Ca	39.73214						
	Mg	14.30357						
	Ti(4)	1.033036						
	Citrate	0.001393	mol/kgw					
	water	1	#	kg				
	Elements	Molality						
	Al	4.42E-04						
	Ca	9.91E-04						
	Citrate	1.39E-03						
	Fe(2)	2.85E-04						
	Mg	5.89E-04						
	Si	3.04E-04						
	Ti(4)	2.16E-05						
Distribution of species								
	Species	Molality	Activity	log Molality	log Activity	log Gamma	mole V cm³/mol	
	H+	4.30E-07	3.98E-07	-6.367	-6.4	-0.033	0	
	OH-	2.16E-08	2.00E-08	-7.666	-7.699	-0.033	0	
	H2O	5.55E+01	1.00E+00	1.744	0	0	18.06	
Al 4.42E-04								
	Al(OH)(Citrate)-2	1.62E-04	1.19E-04	-3.79	-3.923	-0.133	0	
	Al(Citrate)-	8.13E-05	7.53E-05	-4.09	-4.123	-0.033	0	
	Al3(OH)4(HCitrate)3-4	3.99E-05	1.18E-05	-4.399	-4.93	-0.531	0	
	Al2(OH)2(HCitrate)2-2	2.41E-05	1.78E-05	-4.617	-4.75	-0.133	0	
	Al(Citrate)(HCitrate)-4	1.85E-05	5.43E-06	-4.734	-5.265	-0.531	0	
	Al(Citrate)2-5	6.24E-06	9.23E-07	-5.205	-6.035	-0.83	0	
	Al(HCitrate)2-3	5.42E-06	2.72E-06	-5.266	-5.565	-0.299	0	
	Al(HCitrate)	1.70E-07	1.70E-07	-6.771	-6.77	0.001	0	
	Al(OH)2+	1.81E-09	1.68E-09	-8.742	-8.776	-0.033	0	
	Al(OH)4-	1.46E-09	1.36E-09	-8.835	-8.868	-0.033	0	
	Al(OH)3	5.34E-10	5.34E-10	-9.273	-9.272	0.001	0	
	AlOH+2	1.82E-10	1.34E-10	-9.741	-9.873	-0.133	0	

	Al3(OH)4(Citrate)3-7	1.44E-10	3.39E-12	-9.843	-11.47	-1.627	0
	AlH(HCitrate)+	2.88E-11	2.67E-11	-10.54	-10.573	-0.033	0
	Al+3	1.06E-11	5.31E-12	-10.976	-11.275	-0.299	0
Ca	9.91E-04						
	Ca+2	7.38E-04	5.43E-04	-3.132	-3.265	-0.133	0
	Ca(HCitrate)-	2.52E-04	2.33E-04	-3.599	-3.632	-0.033	0
	CaH(HCitrate)	1.57E-06	1.57E-06	-5.804	-5.803	0.001	0
	Ca(OH)+	2.88E-10	2.67E-10	-9.54	-9.573	-0.033	0
	CaH2(HCitrate)+	3.01E-15	2.79E-15	-14.521	-14.554	-0.033	0
Citrate	1.39E-03						
	HCitrate-3	2.84E-04	1.43E-04	-3.546	-3.845	-0.299	0
	Ca(HCitrate)-	2.52E-04	2.33E-04	-3.599	-3.632	-0.033	0
	Fe(HCitrate)-	2.10E-04	1.94E-04	-3.679	-3.712	-0.033	0
	Al(OH)(Citrate)-2	1.62E-04	1.19E-04	-3.79	-3.923	-0.133	0
	Mg(HCitrate)-	1.38E-04	1.28E-04	-3.861	-3.894	-0.033	0
	Al(Citrate)-	8.13E-05	7.53E-05	-4.09	-4.123	-0.033	0
	Al3(OH)4(HCitrate)3-4	3.99E-05	1.18E-05	-4.399	-4.93	-0.531	0
	H(HCitrate)-2	3.35E-05	2.46E-05	-4.475	-4.608	-0.133	0
	Al2(OH)2(HCitrate)2-2	2.41E-05	1.78E-05	-4.617	-4.75	-0.133	0
	Al(Citrate)(HCitrate)-4	1.85E-05	5.43E-06	-4.734	-5.265	-0.531	0
	Al(Citrate)2-5	6.24E-06	9.23E-07	-5.205	-6.035	-0.83	0
	Al(HCitrate)2-3	5.42E-06	2.72E-06	-5.266	-5.565	-0.299	0
	CaH(HCitrate)	1.57E-06	1.57E-06	-5.804	-5.803	0.001	0
	FeH(HCitrate)	1.06E-06	1.06E-06	-5.976	-5.975	0.001	0
	MgH(HCitrate)	5.15E-07	5.16E-07	-6.288	-6.288	0.001	0
	FeH(HCitrate)2-3	4.66E-07	2.34E-07	-6.331	-6.63	-0.299	0
	Al(HCitrate)	1.70E-07	1.70E-07	-6.771	-6.77	0.001	0
	H4SiO4(HCitrate)-3	1.12E-07	5.60E-08	-6.953	-7.252	-0.299	0
	Fe2(Citrate)2-4	5.10E-09	1.50E-09	-8.292	-8.824	-0.531	0
	Al3(OH)4(Citrate)3-7	1.44E-10	3.39E-12	-9.843	-11.47	-1.627	0
	AlH(HCitrate)+	2.88E-11	2.67E-11	-10.54	-10.573	-0.033	0
	H2(HCitrate)-	5.54E-13	5.14E-13	-12.256	-12.289	-0.033	0
	CaH2(HCitrate)+	3.01E-15	2.79E-15	-14.521	-14.554	-0.033	0
	MgH2(HCitrate)+	9.21E-16	8.54E-16	-15.036	-15.069	-0.033	0
	FeH2(HCitrate)+	3.77E-16	3.50E-16	-15.423	-15.456	-0.033	0
	H3(HCitrate)	7.29E-21	7.30E-21	-20.137	-20.137	0.001	0
Fe(2)	2.85E-04						
	Fe(HCitrate)-	2.10E-04	1.94E-04	-3.679	-3.712	-0.033	0
	Fe+2	7.34E-05	5.41E-05	-4.134	-4.267	-0.133	0
	FeH(HCitrate)	1.06E-06	1.06E-06	-5.976	-5.975	0.001	0
	FeH(HCitrate)2-3	4.66E-07	2.34E-07	-6.331	-6.63	-0.299	0
	Fe(OH)+	3.70E-08	3.43E-08	-7.432	-7.465	-0.033	0

	Fe2(Citrate)2-4	5.10E-09	1.50E-09	-8.292	-8.824	-0.531	0
	Fe(OH)2	5.24E-13	5.25E-13	-12.281	-12.28	0.001	0
	FeH2(HCitrate)+	3.77E-16	3.50E-16	-15.423	-15.456	-0.033	0
	Fe(OH)3-	5.50E-17	5.10E-17	-16.26	-16.293	-0.033	0
	Fe(OH)4-2	2.92E-25	2.15E-25	-24.534	-24.667	-0.133	0
H(0)	2.31E-24						
	H2	1.16E-24	1.16E-24	-23.937	-23.937	0.001	0
Mg	5.89E-04						
	Mg+2	4.50E-04	3.32E-04	-3.347	-3.479	-0.133	0
	Mg(HCitrate)-	1.38E-04	1.28E-04	-3.861	-3.894	-0.033	0
	MgH(HCitrate)	5.15E-07	5.16E-07	-6.288	-6.288	0.001	0
	MgOH+	1.12E-09	1.04E-09	-8.951	-8.984	-0.033	0
	MgH2(HCitrate)+	9.21E-16	8.54E-16	-15.036	-15.069	-0.033	0
O(0)	0.00E+00						
	O2	0.00E+00	0.00E+00	-45.485	-45.484	0.001	0
Si	3.04E-04						
	H4SiO4	3.04E-04	3.04E-04	-3.517	-3.517	0.001	0
	H3SiO4-	1.15E-07	1.07E-07	-6.939	-6.972	-0.033	0
	H4SiO4(HCitrate)-3	1.12E-07	5.60E-08	-6.953	-7.252	-0.299	0
	H5Si2O7-	2.00E-09	1.85E-09	-8.7	-8.733	-0.033	0
	H4Si2O7-2	7.93E-14	5.84E-14	-13.101	-13.233	-0.133	0
	H2SiO4-2	1.39E-14	1.02E-14	-13.857	-13.99	-0.133	0
	H5Si3O10-3	2.81E-19	1.41E-19	-18.551	-18.85	-0.299	0
	H3Si3O9-3	2.23E-20	1.12E-20	-19.651	-19.95	-0.299	0
	H5Si4O12-3	8.56E-21	4.30E-21	-20.068	-20.367	-0.299	0
	H4Si4O12-4	5.82E-25	1.71E-25	-24.235	-24.767	-0.531	0
Ti(4)	2.16E-05						
	Ti(OH)4	2.16E-05	2.16E-05	-4.666	-4.666	0.001	0

Saturation indices

	Phase	SI**	log	IAP	log
	afwillite	-25.53	21.57	47.1	Ca3Si2O4(OH)6
	akermanite	-24.42	21.36	45.77	Ca2MgSi2O7
	Al(OH)3(amorph.)	-3.07	7.92	11	Al(OH)3
	amesite-14a	-0.38	30.97	31.36	Mg2Al2SiO5(OH)4
	amrph.silica	-0.79	-3.52	-2.72	SiO2
	andalusite	-4.66	12.33	17	Al2SiO5
	anorthite	-9.29	18.35	27.64	CaAl2(SiO4)2
	anthophyllite	-31.82	37.11	68.93	Mg7Si8O22(OH)2
	antigorite	-18.09	163.91	182	Mg24Si17O42.5(OH)31
	beidellit-ca	0.43	7.13	6.7	Ca0.165Al2.33Si3.67O10(OH)2
	beidellit-mg	0.41	7.1	6.69	Mg0.165Al2.33Si3.67O10(OH)2
	boehmite	-1.89	7.92	9.81	AlO2H

Ca(OH)2	-13.5	9.54	23.04	Ca(OH)2
ca-al	-15.24	21.87	37.11	CaAl2SiO6
ca-p	-3.96	16.24	20.2	CaAl2Si2.6O12.4H6.4
Ca2Al2O13H16	-23.71	34.92	58.63	Ca2Al2O13H16
Ca2Al2SiO15H16	-17.65	31.4	49.05	Ca2Al2SiO15H16
Ca2SiO4	-22.35	15.55	37.91	Ca2SiO4
Ca3(HCitrate)2	-0.45	-17.48	-17.03	Ca3(HCitrate)2
Ca3(HCitrate)2:4H2O	-5.64	-17.48	-11.84	Ca3(HCitrate)2:4H2O
Ca3Al2O12H12	-34.93	44.45	79.38	Ca3Al2O12H12
Ca3Al2Si0.5O12H10	-30.77	42.7	73.47	Ca3Al2Si0.5O12H10
Ca3SiO5	-49.7	25.09	74.79	Ca3SiO5
Ca4Al2O20H26	-48.31	53.99	102.3	Ca4Al2O20H26
CaH(HCitrate)	-2.12	-13.51	-11.39	CaH(HCitrate)
CaO	-23.44	9.54	32.97	CaO
chabazite	-2.26	11.32	13.58	CaAl2Si4O12:6H2O
chalcedony	0.04	-3.52	-3.55	SiO2
chamosite-7a	-2.29	29.4	31.69	Fe2Al2SiO5(OH)4
chrysotile	-11.08	20.93	32.01	Mg3Si2O5(OH)4
chs(0.8)	-6.92	4.11	11.03	Ca0.8SiO5H4.4
clinochl-14a	-16.67	51.9	68.57	Mg5Al2Si3O10(OH)8
clinochl-7a	-20.04	51.9	71.94	Mg5Al2Si3O10(OH)8
clinozoisite	-12.41	32.29	44.71	Ca2Al3Si3O12(OH)
cordier.anh	-22.31	32.76	55.07	Mg2Al4Si5O18
cordier.hydr	-19.8	32.76	52.55	Mg2Al4Si5O18:H2O
corundum	-5.99	15.85	21.84	Al2O3
crist.beta_amorph	-0.5	-3.52	-3.02	SiO2
cristobalite	-0.05	-3.52	-3.47	SiO2
csh(1.1)	-9.69	6.97	16.66	Ca1.1SiO7H7.8
csh(1.8)	-18.76	13.65	32.41	Ca1.8SiO9H10.4
daphnite-14a	-3.34	47.96	51.31	Fe5Al2Si3O10(OH)8
daphnite-7a	-6.74	47.96	54.71	Fe5Al2Si3O10(OH)8
diaspore	-1.02	7.92	8.95	AlHO2
diopside	-9.46	11.82	21.28	CaMgSi2O6
enstatite	-5.85	5.8	11.65	MgSiO3
epistilbite	-1.22	4.28	5.5	CaAl2Si6O16:5H2O
fayalite	-5.77	13.55	19.32	Fe2SiO4
Fe(OH)2	-5.48	8.53	14.01	Fe(OH)2
FeO	-5.13	8.53	13.66	FeO
ferrosilite	-2.52	5.02	7.53	FeSiO3
forsterite	-13.43	15.12	28.55	Mg2SiO4
foshagite	-31.82	27.59	59.41	Ca4Si3O9(OH)2:0.5H2O
gehlenite	-26.13	31.4	57.53	Ca2Al2SiO7
gibbsite	0	7.92	7.93	Al(OH)3
greenalite	-4.31	18.57	22.87	Fe3Si2O5(OH)4

grossular	-20.2	33.91	54.11	Ca3Al2(SiO4)3
gyrolite	-12.02	8.52	20.54	Ca2Si3O7(OH)2:1.5H2O
H2(g)	-20.8	-23.94	-3.14	H2
halloy	-1.66	8.82	10.48	Al2Si2O9H4
hedenbergite	-8.8	11.03	19.84	CaFe(SiO3)2
hercynite	-3.3	24.38	27.68	FeAl2O4
heulandite-ca	-0.7	0.77	1.47	CaAl2Si7O18:6H2O
hillebrandite	-17	15.55	32.55	Ca2SiO3(OH)2:0.167H2O
hydrogarnet	-36.35	44.45	80.8	Ca3Al2O6:6H2O
hydrotalcite	-22.21	53.13	75.34	Mg4Al2O17H20
jennite	-81.88	64.72	146.6	Ca9Si6O32H22
kaolinite	-0.28	-37.57	-37.29	Al2Si2O5(OH)4
katoite	-27.95	40.94	68.89	Ca3Al2SiO12H8
kyanite	-4.39	12.33	16.72	Al2SiO5
larnite	-23.86	15.55	39.42	Ca2SiO4
laumontite	-4.08	11.32	15.4	CaAl2Si4O12:4H2O
lawsonite	-4.84	18.35	23.19	CaAl2Si2O7(OH)2:H2O
margarite	-8.91	34.2	43.12	CaAl4Si2O10(OH)2
merwinite	-38.19	30.89	69.08	MgCa3(SiO4)2
Mg(OH)2	-7.73	9.32	17.05	Mg(OH)2
minnesotaite	-2.61	11.53	14.15	Fe3Si4O10(OH)2
monticellite	-14.79	15.34	30.13	CaMgSiO4
montmor-ca	0.46	3.82	3.35	Ca0.165Mg0.33Al1.67Si4O10(OH)2
montmor-mg	0.5	3.78	3.28	Mg0.495Al1.67Si4O10(OH)2
O2(g)	-42.54	-45.48	-2.95	O2
okenite	-2.57	2.5	5.07	CaSi2O4(OH)2:H2O
phillipsite-ca	-1.8	7.8	9.6	CaAl2Si5O14:5H2O
prehnite	-9.54	24.37	33.91	Ca2Al2Si3O10(OH)2
pseudo	-8.18	6.02	14.2	CaSiO3
pyrophyllite	0.43	1.78	1.35	Al2Si4O10(OH)2
quartz	0.53	-3.52	-4.05	SiO2
rankinite	-31	21.57	52.57	Ca3Si2O7
ripidolit-14a	-9.92	50.33	60.25	Mg3Fe2Al2Si3O10(OH)8
ripidolit-7a	-13.32	50.33	63.64	Mg3Fe2Al2Si3O10(OH)8
saponite-ca	-8.07	19.24	27.32	Ca0.165Mg3Al0.33Si3.67O10(OH)2
saponite-h	-8.66	17.67	26.33	H0.33Mg3Al0.33Si3.67O10(OH)2
saponite-mg	-8.09	19.21	27.3	Mg3.165Al0.33Si3.67O10(OH)2
scolecite	-0.31	14.83	15.14	CaAl2Si3O10:3H2O
sepiolite	-8.03	-48.31	-40.28	Mg2Si3O7.5OH:3H2O
sillimantite	-5.03	12.33	17.36	Al2SiO5
spinel	-13.9	25.17	39.07	Al2MgO4
stratlingite	-17.93	31.4	49.33	Ca2Al2Si1O15H16
talc	-8.07	13.9	21.97	Mg3Si4O10(OH)2
tobermorite(11A)	-40.26	26.58	66.83	Ca5Si6H11O22.5

tobermorite(14A)	-37.75	26.58	64.32	Ca5Si6H21O27.5
tobermorite(9A)	-43.21	26.58	69.79	Ca5Si6H6O20
tremolite	-25.13	37.54	62.67	Ca2Mg5Si8O22(OH)2
wairakite	-8.6	11.32	19.92	CaAl2Si4O12:2H2O
wollastonite	-7.77	6.02	13.79	CaSiO3
xonotlite	-31.34	36.11	67.45	Ca6Si6O17(OH)2
yugawaralite	-2.04	2.14	4.18	Ca0.5AlSi3O8:2H2O
zoisite	-12.54	32.29	44.84	Ca2Al3(SiO4)3OH

BG_TA_6

temp	22		
pH	6.4		
pe	4		
density	0.997		
Si	8.079268		
Al	3.716463		
Fe(2)	9.695122		
Ca	26.66159		
Mg	8.887195		
Ti(4)	11.47459	umol/kgw	
Tartrate	0.001181	mol/kgw	
water	1	#	kg
Elements	Molality		
Al	1.38E-04		
Ca	6.65E-04		
Fe(2)	1.74E-04		
Mg	3.66E-04		
Si	1.35E-04		
Tartrate	1.18E-03		
Ti(4)	1.15E-05		

Distribution of species

	Species		Molality	Activity	log Molality	log Activity	log Gamma	mole V cm³/mol
	H+		4.28E-07	3.98E-07	-6.369	-6.4	-0.031	0
	OH-		2.15E-08	2.00E-08	-7.668	-7.699	-0.031	0
	H2O		5.55E+01	1.00E+00	1.744	0	0	18.06
Al		1.38E-04						
	Al(Tartrate)-		1.15E-04	1.07E-04	-3.938	-3.969	-0.031	0
	Al(Tartrate)(HTartrate)-4		1.06E-05	3.37E-06	-4.976	-5.472	-0.496	0
	Al(HTartrate)		6.03E-06	6.04E-06	-5.219	-5.219	0	0
	Al(HTartrate)(H2Tartrate)-2		3.33E-06	2.50E-06	-5.478	-5.602	-0.124	0
	Al2(Tartrate)2-2		9.46E-07	7.11E-07	-6.024	-6.148	-0.124	0
	Al2(HTartrate)(Tartrate)-		2.06E-07	1.91E-07	-6.687	-6.718	-0.031	0
	Al(OH)2+		7.32E-08	6.82E-08	-7.135	-7.166	-0.031	0

	Al(OH)4-		5.92E-08	5.51E-08	-7.228	-7.259	-0.031	0
	Al(OH)3		2.17E-08	2.17E-08	-7.664	-7.663	0	0
	Al(Tartrate)(HTartrate)2-7		1.36E-08	4.12E-10	-7.867	-9.385	-1.518	0
	AlOH+2		7.24E-09	5.44E-09	-8.14	-8.264	-0.124	0
	Al(H2Tartrate)2-		5.61E-09	5.22E-09	-8.251	-8.282	-0.031	0
	Al2(HTartrate)2		1.24E-09	1.24E-09	-8.908	-8.908	0	0
	Al+3		4.10E-10	2.16E-10	-9.387	-9.666	-0.279	0
	Al(H2Tartrate)+		1.56E-10	1.45E-10	-9.808	-9.839	-0.031	0
	Al(Tartrate)2(HTartrate)-8		1.28E-12	1.33E-14	-11.892	-13.875	-1.983	0
	Al2(HTartrate)(H2Tartrate)+		2.15E-13	2.00E-13	-12.667	-12.698	-0.031	0
	Al(Tartrate)3-9		2.16E-18	6.68E-21	-17.666	-20.175	-2.509	0
Ca		6.65E-04						
	Ca+2		6.39E-04	4.80E-04	-3.195	-3.319	-0.124	0
	Ca(H2Tartrate)		2.64E-05	2.65E-05	-4.578	-4.577	0	0
	CaH(H2Tartrate)+		2.33E-08	2.17E-08	-7.634	-7.665	-0.031	0
	Ca(OH)+		2.54E-10	2.36E-10	-9.596	-9.627	-0.031	0
Fe(2)		1.74E-04						
	Fe+2		1.58E-04	1.19E-04	-3.8	-3.924	-0.124	0
	Fe(H2Tartrate)		1.52E-05	1.52E-05	-4.818	-4.817	0	0
	Fe(OH)+		8.10E-08	7.54E-08	-7.092	-7.123	-0.031	0
	Fe(OH)2		1.15E-12	1.16E-12	-11.938	-11.938	0	0
	Fe(OH)3-		1.20E-16	1.12E-16	-15.919	-15.95	-0.031	0
	Fe(OH)4-2		6.30E-25	4.74E-25	-24.201	-24.324	-0.124	0
H(0)		2.31E-24						
	H2		1.16E-24	1.16E-24	-23.937	-23.937	0	0
Mg		3.66E-04						
	Mg+2		3.60E-04	2.71E-04	-3.444	-3.567	-0.124	0
	Mg(H2Tartrate)		5.48E-06	5.49E-06	-5.261	-5.26	0	0
	MgH(H2Tartrate)+		7.14E-09	6.64E-09	-8.147	-8.178	-0.031	0
	MgOH+		9.09E-10	8.47E-10	-9.041	-9.072	-0.031	0
O(0)		0.00E+00						
	O2		0.00E+00	0.00E+00	-45.485	-45.484	0	0
Si		1.35E-04						
	H4SiO4		1.34E-04	1.35E-04	-3.872	-3.871	0	0
	H3SiO4-		5.06E-08	4.71E-08	-7.296	-7.327	-0.031	0
	H5Si2O7-		3.88E-10	3.61E-10	-9.411	-9.442	-0.031	0
	H4Si2O7-2		1.52E-14	1.14E-14	-13.818	-13.942	-0.124	0
	H2SiO4-2		6.02E-15	4.52E-15	-14.221	-14.345	-0.124	0
	H5Si3O10-3		2.32E-20	1.22E-20	-19.635	-19.913	-0.279	0
	H3Si3O9-3		1.84E-21	9.70E-22	-20.735	-21.013	-0.279	0
	H5Si4O12-3		3.12E-22	1.64E-22	-21.506	-21.784	-0.279	0
	H4Si4O12-4		2.05E-26	6.54E-27	-25.689	-26.184	-0.496	0
Tartrate		1.18E-03						
	H2Tartrate-2		9.79E-04	7.36E-04	-3.009	-3.133	-0.124	0

	Al(Tartrate)-		1.15E-04	1.07E-04	-3.938	-3.969	-0.031	0
	Ca(H2Tartrate)		2.64E-05	2.65E-05	-4.578	-4.577	0	0
	Fe(H2Tartrate)		1.52E-05	1.52E-05	-4.818	-4.817	0	0
	Al(Tartrate)(HTartrate)-4		1.06E-05	3.37E-06	-4.976	-5.472	-0.496	0
	Al(HTartrate)		6.03E-06	6.04E-06	-5.219	-5.219	0	0
	Mg(H2Tartrate)		5.48E-06	5.49E-06	-5.261	-5.26	0	0
	Al(HTartrate)(H2Tartrate)-2		3.33E-06	2.50E-06	-5.478	-5.602	-0.124	0
	H(H2Tartrate)-		2.96E-06	2.75E-06	-5.529	-5.56	-0.031	0
	Al2(Tartrate)2-2		9.46E-07	7.11E-07	-6.024	-6.148	-0.124	0
	Al2(HTartrate)(Tartrate)-		2.06E-07	1.91E-07	-6.687	-6.718	-0.031	0
	CaH(H2Tartrate)+		2.33E-08	2.17E-08	-7.634	-7.665	-0.031	0
	Al(Tartrate)(HTartrate)2-7		1.36E-08	4.12E-10	-7.867	-9.385	-1.518	0
	MgH(H2Tartrate)+		7.14E-09	6.64E-09	-8.147	-8.178	-0.031	0
	Al(H2Tartrate)2-		5.61E-09	5.22E-09	-8.251	-8.282	-0.031	0
	HTartrate-3		1.31E-09	6.87E-10	-8.884	-9.163	-0.279	0
	Al2(HTartrate)2		1.24E-09	1.24E-09	-8.908	-8.908	0	0
	H2(H2Tartrate)		7.34E-10	7.34E-10	-9.135	-9.134	0	0
	Al(H2Tartrate)+		1.56E-10	1.45E-10	-9.808	-9.839	-0.031	0
	Al(Tartrate)2(HTartrate)-8		1.28E-12	1.33E-14	-11.892	-13.875	-1.983	0
	Al2(HTartrate)(H2Tartrate)+		2.15E-13	2.00E-13	-12.667	-12.698	-0.031	0
	Tartrate-4		5.79E-16	1.85E-16	-15.237	-15.733	-0.496	0
	Al(Tartrate)3-9		2.16E-18	6.68E-21	-17.666	-20.175	-2.509	0
Ti(4)		1.15E-05						
	Ti(OH)4		1.15E-05	1.15E-05	-4.94	-4.94	0	0

Saturation indices

	Phase	SI**	log	IAP	log
	afwillite	-26.4	20.7	47.1	Ca3Si2O4(OH)6
	akermanite	########	2.05E+01	45.77	Ca2MgSi2O7
	Al(OH)3(amorph.)	-1.46	9.53	11	Al(OH)3
	amesite-14a	2.3	33.66	31.36	Mg2Al2SiO5(OH)4
	amrph.silica	-1.15	-3.87	-2.72	SiO2
	andalusite	-1.8	15.2	17	Al2SiO5
	anorthite	-6.83	20.81	27.64	CaAl2(SiO4)2
	anthophyllite	-35.27	33.66	68.93	Mg7Si8O22(OH)2
	antigorite	-26.23	155.77	182	Mg24Si17O42.5(OH)31
	beidellit-ca	2.87	9.57	6.7	Ca0.165Al2.33Si3.67O10(OH)2
	beidellit-mg	2.84	9.53	6.69	Mg0.165Al2.33Si3.67O10(OH)2
	boehmite	-0.28	9.53	9.81	AlO2H
	Ca(H2Tartrate):4H2O	-0.47	-6.45	-5.98	Ca(H2Tartrate):4H2O
	Ca(OH)2	-13.56	9.48	23.04	Ca(OH)2
	ca-al	-12.43	24.68	37.11	CaAl2SiO6
	ca-p	-1.72	18.48	20.2	CaAl2Si2.6O12.4H6.4

Ca2Al2O13H16	-20.6	38.03	58.63	Ca2Al2O13H16
Ca2Al2SiO15H16	-14.89	34.16	49.05	Ca2Al2SiO15H16
Ca2SiO4	-22.81	15.09	37.91	Ca2SiO4
Ca3Al2O12H12	-31.87	47.51	79.38	Ca3Al2O12H12
Ca3Al2Si0.5O12H10	-27.89	45.58	73.47	Ca3Al2Si0.5O12H10
Ca3SiO5	-50.22	24.57	74.79	Ca3SiO5
Ca4Al2O20H26	-45.31	56.99	102.3	Ca4Al2O20H26
CaO	-23.49	9.48	32.97	CaO
chabazite	-0.52	13.06	13.58	CaAl2Si4O12:6H2O
chalcedony	-0.32	-3.87	-3.55	SiO2
chamosite-7a	1.26	32.95	31.69	Fe2Al2SiO5(OH)4
chrysotile	-12.05	19.96	32.01	Mg3Si2O5(OH)4
chs(0.8)	-7.32	3.71	11.03	Ca0.8SiO5H4.4
clinochl-14a	-14.95	53.62	68.57	Mg5Al2Si3O10(OH)8
clinochl-7a	-18.32	53.62	71.94	Mg5Al2Si3O10(OH)8
clinozoisite	-8.76	35.95	44.71	Ca2Al3Si3O12(OH)
cordier.anh	-17.82	37.25	55.07	Mg2Al4Si5O18
cordier.hydr	-15.31	37.25	52.55	Mg2Al4Si5O18:H2O
corundum	-2.77	19.07	21.84	Al2O3
crist.beta_amorph	-0.85	-3.87	-3.02	SiO2
cristobalite	-0.4	-3.87	-3.47	SiO2
csh(1.1)	-10.1	6.56	16.66	Ca1.1SiO7H7.8
csh(1.8)	-19.21	13.2	32.41	Ca1.8SiO9H10.4
daphnite-14a	0.52	51.83	51.31	Fe5Al2Si3O10(OH)8
daphnite-7a	-2.87	51.83	54.71	Fe5Al2Si3O10(OH)8
diaspore	0.59	9.53	8.95	AlHO2
diopside	-10.31	10.97	21.28	CaMgSi2O6
enstatite	-6.29	5.36	11.65	MgSiO3
epistilbite	-0.18	5.32	5.5	CaAl2Si6O16:5H2O
fayalite	-5.44	13.88	19.32	Fe2SiO4
Fe(H2Tartrate):3H2O	1.17	-7.06	-8.23	Fe(H2Tartrate):3H2O
Fe(OH)2	-5.13	8.88	14.01	Fe(OH)2
FeO	-4.78	8.88	13.66	FeO
ferrosilite	-2.53	5	7.53	FeSiO3
forsterite	-13.96	14.59	28.55	Mg2SiO4
foshagite	-33.1	26.31	59.41	Ca4Si3O9(OH)2:0.5H2O
gehlenite	-23.37	34.16	57.53	Ca2Al2SiO7
gibbsite	1.61	9.53	7.93	Al(OH)3
greenalite	-3.99	18.88	22.87	Fe3Si2O5(OH)4
grossular	-18.21	35.9	54.11	Ca3Al2(SiO4)3
gyrolite	-13.19	7.35	20.54	Ca2Si3O7(OH)2:1.5H2O
H2(g)	-20.8	-23.94	-3.14	H2
halloy	0.85	11.33	10.48	Al2Si2O9H4
hedenbergite	-9.23	10.61	19.84	CaFe(SiO3)2

hercynite	0.26	27.94	27.68	FeAl2O4
heulandite-ca	-0.01	1.45	1.47	CaAl2Si7O18:6H2O
hillebrandite	-17.46	15.09	32.55	Ca2SiO3(OH)2:0.167H2O
hydrogarnet	-33.29	47.51	80.8	Ca3Al2O6:6H2O
hydrotalcite	-19.34	56	75.34	Mg4Al2O17H20
jennite	-84.49	62.11	146.6	Ca9Si6O32H22
kaolinite	2.23	-35.06	-37.29	Al2Si2O5(OH)4
katoite	-25.25	43.64	68.89	Ca3Al2SiO12H8
kyanite	-1.52	15.2	16.72	Al2SiO5
larnite	-24.33	15.09	39.42	Ca2SiO4
laumontite	-2.34	13.06	15.4	CaAl2Si4O12:4H2O
lawsonite	-2.38	20.81	23.19	CaAl2Si2O7(OH)2:H2O
margarite	-3.24	39.88	43.12	CaAl4Si2O10(OH)2
merwinite	-39.15	29.93	69.08	MgCa3(SiO4)2
Mg(H2Tartrate):5H2O	-3.67	-6.7	-3.03	Mg(H2Tartrate):5H2O
Mg(OH)2	-7.82	9.23	17.05	Mg(OH)2
minnesotaite	-3	11.14	14.15	Fe3Si4O10(OH)2
monticellite	-15.29	14.84	30.13	CaMgSiO4
montmor-ca	1.7	5.05	3.35	Ca0.165Mg0.33Al1.67Si4O10(OH)2
montmor-mg	1.73	5.01	3.28	Mg0.495Al1.67Si4O10(OH)2
O2(g)	-42.54	-45.48	-2.95	O2
okenite	-3.33	1.74	5.07	CaSi2O4(OH)2:H2O
phillipsite-ca	-0.41	9.19	9.6	CaAl2Si5O14:5H2O
prehnite	-7.49	26.42	33.91	Ca2Al2Si3O10(OH)2
pseudo	-8.58	5.61	14.2	CaSiO3
pyrophyllite	2.23	3.58	1.35	Al2Si4O10(OH)2
quartz	0.17	-3.87	-4.05	SiO2
rankinite	-31.87	20.7	52.57	Ca3Si2O7
ripidolit-14a	-7.35	52.9	60.25	Mg3Fe2Al2Si3O10(OH)8
ripidolit-7a	-10.74	52.9	63.64	Mg3Fe2Al2Si3O10(OH)8
saponite-ca	-9.11	18.2	27.32	Ca0.165Mg3Al0.33Si3.67O10(OH)2
saponite-h	-9.69	16.64	26.33	H0.33Mg3Al0.33Si3.67O10(OH)2
saponite-mg	-9.14	18.16	27.3	Mg3.165Al0.33Si3.67O10(OH)2
scolecite	1.8	16.94	15.14	CaAl2Si3O10:3H2O
sepiolite	-9.27	-49.55	-40.28	Mg2Si3O7.5OH:3H2O
sillimantite	-2.17	15.2	17.36	Al2SiO5
spinel	-10.77	28.3	39.07	Al2MgO4
stratlingite	-15.17	34.16	49.33	Ca2Al2Si1O15H16
talc	-9.75	12.21	21.97	Mg3Si4O10(OH)2
tobermorite(11A)	-42.65	24.18	66.83	Ca5Si6H11O22.5
tobermorite(14A)	-40.14	24.18	64.32	Ca5Si6H21O27.5
tobermorite(9A)	-45.61	24.18	69.79	Ca5Si6H6O20
tremolite	-28.52	34.16	62.67	Ca2Mg5Si8O22(OH)2
wairakite	-6.85	13.06	19.92	CaAl2Si4O12:2H2O

wollastonite	-8.18	5.61	13.79	CaSiO3
xonotlite	-33.79	33.66	67.45	Ca6Si6O17(OH)2
yugawaralite	-1.52	2.66	4.18	Ca0.5AlSi3O8:2H2O
zoisite	-8.89	35.95	44.84	Ca2Al3(SiO4)3OH

BFS_i

temp	22		
pH	10		
pe	4		
density	0.997		
Si	13.00887		
Al	148.0378	umol/kgw	
Fe(2)	6.99219	umol/kgw	
Ca	76.25887		
Mg	2.108333		
Ti(4)	12.01235	umol/kgw	
Citrate	0	mol/kgw	
water	1	#	kg
Elements	Molality		
Al	1.48E-04		
Ca	1.90E-03		
Fe(2)	6.99E-06		
Mg	8.67E-05		
Si	2.17E-04		
Ti(4)	1.20E-05		

Distribution of species

	Species		Molality	Activity	log Molality	log Activity	log Gamma	mole V cm³/mol
	OH-		8.53E-05	7.95E-05	-4.069	-4.099	-0.03	0
	H+		1.07E-10	1.00E-10	-9.97	-10	-0.03	0
	H2O		5.55E+01	1.00E+00	1.744	0	0	18.06
Al		1.48E-04						
	Al(OH)4-		1.48E-04	1.38E-04	-3.83	-3.86	-0.03	0
	Al(OH)3		1.37E-08	1.37E-08	-7.865	-7.864	0	0
	Al(OH)2+		1.16E-11	1.08E-11	-10.937	-10.968	-0.03	0
	AlOH+2		2.85E-16	2.16E-16	-15.545	-15.665	-0.12	0
	Al+3		4.02E-21	2.15E-21	-20.396	-20.667	-0.271	0
Ca		1.90E-03						
	Ca+2		1.90E-03	1.44E-03	-2.721	-2.842	-0.12	0
	Ca(OH)+		3.02E-06	2.82E-06	-5.52	-5.55	-0.03	0
Fe(2)		6.99E-06						
	Fe(OH)+		4.46E-06	4.16E-06	-5.351	-5.381	-0.03	0

	Fe+2		2.18E-06	1.65E-06	-5.662	-5.783	-0.12	0
	Fe(OH)2		2.53E-07	2.54E-07	-6.596	-6.596	0	0
	Fe(OH)3-		1.05E-07	9.81E-08	-6.978	-7.008	-0.03	0
	Fe(OH)4-2		2.18E-12	1.65E-12	-11.662	-11.783	-0.12	0
H(0)		1.46E-31						
	H2		7.29E-32	7.30E-32	-31.137	-31.137	0	0
Mg		8.67E-05						
	Mg+2		8.59E-05	6.51E-05	-4.066	-4.187	-0.12	0
	MgOH+		8.68E-07	8.10E-07	-6.061	-6.091	-0.03	0
O(0)		1.65E-31						
	O2		8.23E-32	8.24E-32	-31.085	-31.084	0	0
Si		2.17E-04						
	H3SiO4-		1.29E-04	1.20E-04	-3.89	-3.92	-0.03	0
	H4SiO4		8.61E-05	8.62E-05	-4.065	-4.064	0	0
	H5Si2O7-		6.33E-07	5.90E-07	-6.199	-6.229	-0.03	0
	H4Si2O7-2		9.81E-08	7.43E-08	-7.008	-7.129	-0.12	0
	H2SiO4-2		6.06E-08	4.59E-08	-7.217	-7.338	-0.12	0
	H5Si3O10-3		3.78E-10	2.03E-10	-9.422	-9.693	-0.271	0
	H3Si3O9-3		3.01E-11	1.61E-11	-10.522	-10.793	-0.271	0
	H5Si4O12-3		3.26E-12	1.75E-12	-11.487	-11.758	-0.271	0
	H4Si4O12-4		8.40E-13	2.77E-13	-12.076	-12.558	-0.482	0
Ti(4)		1.20E-05						
	Ti(OH)4		1.20E-05	1.20E-05	-4.92	-4.92	0	0

Saturation indices

Phase	SI**	log	IAP	log
afwillite	-3.75	43.35	47.1	Ca3Si2O4(OH)6
akermanite	-3.77	42	45.77	Ca2MgSi2O7
Al(OH)3(amorph.)	-1.66	9.33	11	Al(OH)3
amesite-14a	14.87	46.23	31.36	Mg2Al2SiO5(OH)4
amrph.silica	-1.34	-4.06	-2.72	SiO2
andalusite	-2.4	14.6	17	Al2SiO5
anorthite	0.05	27.7	27.64	CaAl2(SiO4)2
anthophyllite	9.25	78.18	68.93	Mg7Si8O22(OH)2
antigorite	128.42	310.43	182	Mg24Si17O42.5(OH)31
beidellit-ca	2.96	9.66	6.7	Ca0.165Al2.33Si3.67O10(OH)2
beidellit-mg	2.75	9.44	6.69	Mg0.165Al2.33Si3.67O10(OH)2
boehmite	-0.48	9.33	9.81	AlO2H
Ca(OH)2	-5.88	17.16	23.04	Ca(OH)2
ca-al	-5.35	31.76	37.11	CaAl2SiO6
ca-p	5.06	25.26	20.2	CaAl2Si2.6O12.4H6.4
Ca2Al2O13H16	-5.65	52.98	58.63	Ca2Al2O13H16
Ca2Al2SiO15H16	-0.13	48.92	49.05	Ca2Al2SiO15H16

Ca2SiO4	-7.65	30.25	37.91	Ca2SiO4
Ca3Al2O12H12	-9.24	70.14	79.38	Ca3Al2O12H12
Ca3Al2Si0.5O12H10	-5.36	68.11	73.47	Ca3Al2Si0.5O12H10
Ca3SiO5	-27.38	47.41	74.79	Ca3SiO5
Ca4Al2O20H26	-15	87.3	102.3	Ca4Al2O20H26
CaO	-15.82	17.16	32.97	CaO
chabazite	5.99	19.57	13.58	CaAl2Si4O12:6H2O
chalcedony	-0.51	-4.06	-3.55	SiO2
chamosite-7a	11.35	43.04	31.69	Fe2Al2SiO5(OH)4
chrysotile	7.3	39.31	32.01	Mg3Si2O5(OH)4
chs(0.8)	-1.37	9.66	11.03	Ca0.8SiO5H4.4
clinochl-14a	16.97	85.54	68.57	Mg5Al2Si3O10(OH)8
clinochl-7a	13.6	85.54	71.94	Mg5Al2Si3O10(OH)8
clinozoisite	5.42	50.12	44.71	Ca2Al3Si3O12(OH)
cordier.anh	-6.43	48.64	55.07	Mg2Al4Si5O18
cordier.hydr	-3.92	48.64	52.55	Mg2Al4Si5O18:H2O
corundum	-3.17	18.67	21.84	Al2O3
crist.beta_amorph	-1.04	-4.06	-3.02	SiO2
cristobalite	-0.6	-4.06	-3.47	SiO2
csh(1.1)	-1.85	14.81	16.66	Ca1.1SiO7H7.8
csh(1.8)	-5.59	26.82	32.41	Ca1.8SiO9H10.4
daphnite-14a	26.25	77.56	51.31	Fe5Al2Si3O10(OH)8
daphnite-7a	22.85	77.56	54.71	Fe5Al2Si3O10(OH)8
diaspore	0.39	9.33	8.95	AlHO2
diopside	3.57	24.84	21.28	CaMgSi2O6
enstatite	0.1	11.75	11.65	MgSiO3
epistilbite	5.93	11.44	5.5	CaAl2Si6O16:5H2O
fayalite	5.05	24.37	19.32	Fe2SiO4
Fe(OH)2	0.21	14.22	14.01	Fe(OH)2
FeO	0.56	14.22	13.66	FeO
ferrosilite	2.62	10.15	7.53	FeSiO3
forsterite	-0.99	27.56	28.55	Mg2SiO4
foshagite	-2.97	56.44	59.41	Ca4Si3O9(OH)2:0.5H2O
gehlenite	-8.61	48.92	57.53	Ca2Al2SiO7
gibbsite	1.41	9.33	7.93	Al(OH)3
greenalite	11.65	34.52	22.87	Fe3Si2O5(OH)4
grossular	3.84	57.95	54.11	Ca3Al2(SiO4)3
gyrolite	1.59	22.12	20.54	Ca2Si3O7(OH)2:1.5H2O
H2(g)	-28	-31.14	-3.14	H2
halloy	0.06	10.54	10.48	Al2Si2O9H4
hedenbergite	3.41	23.25	19.84	CaFe(SiO3)2
hercynite	5.2	32.88	27.68	FeAl2O4
heulandite-ca	5.91	7.37	1.47	CaAl2Si7O18:6H2O
hillebrandite	-2.3	30.25	32.55	Ca2SiO3(OH)2:0.167H2O

hydrogarnet	-10.66	70.14	80.8	Ca3Al2O6:6H2O
hydrotalcite	6.58	81.92	75.34	Mg4Al2O17H20
jennite	-16.56	130.04	146.6	Ca9Si6O32H22
kaolinite	1.44	-35.85	-37.29	Al2Si2O5(OH)4
katoite	-2.81	66.08	68.89	Ca3Al2SiO12H8
kyanite	-2.12	14.6	16.72	Al2SiO5
larnite	-9.17	30.25	39.42	Ca2SiO4
laumontite	4.16	19.57	15.4	CaAl2Si4O12:4H2O
lawsonite	4.51	27.7	23.19	CaAl2Si2O7(OH)2:H2O
margarite	3.25	46.36	43.12	CaAl4Si2O10(OH)2
merwinite	-9.93	59.16	69.08	MgCa3(SiO4)2
Mg(OH)2	-1.24	15.81	17.05	Mg(OH)2
minnesotaite	12.25	26.39	14.15	Fe3Si4O10(OH)2
monticellite	-1.23	28.91	30.13	CaMgSiO4
montmor-ca	4.02	7.38	3.35	Ca0.165Mg0.33Al1.67Si4O10(OH)2
montmor-mg	3.88	7.16	3.28	Mg0.495Al1.67Si4O10(OH)2
O2(g)	-28.14	-31.08	-2.95	O2
okenite	3.96	9.03	5.07	CaSi2O4(OH)2:H2O
phillipsite-ca	5.9	15.5	9.6	CaAl2Si5O14:5H2O
prehnite	6.88	40.79	33.91	Ca2Al2Si3O10(OH)2
pseudo	-1.1	13.09	14.2	CaSiO3
pyrophyllite	1.06	2.41	1.35	Al2Si4O10(OH)2
quartz	-0.02	-4.06	-4.05	SiO2
rankinite	-9.23	43.35	52.57	Ca3Si2O7
ripidolit-14a	22.1	82.35	60.25	Mg3Fe2Al2Si3O10(OH)8
ripidolit-7a	18.7	82.35	63.64	Mg3Fe2Al2Si3O10(OH)8
saponite-ca	11.12	38.43	27.32	Ca0.165Mg3Al0.33Si3.67O10(OH)2
saponite-h	9.27	35.6	26.33	H0.33Mg3Al0.33Si3.67O10(OH)2
saponite-mg	10.91	38.21	27.3	Mg3.165Al0.33Si3.67O10(OH)2
scolecite	8.49	23.63	15.14	CaAl2Si3O10:3H2O
sepiolite	3.31	-36.96	-40.28	Mg2Si3O7.5OH:3H2O
sillimantite	-2.76	14.6	17.36	Al2SiO5
spinel	-4.59	34.48	39.07	Al2MgO4
stratlingite	-0.41	48.92	49.33	Ca2Al2Si1O15H16
talc	9.22	31.18	21.97	Mg3Si4O10(OH)2
tobermorite(11A)	-5.43	61.4	66.83	Ca5Si6H11O22.5
tobermorite(14A)	-2.92	61.4	64.32	Ca5Si6H21O27.5
tobermorite(9A)	-8.38	61.4	69.79	Ca5Si6H6O20
tremolite	18.2	80.87	62.67	Ca2Mg5Si8O22(OH)2
wairakite	-0.35	19.57	19.92	CaAl2Si4O12:2H2O
wollastonite	-0.7	13.09	13.79	CaSiO3
xonotlite	11.11	78.56	67.45	Ca6Si6O17(OH)2
yugawaralite	1.54	5.72	4.18	Ca0.5AlSi3O8:2H2O
zoisite	5.28	50.12	44.84	Ca2Al3(SiO4)3OH

	temp	22					
	pH	8					
	pe	4					
	density	0.997					
	Si	42.16625					
	Al	479.842	umol/kgw				
	Fe(2)	22.66411	umol/kgw				
	Ca	843.3249					
	Mg	144.068					
	Ti(4)	38.9362	umol/kgw				
	Citrate	0	mol/kgw				
	water	1	#	kg			
	Elements	Molality					
	Al	4.80E-04					
	Ca	2.10E-02					
	Fe(2)	2.27E-05					
	Mg	5.93E-03					
	Si	7.02E-04					
	Ti(4)	3.89E-05					

Distribution of species

	Species		Molality	Activity	log Molality	log Activity	log Gamma	mole V cm³/mol
	OH-		9.73E-07	7.95E-07	-6.012	-6.1	-0.088	0
	H+		1.22E-08	1.00E-08	-7.912	-8	-0.088	0
	H2O		5.55E+01	1.00E+00	1.744	0	0	18.06
Al		4.80E-04						
	Al(OH)4-		4.76E-04	3.89E-04	-3.323	-3.41	-0.088	0
	Al(OH)3		3.80E-06	3.85E-06	-5.42	-5.415	0.005	0
	Al(OH)2+		3.72E-07	3.04E-07	-6.43	-6.518	-0.088	0
	AlOH+2		1.36E-09	6.09E-10	-8.865	-9.215	-0.35	0
	Al+3		3.73E-12	6.07E-13	-11.429	-12.217	-0.788	0
Ca		2.10E-02						
	Ca+2		2.10E-02	9.39E-03	-1.677	-2.027	-0.35	0
	Ca(OH)+		2.25E-07	1.84E-07	-6.648	-6.736	-0.088	0
Fe(2)		2.27E-05						
	Fe+2		2.24E-05	9.98E-06	-4.651	-5.001	-0.35	0
	Fe(OH)+		3.08E-07	2.52E-07	-6.512	-6.6	-0.088	0
	Fe(OH)2		1.51E-10	1.53E-10	-9.82	-9.814	0.005	0
	Fe(OH)3-		7.25E-13	5.93E-13	-12.14	-12.227	-0.088	0
	Fe(OH)4-2		2.23E-19	9.96E-20	-18.651	-19.002	-0.35	0

H(0)		1.44E-27						
	H2		7.21E-28	7.30E-28	-27.142	-27.137	0.005	0
Mg		5.93E-03						
	Mg+2		5.93E-03	2.65E-03	-2.227	-2.578	-0.35	0
	MgOH+		4.03E-07	3.29E-07	-6.395	-6.483	-0.088	0
O(0)		1.63E-39						
	O2		8.13E-40	8.23E-40	-39.09	-39.085	0.005	0
Si		7.02E-04						
	H4SiO4		6.89E-04	6.98E-04	-3.162	-3.156	0.005	0
	H3SiO4-		1.19E-05	9.73E-06	-4.924	-5.012	-0.088	0
	H5Si2O7-		4.73E-07	3.87E-07	-6.325	-6.413	-0.088	0
	H4Si2O7-2		1.09E-09	4.87E-10	-8.962	-9.313	-0.35	0
	H2SiO4-2		8.33E-11	3.72E-11	-10.08	-10.43	-0.35	0
	H5Si3O10-3		6.60E-13	1.07E-13	-12.181	-12.969	-0.788	0
	H3Si3O9-3		5.24E-14	8.54E-15	-13.28	-14.069	-0.788	0
	H5Si4O12-3		4.61E-14	7.50E-15	-13.336	-14.125	-0.788	0
	H4Si4O12-4		3.00E-16	1.19E-17	-15.523	-16.925	-1.401	0
Ti(4)		3.89E-05						
	Ti(OH)4		3.89E-05	3.94E-05	-4.41	-4.404	0.005	0

Saturation indices

Phase	SI**	log	IAP	log
afwillite	-11.49	35.6	47.1	Ca3Si2O4(OH)6
akermanite	-10.72	35.06	45.77	Ca2MgSi2O7
Al(OH)3(amorph.)	0.79	11.78	11	Al(OH)3
amesite-14a	15.9	47.25	31.36	Mg2Al2SiO5(OH)4
amrph.silica	-0.43	-3.16	-2.72	SiO2
andalusite	3.41	20.41	17	Al2SiO5
anorthite	3.59	31.23	27.64	CaAl2(SiO4)2
anthophyllite	-0.23	68.71	68.93	Mg7Si8O22(OH)2
antigorite	86.48	268.48	182	Mg24Si17O42.5(OH)31
beidellit-ca	11.47	18.18	6.7	Ca0.165Al2.33Si3.67O10(OH)2
beidellit-mg	11.4	18.09	6.69	Mg0.165Al2.33Si3.67O10(OH)2
boehmite	1.97	11.78	9.81	AlO2H
Ca(OH)2	-9.07	13.97	23.04	Ca(OH)2
ca-al	-2.73	34.38	37.11	CaAl2SiO6
ca-p	9.13	29.33	20.2	CaAl2Si2.6O12.4H6.4
Ca2Al2O13H16	-7.12	51.51	58.63	Ca2Al2O13H16
Ca2Al2SiO15H16	-0.7	48.35	49.05	Ca2Al2SiO15H16
Ca2SiO4	-13.12	24.79	37.91	Ca2SiO4
Ca3Al2O12H12	-13.9	65.48	79.38	Ca3Al2O12H12
Ca3Al2Si0.5O12H10	-9.57	63.9	73.47	Ca3Al2Si0.5O12H10

Ca3SiO5	-36.03	38.76	74.79	Ca3SiO5
Ca4Al2O20H26	-22.85	79.45	102.3	Ca4Al2O20H26
CaO	-19	13.97	32.97	CaO
chabazite	11.33	24.91	13.58	CaAl2Si4O12:6H2O
chalcedony	0.4	-3.16	-3.55	SiO2
chamosite-7a	10.72	42.41	31.69	Fe2Al2SiO5(OH)4
chrysotile	1.94	33.95	32.01	Mg3Si2O5(OH)4
chs(0.8)	-3.01	8.02	11.03	Ca0.8SiO5H4.4
clinochl-14a	12.64	81.21	68.57	Mg5Al2Si3O10(OH)8
clinochl-7a	9.27	81.21	71.94	Mg5Al2Si3O10(OH)8
clinozoisite	9.12	53.83	44.71	Ca2Al3Si3O12(OH)
cordier.anh	3.13	58.2	55.07	Mg2Al4Si5O18
cordier.hydr	5.64	58.2	52.55	Mg2Al4Si5O18:H2O
corundum	1.73	23.57	21.84	Al2O3
crist.beta_amorph	-0.14	-3.16	-3.02	SiO2
cristobalite	0.31	-3.16	-3.47	SiO2
csh(1.1)	-4.45	12.21	16.66	Ca1.1SiO7H7.8
csh(1.8)	-10.42	21.99	32.41	Ca1.8SiO9H10.4
daphnite-14a	17.78	69.09	51.31	Fe5Al2Si3O10(OH)8
daphnite-7a	14.38	69.09	54.71	Fe5Al2Si3O10(OH)8
diaspore	2.84	11.78	8.95	AlHO2
diopside	-0.19	21.08	21.28	CaMgSi2O6
enstatite	-1.39	10.27	11.65	MgSiO3
epistilbite	13.1	18.6	5.5	CaAl2Si6O16:5H2O
fayalite	-0.47	18.84	19.32	Fe2SiO4
Fe(OH)2	-3.01	11	14.01	Fe(OH)2
FeO	-2.66	11	13.66	FeO
ferrosilite	0.31	7.84	7.53	FeSiO3
forsterite	-4.86	23.69	28.55	Mg2SiO4
foshagite	-12.99	46.42	59.41	Ca4Si3O9(OH)2:0.5H2O
gehlenite	-9.17	48.35	57.53	Ca2Al2SiO7
gibbsite	3.85	11.78	7.93	Al(OH)3
greenalite	3.81	26.68	22.87	Fe3Si2O5(OH)4
grossular	1.91	56.02	54.11	Ca3Al2(SiO4)3
gyrolite	-2.06	18.48	20.54	Ca2Si3O7(OH)2:1.5H2O
H2(g)	-24	-27.14	-3.14	H2
halloy	6.77	17.25	10.48	Al2Si2O9H4
hedenbergite	-1.18	18.66	19.84	CaFe(SiO3)2
hercynite	6.88	34.56	27.68	FeAl2O4
heulandite-ca	13.98	15.44	1.47	CaAl2Si7O18:6H2O
hillebrandite	-7.77	24.79	32.55	Ca2SiO3(OH)2:0.167H2O
hydrogarnet	-15.32	65.48	80.8	Ca3Al2O6:6H2O
hydrotalcite	1.91	77.25	75.34	Mg4Al2O17H20
jennite	-39.79	106.81	146.6	Ca9Si6O32H22

kaolinite	8.15	-29.13	-37.29	Al2Si2O5(OH)4
katoite	-6.56	62.33	68.89	Ca3Al2SiO12H8
kyanite	3.69	20.41	16.72	Al2SiO5
larnite	-14.63	24.79	39.42	Ca2SiO4
laumontite	9.51	24.91	15.4	CaAl2Si4O12:4H2O
lawsonite	8.04	31.23	23.19	CaAl2Si2O7(OH)2:H2O
margarite	11.68	54.79	43.12	CaAl4Si2O10(OH)2
merwinite	-20.06	49.03	69.08	MgCa3(SiO4)2
Mg(OH)2	-3.63	13.42	17.05	Mg(OH)2
minnesotaite	6.23	20.37	14.15	Fe3Si4O10(OH)2
monticellite	-5.89	24.24	30.13	CaMgSiO4
montmor-ca	10.43	13.79	3.35	Ca0.165Mg0.33Al1.67Si4O10(OH)2
montmor-mg	10.42	13.7	3.28	Mg0.495Al1.67Si4O10(OH)2
O2(g)	-36.14	-39.08	-2.95	O2
okenite	2.59	7.66	5.07	CaSi2O4(OH)2:H2O
phillipsite-ca	12.16	21.76	9.6	CaAl2Si5O14:5H2O
prehnite	8.13	42.04	33.91	Ca2Al2Si3O10(OH)2
pseudo	-3.38	10.82	14.2	CaSiO3
pyrophyllite	9.59	10.94	1.35	Al2Si4O10(OH)2
quartz	0.89	-3.16	-4.05	SiO2
rankinite	-16.97	35.61	52.57	Ca3Si2O7
ripidolit-14a	16.11	76.36	60.25	Mg3Fe2Al2Si3O10(OH)8
ripidolit-7a	12.72	76.36	63.64	Mg3Fe2Al2Si3O10(OH)8
saponite-ca	7.56	34.88	27.32	Ca0.165Mg3Al0.33Si3.67O10(OH)2
saponite-h	6.24	32.57	26.33	H0.33Mg3Al0.33Si3.67O10(OH)2
saponite-mg	7.49	34.79	27.3	Mg3.165Al0.33Si3.67O10(OH)2
scolecite	12.93	28.07	15.14	CaAl2Si3O10:3H2O
sepiolite	1.26	-39.02	-40.28	Mg2Si3O7.5OH:3H2O
sillimantite	3.05	20.41	17.36	Al2SiO5
spinel	-2.09	36.99	39.07	Al2MgO4
stratlingite	-0.98	48.35	49.33	Ca2Al2Si1O15H16
talc	5.68	27.64	21.97	Mg3Si4O10(OH)2
tobermorite(11A)	-15.91	50.93	66.83	Ca5Si6H11O22.5
tobermorite(14A)	-13.4	50.92	64.32	Ca5Si6H21O27.5
tobermorite(9A)	-18.86	50.93	69.79	Ca5Si6H6O20
tremolite	7.14	69.81	62.67	Ca2Mg5Si8O22(OH)2
wairakite	5	24.91	19.92	CaAl2Si4O12:2H2O
wollastonite	-2.97	10.82	13.79	CaSiO3
xonotlite	-2.55	64.9	67.45	Ca6Si6O17(OH)2
yugawaralite	5.12	9.3	4.18	Ca0.5AlSi3O8:2H2O
zoisite	8.99	53.83	44.84	Ca2Al3(SiO4)3OH

temp	22		
pH	8		
pe	4		
density	0.997		
Si	63.61141		
Al	723.88	umol/kgw	
Fe(2)	4.11		
Ca	362.95		
Mg	127.222		
Ti(4)	58.73861	umol/kgw	
Tartrate	0.0229	mol/kgw	
water	1	#	kg

Elements	Molality
Al	7.24E-04
Ca	9.06E-03
Fe(2)	7.36E-05
Mg	5.23E-03
Si	1.06E-03
Tartrate	2.29E-02
Ti(4)	5.87E-05

Distribution of species

	Species		Molality	Activity	log Molality	log Activity	log Gamma	mole V cm³/mo
	OH-		1.00E-06	7.95E-07	-6	-6.1	-0.1	
	H+		1.26E-08	1.00E-08	-7.9	-8	-0.1	
	H2O		5.55E+01	9.99E-01	1.744	0	0	18.0
Al		7.24E-04						
	Al(Tartrate)(HTartrate)2-7		6.89E-04	9.02E-09	-3.162	-8.045	-4.883	
	Al(Tartrate)2(HTartrate)-8		2.77E-05	1.16E-11	-4.557	-10.935	-6.378	
	Al(Tartrate)(HTartrate)-4		7.19E-06	1.83E-07	-5.143	-6.738	-1.594	
	Al(Tartrate)3-9		2.74E-08	2.32E-16	-7.563	-15.635	-8.072	
	Al(Tartrate)-		1.82E-08	1.44E-08	-7.741	-7.841	-0.1	
	Al(OH)4-		1.45E-09	1.16E-09	-8.838	-8.937	-0.1	
	Al(HTartrate)(H2Tartrate)-2		2.14E-10	8.56E-11	-9.669	-10.068	-0.399	
	Al(HTartrate)		2.00E-11	2.04E-11	-10.699	-10.691	0.008	
	Al(OH)3		1.12E-11	1.14E-11	-10.95	-10.942	0.008	
	Al(OH)2+		1.14E-12	9.02E-13	-11.945	-12.045	-0.1	
	Al2(Tartrate)2-2		3.22E-14	1.28E-14	-13.493	-13.891	-0.399	
	Al(H2Tartrate)2-		5.65E-15	4.49E-15	-14.248	-14.348	-0.1	
	AlOH+2		4.53E-15	1.81E-15	-14.344	-14.742	-0.399	
	Al2(HTartrate)(Tartrate)-		1.09E-16	8.68E-17	-15.962	-16.061	-0.1	
	Al(H2Tartrate)+		1.55E-17	1.23E-17	-16.811	-16.911	-0.1	

	Al+3		1.42E-17	1.80E-18	-16.847	-17.744	-0.897
	Al2(HTartrate)2		1.38E-20	1.41E-20	-19.859	-19.851	0.008
	Al2(HTartrate)(H2Tartrate)+		7.22E-26	5.74E-26	-25.142	-25.241	-0.1
Ca		9.06E-03					
	Ca+2		7.43E-03	2.97E-03	-2.129	-2.528	-0.399
	Ca(H2Tartrate)		1.63E-03	1.66E-03	-2.788	-2.78	0.008
	Ca(OH)+		7.30E-08	5.80E-08	-7.137	-7.236	-0.1
	CaH(H2Tartrate)+		4.29E-08	3.41E-08	-7.368	-7.468	-0.1
Fe(2)		7.36E-05					
	Fe+2		4.84E-05	1.93E-05	-4.315	-4.714	-0.399
	Fe(H2Tartrate)		2.46E-05	2.51E-05	-4.609	-4.601	0.008
	Fe(OH)+		6.13E-07	4.87E-07	-6.213	-6.313	-0.1
	Fe(OH)2		2.91E-10	2.97E-10	-9.536	-9.528	0.008
	Fe(OH)3-		1.44E-12	1.15E-12	-11.841	-11.94	-0.1
	Fe(OH)4-2		4.83E-19	1.93E-19	-18.316	-18.715	-0.399
H(0)		1.43E-27					
	H2		7.16E-28	7.30E-28	-27.145	-27.137	0.008
Mg		5.23E-03					
	Mg+2		4.84E-03	1.94E-03	-2.315	-2.713	-0.399
	Mg(H2Tartrate)		3.91E-04	3.98E-04	-3.408	-3.4	0.008
	MgOH+		3.03E-07	2.41E-07	-6.519	-6.619	-0.1
	MgH(H2Tartrate)+		1.52E-08	1.21E-08	-7.818	-7.918	-0.1
O(0)		1.62E-39					
	O2		8.08E-40	8.23E-40	-39.093	-39.085	0.008
Si		1.06E-03					
	H4SiO4		1.04E-03	1.06E-03	-2.984	-2.976	0.008
	H3SiO4-		1.85E-05	1.47E-05	-4.732	-4.831	-0.1
	H5Si2O7-		1.12E-06	8.88E-07	-5.952	-6.051	-0.1
	H4Si2O7-2		2.80E-09	1.12E-09	-8.553	-8.951	-0.399
	H2SiO4-2		1.41E-10	5.63E-11	-9.851	-10.249	-0.399
	H5Si3O10-3		2.95E-12	3.74E-13	-11.53	-12.427	-0.897
	H5Si4O12-3		3.12E-13	3.96E-14	-12.506	-13.402	-0.897
	H3Si3O9-3		2.34E-13	2.97E-14	-12.63	-13.527	-0.897
	H4Si4O12-4		2.47E-15	6.28E-17	-14.608	-16.202	-1.594
Tartrate		2.29E-02					
	H2Tartrate-2		1.87E-02	7.47E-03	-1.728	-2.127	-0.399
	Ca(H2Tartrate)		1.63E-03	1.66E-03	-2.788	-2.78	0.008
	Al(Tartrate)(HTartrate)2-7		6.89E-04	9.02E-09	-3.162	-8.045	-4.883
	Mg(H2Tartrate)		3.91E-04	3.98E-04	-3.408	-3.4	0.008
	Al(Tartrate)2(HTartrate)-8		2.77E-05	1.16E-11	-4.557	-10.935	-6.378
	Fe(H2Tartrate)		2.46E-05	2.51E-05	-4.609	-4.601	0.008
	Al(Tartrate)(HTartrate)-4		7.19E-06	1.83E-07	-5.143	-6.738	-1.594
	HTartrate-3		2.19E-06	2.77E-07	-5.66	-6.557	-0.897
	H(H2Tartrate)-		8.82E-07	7.01E-07	-6.054	-6.154	-0.1

	CaH(H2Tartrate)+	4.29E-08	3.41E-08	-7.368	-7.468	-0.1
	Al(Tartrate)3-9	2.74E-08	2.32E-16	-7.563	-15.635	-8.072
	Al(Tartrate)-	1.82E-08	1.44E-08	-7.741	-7.841	-0.1
	MgH(H2Tartrate)+	1.52E-08	1.21E-08	-7.818	-7.918	-0.1
	Al(HTartrate)(H2Tartrate)-2	2.14E-10	8.56E-11	-9.669	-10.068	-0.399
	Tartrate-4	1.17E-10	2.97E-12	-9.933	-11.527	-1.594
	Al(HTartrate)	2.00E-11	2.04E-11	-10.699	-10.691	0.008
	H2(H2Tartrate)	4.61E-12	4.70E-12	-11.336	-11.328	0.008
	Al2(Tartrate)2-2	3.22E-14	1.28E-14	-13.493	-13.891	-0.399
	Al(H2Tartrate)2-	5.65E-15	4.49E-15	-14.248	-14.348	-0.1
	Al2(HTartrate)(Tartrate)-	1.09E-16	8.68E-17	-15.962	-16.061	-0.1
	Al(H2Tartrate)+	1.55E-17	1.23E-17	-16.811	-16.911	-0.1
	Al2(HTartrate)2	1.38E-20	1.41E-20	-19.859	-19.851	0.008
	Al2(HTartrate)(H2Tartrate)+	7.22E-26	5.74E-26	-25.142	-25.241	-0.1
Ti(4)	5.87E-05					
	Ti(OH)4	5.87E-05	5.98E-05	-4.231	-4.223	0.008

Saturation indices

Phase	SI**	log	IAP	log
afwillite	-12.63	34.46	47.1	Ca3Si2O4(OH)6
akermanite	-11.49	34.28	45.77	Ca2MgSi2O7
Al(OH)3(amorph.)	-4.74	6.26	11	Al(OH)3
amesite-14a	4.75	36.11	31.36	Mg2Al2SiO5(OH)4
amrph.silica	-0.25	-2.98	-2.72	SiO2
andalusite	-7.46	9.54	17	Al2SiO5
anorthite	-7.61	20.03	27.64	CaAl2(SiO4)2
anthophyllite	0.27	69.2	68.93	Mg7Si8O22(OH)2
antigorite	86.28	268.29	182	Mg24Si17O42.5(OH)31
beidellit-ca	-0.82	5.88	6.7	Ca0.165Al2.33Si3.67O10(OH)2
beidellit-mg	-0.84	5.85	6.69	Mg0.165Al2.33Si3.67O10(OH)2
boehmite	-3.56	6.26	9.81	AlO2H
Ca(H2Tartrate):4H2O	1.32	-4.66	-5.98	Ca(H2Tartrate):4H2O
Ca(OH)2	-9.57	13.47	23.04	Ca(OH)2
ca-al	-14.1	23.01	37.11	CaAl2SiO6
ca-p	-1.95	18.25	20.2	CaAl2Si2.6O12.4H6.4
Ca2Al2O13H16	-19.18	39.45	58.63	Ca2Al2O13H16
Ca2Al2SiO15H16	-12.57	36.48	49.05	Ca2Al2SiO15H16
Ca2SiO4	-13.94	23.97	37.91	Ca2SiO4
Ca3Al2O12H12	-26.45	52.93	79.38	Ca3Al2O12H12
Ca3Al2Si0.5O12H10	-22.03	51.44	73.47	Ca3Al2Si0.5O12H10
Ca3SiO5	-37.35	37.44	74.79	Ca3SiO5
Ca4Al2O20H26	-35.9	66.4	102.3	Ca4Al2O20H26

CaO	-19.5	13.47	32.97	CaO
chabazite	0.5	14.08	13.58	CaAl2Si4O12:6H2O
chalcedony	0.58	-2.98	-3.55	SiO2
chamosite-7a	0.42	32.11	31.69	Fe2Al2SiO5(OH)4
chrysotile	1.9	33.91	32.01	Mg3Si2O5(OH)4
chs(0.8)	-3.23	7.8	11.03	Ca0.8SiO5H4.4
clinochl-14a	1.45	70.02	68.57	Mg5Al2Si3O10(OH)8
clinochl-7a	-1.92	70.02	71.94	Mg5Al2Si3O10(OH)8
clinozoisite	-7.92	36.79	44.71	Ca2Al3Si3O12(OH)
cordier.anh	-18.35	36.72	55.07	Mg2Al4Si5O18
cordier.hydr	-15.84	36.72	52.55	Mg2Al4Si5O18:H2O
corundum	-9.33	12.51	21.84	Al2O3
crist.beta_amorph	0.04	-2.98	-3.02	SiO2
cristobalite	0.49	-2.98	-3.47	SiO2
csh(1.1)	-4.82	11.84	16.66	Ca1.1SiO7H7.8
csh(1.8)	-11.14	21.27	32.41	Ca1.8SiO9H10.4
daphnite-14a	8.7	60.01	51.31	Fe5Al2Si3O10(OH)8
daphnite-7a	5.31	60.01	54.71	Fe5Al2Si3O10(OH)8
diaspore	-2.69	6.26	8.95	AlHO2
diopside	-0.47	20.81	21.28	CaMgSi2O6
enstatite	-1.34	10.31	11.65	MgSiO3
epistilbite	2.63	8.13	5.5	CaAl2Si6O16:5H2O
fayalite	0.28	19.6	19.32	Fe2SiO4
Fe(H2Tartrate):3H2O	1.39	-6.84	-8.23	Fe(H2Tartrate):3H2O
Fe(OH)2	-2.72	11.29	14.01	Fe(OH)2
FeO	-2.37	11.29	13.66	FeO
ferrosilite	0.78	8.31	7.53	FeSiO3
forsterite	-4.96	23.6	28.55	Mg2SiO4
foshagite	-14.45	44.96	59.41	Ca4Si3O9(OH)2:0.5H2O
gehlenite	-21.05	36.48	57.53	Ca2Al2SiO7
gibbsite	-1.67	6.26	7.93	Al(OH)3
greenalite	5.03	27.91	22.87	Fe3Si2O5(OH)4
grossular	-10.11	44	54.11	Ca3Al2(SiO4)3
gyrolite	-2.52	18.02	20.54	Ca2Si3O7(OH)2:1.5H2O
H2(g)	-24	-27.14	-3.14	H2
halloy	-3.92	6.56	10.48	Al2Si2O9H4
hedenbergite	-1.03	18.81	19.84	CaFe(SiO3)2
hercynite	-3.88	23.8	27.68	FeAl2O4
heulandite-ca	3.69	5.15	1.47	CaAl2Si7O18:6H2O
hillebrandite	-8.59	23.97	32.55	Ca2SiO3(OH)2:0.167H2O

BFS_CA_8

temp	22
pH	8

pe	4		
density	0.997		
Si	55.64179		
Al	633.1905	umol/kgw	
Fe(2)	29.90714	umol/kgw	
Ca	695.5224		
Mg	125.194		
Ti(4)	51.37948		
Citrate	0.013869	mol/kgw	
water	1	#	kg
Elements	Molality		
Al	6.33E-04		
Ca	1.74E-02		
Citrate	1.39E-02		
Fe(2)	2.99E-05		
Mg	5.15E-03		
Si	9.26E-04		
Ti(4)	1.07E-03		

Distribution of species

		Species	Molality	Activity	log Molality	log Activity	log Gamma	mole V cm³/mol
		OH-	9.64E-07	7.95E-07	-6.016	-6.1	-0.084	0
		H+	1.21E-08	1.00E-08	-7.916	-8	-0.084	0
		H2O	5.55E+01	1.00E+00	1.744	0	0	18.06
Al	6.33E-04							
		Al(Citrate)2-5	3.72E-04	3.00E-06	-3.43	-5.523	-2.093	0
		Al(OH)(Citrate)-2	2.44E-04	1.13E-04	-3.612	-3.947	-0.335	0
		Al(Citrate)(HCitrate)-4	9.69E-06	4.43E-07	-5.014	-6.353	-1.34	0
		Al(Citrate)-	2.17E-06	1.79E-06	-5.663	-5.747	-0.084	0
		Al3(OH)4(Citrate)3-7	1.45E-06	1.14E-10	-5.839	-9.942	-4.103	0
		Al(OH)4-	7.18E-07	5.92E-07	-6.144	-6.228	-0.084	0
		Al3(OH)4(HCitrate)3-4	1.37E-07	6.28E-09	-6.863	-8.202	-1.34	0
		Al(HCitrate)2-3	3.16E-08	5.58E-09	-7.5	-8.253	-0.754	0
		Al2(OH)2(HCitrate)2-2	2.17E-08	1.01E-08	-7.663	-7.998	-0.335	0
		Al(OH)3	5.80E-09	5.86E-09	-8.237	-8.232	0.005	0
		Al(OH)2+	5.61E-10	4.62E-10	-9.251	-9.335	-0.084	0
		Al(HCitrate)	1.00E-10	1.01E-10	-9.999	-9.994	0.005	0
		AlOH+2	2.01E-12	9.27E-13	-11.698	-12.033	-0.335	0
		Al+3	5.24E-15	9.24E-16	-14.281	-15.034	-0.754	0
		AlH(HCitrate)+	4.86E-16	4.01E-16	-15.313	-15.397	-0.084	0
Ca	1.74E-02							
		Ca+2	9.50E-03	4.39E-03	-2.022	-2.357	-0.335	0

	Ca(HCitrate)-	7.85E-03	6.47E-03	-2.105	-2.189	-0.084	0
	CaH(HCitrate)	1.08E-06	1.10E-06	-5.965	-5.96	0.005	0
	Ca(OH)+	1.04E-07	8.60E-08	-6.982	-7.066	-0.084	0
	CaH2(HCitrate)+	5.92E-17	4.88E-17	-16.227	-16.311	-0.084	0
Citrate	1.39E-02						
	Ca(HCitrate)-	7.85E-03	6.47E-03	-2.105	-2.189	-0.084	0
	HCitrate-3	2.78E-03	4.90E-04	-2.556	-3.31	-0.754	0
	Mg(HCitrate)-	2.19E-03	1.81E-03	-2.66	-2.743	-0.084	0
	Al(Citrate)2-5	3.72E-04	3.00E-06	-3.43	-5.523	-2.093	0
	Al(OH)(Citrate)-2	2.44E-04	1.13E-04	-3.612	-3.947	-0.335	0
	Fe(HCitrate)-	2.50E-05	2.07E-05	-4.601	-4.685	-0.084	0
	Al(Citrate)(HCitrate)-4	9.69E-06	4.43E-07	-5.014	-6.353	-1.34	0
	H(HCitrate)-2	4.59E-06	2.12E-06	-5.338	-5.673	-0.335	0
	H4SiO4(HCitrate)-3	3.28E-06	5.78E-07	-5.484	-6.238	-0.754	0
	Al(Citrate)-	2.17E-06	1.79E-06	-5.663	-5.747	-0.084	0
	Al3(OH)4(Citrate)3-7	1.45E-06	1.14E-10	-5.839	-9.942	-4.103	0
	CaH(HCitrate)	1.08E-06	1.10E-06	-5.965	-5.96	0.005	0
	Fe2(Citrate)2-4	5.88E-07	2.69E-08	-6.23	-7.57	-1.34	0
	MgH(HCitrate)	1.81E-07	1.83E-07	-6.741	-6.737	0.005	0
	Al3(OH)4(HCitrate)3-4	1.37E-07	6.28E-09	-6.863	-8.202	-1.34	0
	Al(HCitrate)2-3	3.16E-08	5.58E-09	-7.5	-8.253	-0.754	0
	Al2(OH)2(HCitrate)2-2	2.17E-08	1.01E-08	-7.663	-7.998	-0.335	0
	FeH(HCitrate)2-3	1.22E-08	2.15E-09	-7.915	-8.668	-0.754	0
	FeH(HCitrate)	2.80E-09	2.83E-09	-8.553	-8.549	0.005	0
	Al(HCitrate)	1.00E-10	1.01E-10	-9.999	-9.994	0.005	0
	H2(HCitrate)-	1.35E-15	1.11E-15	-14.87	-14.954	-0.084	0
	AlH(HCitrate)+	4.86E-16	4.01E-16	-15.313	-15.397	-0.084	0
	CaH2(HCitrate)+	5.92E-17	4.88E-17	-16.227	-16.311	-0.084	0
	MgH2(HCitrate)+	9.25E-18	7.63E-18	-17.034	-17.118	-0.084	0
	FeH2(HCitrate)+	2.85E-20	2.35E-20	-19.546	-19.63	-0.084	0
	H3(HCitrate)	3.92E-25	3.97E-25	-24.406	-24.401	0.005	0
Fe(2)	2.99E-05						
	Fe(HCitrate)-	2.50E-05	2.07E-05	-4.601	-4.685	-0.084	0
	Fe+2	3.63E-06	1.68E-06	-5.441	-5.775	-0.335	0
	Fe2(Citrate)2-4	5.88E-07	2.69E-08	-6.23	-7.57	-1.34	0
	Fe(OH)+	5.13E-08	4.23E-08	-7.29	-7.374	-0.084	0
	FeH(HCitrate)2-3	1.22E-08	2.15E-09	-7.915	-8.668	-0.754	0
	FeH(HCitrate)	2.80E-09	2.83E-09	-8.553	-8.549	0.005	0
	Fe(OH)2	2.55E-11	2.58E-11	-10.594	-10.589	0.005	0
	Fe(OH)3-	1.21E-13	9.96E-14	-12.918	-13.002	-0.084	0
	Fe(OH)4-2	3.62E-20	1.67E-20	-19.441	-19.776	-0.335	0
	FeH2(HCitrate)+	2.85E-20	2.35E-20	-19.546	-19.63	-0.084	0
H(0)	1.44E-27						

	H2	7.22E-28	7.30E-28	-27.142	-27.137	0.005	0
Mg	5.15E-03						
	Mg+2	2.96E-03	1.37E-03	-2.529	-2.864	-0.335	0
	Mg(HCitrate)-	2.19E-03	1.81E-03	-2.66	-2.743	-0.084	0
	MgOH+	2.07E-07	1.70E-07	-6.685	-6.769	-0.084	0
	MgH(HCitrate)	1.81E-07	1.83E-07	-6.741	-6.737	0.005	0
	MgH2(HCitrate)+	9.25E-18	7.63E-18	-17.034	-17.118	-0.084	0
O(0)	1.63E-39						
	O2	8.14E-40	8.23E-40	-39.089	-39.085	0.005	0
Si	9.26E-04						
	H4SiO4	9.06E-04	9.16E-04	-3.043	-3.038	0.005	0
	H3SiO4-	1.55E-05	1.28E-05	-4.81	-4.894	-0.084	0
	H4SiO4(HCitrate)-3	3.28E-06	5.78E-07	-5.484	-6.238	-0.754	0
	H5Si2O7-	8.08E-07	6.66E-07	-6.093	-6.176	-0.084	0
	H4Si2O7-2	1.81E-09	8.39E-10	-8.741	-9.076	-0.335	0
	H2SiO4-2	1.06E-10	4.88E-11	-9.977	-10.312	-0.335	0
	H5Si3O10-3	1.38E-12	2.43E-13	-11.861	-12.614	-0.754	0
	H5Si4O12-3	1.26E-13	2.23E-14	-12.899	-13.652	-0.754	0
	H3Si3O9-3	1.10E-13	1.93E-14	-12.961	-13.714	-0.754	0
	H4Si4O12-4	7.72E-16	3.53E-17	-15.112	-16.452	-1.34	0
Ti(4)	1.07E-03						
	Ti(OH)4	1.07E-03	1.09E-03	-2.969	-2.965	0.005	0

Saturation indices

Phase	SI**	log	IAP	log
afwillite	-12.25	34.85	47.1	Ca3Si2O4(OH)6
akermanite	-11.43	34.35	45.77	Ca2MgSi2O7
Al(OH)3(amorph.)	-2.03	8.97	11	Al(OH)3
amesite-14a	9.81	41.17	31.36	Mg2Al2SiO5(OH)4
amrph.silica	-0.32	-3.04	-2.72	SiO2
andalusite	-2.1	14.89	17	Al2SiO5
anorthite	-2.14	25.5	27.64	CaAl2(SiO4)2
anthophyllite	-1.28	67.65	68.93	Mg7Si8O22(OH)2
antigorite	81.62	263.62	182	Mg24Si17O42.5(OH)31
beidellit-ca	5.29	11.99	6.7	Ca0.165Al2.33Si3.67O10(OH)2
beidellit-mg	5.22	11.91	6.69	Mg0.165Al2.33Si3.67O10(OH)2
boehmite	-0.85	8.97	9.81	AlO2H
Ca(OH)2	-9.4	13.64	23.04	Ca(OH)2
ca-al	-8.58	28.54	37.11	CaAl2SiO6
ca-p	3.47	23.67	20.2	CaAl2Si2.6O12.4H6.4
Ca2Al2O13H16	-13.42	45.21	58.63	Ca2Al2O13H16
Ca2Al2SiO15H16	-6.87	42.18	49.05	Ca2Al2SiO15H16

Ca2SiO4	-13.66	24.25	37.91	Ca2SiO4
Ca3(HCitrate)2	3.34	-13.69	-17.03	Ca3(HCitrate)2
Ca3(HCitrate)2:4H2O	-1.85	-13.69	-11.84	Ca3(HCitrate)2:4H2O
Ca3Al2O12H12	-20.52	58.86	79.38	Ca3Al2O12H12
Ca3Al2Si0.5O12H10	-16.13	57.34	73.47	Ca3Al2Si0.5O12H10
Ca3SiO5	-36.9	37.89	74.79	Ca3SiO5
Ca4Al2O20H26	-29.8	72.5	102.3	Ca4Al2O20H26
CaH(HCitrate)	-2.28	-13.67	-11.39	CaH(HCitrate)
CaO	-19.33	13.64	32.97	CaO
chabazite	5.84	19.42	13.58	CaAl2Si4O12:6H2O
chalcedony	0.52	-3.04	-3.55	SiO2
chamosite-7a	3.65	35.34	31.69	Fe2Al2SiO5(OH)4
chrysotile	1.32	33.33	32.01	Mg3Si2O5(OH)4
chs(0.8)	-3.15	7.88	11.03	Ca0.8SiO5H4.4
clinochl-14a	5.93	74.5	68.57	Mg5Al2Si3O10(OH)8
clinochl-7a	2.56	74.5	71.94	Mg5Al2Si3O10(OH)8
clinozoisite	0.36	45.07	44.71	Ca2Al3Si3O12(OH)
cordier.anh	-8.12	46.95	55.07	Mg2Al4Si5O18
cordier.hydr	-5.61	46.94	52.55	Mg2Al4Si5O18:H2O
corundum	-3.91	17.93	21.84	Al2O3
crist.beta_amorph	-0.02	-3.04	-3.02	SiO2
cristobalite	0.43	-3.04	-3.47	SiO2
csh(1.1)	-4.69	11.97	16.66	Ca1.1SiO7H7.8
csh(1.8)	-10.89	21.52	32.41	Ca1.8SiO9H10.4
daphnite-14a	8.63	59.94	51.31	Fe5Al2Si3O10(OH)8
daphnite-7a	5.23	59.94	54.71	Fe5Al2Si3O10(OH)8
diaspore	0.02	8.97	8.95	AlHO2
diopside	-0.57	20.7	21.28	CaMgSi2O6
enstatite	-1.55	10.1	11.65	MgSiO3
epistilbite	7.84	13.35	5.5	CaAl2Si6O16:5H2O
fayalite	-1.91	17.41	19.32	Fe2SiO4
Fe(OH)2	-3.79	10.22	14.01	Fe(OH)2
FeO	-3.43	10.22	13.66	FeO
ferrosilite	-0.35	7.19	7.53	FeSiO3
forsterite	-5.32	23.23	28.55	Mg2SiO4
foshagite	-13.95	45.46	59.41	Ca4Si3O9(OH)2:0.5H2O
gehlenite	-15.35	42.18	57.53	Ca2Al2SiO7
gibbsite	1.04	8.97	7.93	Al(OH)3
greenalite	1.72	24.6	22.87	Fe3Si2O5(OH)4
grossular	-4.36	49.75	54.11	Ca3Al2(SiO4)3
gyrolite	-2.37	18.17	20.54	Ca2Si3O7(OH)2:1.5H2O
H2(g)	-24	-27.14	-3.14	H2
halloy	1.37	11.85	10.48	Al2Si2O9H4
hedenbergite	-2.05	17.79	19.84	CaFe(SiO3)2

hercynite	0.48	28.16	27.68	FeAl2O4
heulandite-ca	8.84	10.31	1.47	CaAl2Si7O18:6H2O
hillebrandite	-8.31	24.25	32.55	Ca2SiO3(OH)2:0.167H2O
hydrogarnet	-21.94	58.86	80.8	Ca3Al2O6:6H2O
hydrotalcite	-4.87	70.47	75.34	Mg4Al2O17H20
jennite	-42.05	104.55	146.6	Ca9Si6O32H22
kaolinite	2.76	-34.53	-37.29	Al2Si2O5(OH)4
katoite	-13.07	55.82	68.89	Ca3Al2SiO12H8
kyanite	-1.83	14.89	16.72	Al2SiO5
larnite	-15.17	24.25	39.42	Ca2SiO4
laumontite	4.02	19.42	15.4	CaAl2Si4O12:4H2O
lawsonite	2.31	25.5	23.19	CaAl2Si2O7(OH)2:H2O
margarite	0.31	43.43	43.12	CaAl4Si2O10(OH)2
merwinite	-21.1	47.99	69.08	MgCa3(SiO4)2
Mg(OH)2	-3.92	13.14	17.05	Mg(OH)2
minnesotaite	4.38	18.52	14.15	Fe3Si4O10(OH)2
monticellite	-6.39	23.74	30.13	CaMgSiO4
montmor-ca	6.05	9.41	3.35	Ca0.165Mg0.33Al1.67Si4O10(OH)2
montmor-mg	6.05	9.32	3.28	Mg0.495Al1.67Si4O10(OH)2
O2(g)	-36.14	-39.08	-2.95	O2
okenite	2.49	7.57	5.07	CaSi2O4(OH)2:H2O
phillipsite-ca	6.78	16.38	9.6	CaAl2Si5O14:5H2O
prehnite	2.19	36.1	33.91	Ca2Al2Si3O10(OH)2
pseudo	-3.59	10.6	14.2	CaSiO3
pyrophyllite	4.43	5.78	1.35	Al2Si4O10(OH)2
quartz	1.01	-3.04	-4.05	SiO2
rankinite	-17.72	34.85	52.57	Ca3Si2O7
ripidolit-14a	8.42	68.67	60.25	Mg3Fe2Al2Si3O10(OH)8
ripidolit-7a	5.03	68.67	63.64	Mg3Fe2Al2Si3O10(OH)8
saponite-ca	6.15	33.47	27.32	Ca0.165Mg3Al0.33Si3.67O10(OH)2
saponite-h	4.89	31.22	26.33	H0.33Mg3Al0.33Si3.67O10(OH)2
saponite-mg	6.08	33.39	27.3	Mg3.165Al0.33Si3.67O10(OH)2
scolecite	7.32	22.46	15.14	CaAl2Si3O10:3H2O
sepiolite	1.04	-39.24	-40.28	Mg2Si3O7.5OH:3H2O
sillimantite	-2.47	14.89	17.36	Al2SiO5
spinel	-8.01	31.07	39.07	Al2MgO4
stratlingite	-7.15	42.18	49.33	Ca2Al2Si1O15H16
talc	5.29	27.26	21.97	Mg3Si4O10(OH)2
tobermorite(11A)	-16.85	49.99	66.83	Ca5Si6H11O22.5
tobermorite(14A)	-14.34	49.98	64.32	Ca5Si6H21O27.5
tobermorite(9A)	-19.8	49.99	69.79	Ca5Si6H6O20
tremolite	5.99	68.66	62.67	Ca2Mg5Si8O22(OH)2
wairakite	-0.5	19.42	19.92	CaAl2Si4O12:2H2O
wollastonite	-3.19	10.6	13.79	CaSiO3

xonotlite	-3.82	63.63	67.45	Ca6Si6O17(OH)2
yugawaralite	2.49	6.67	4.18	Ca0.5AlSi3O8:2H2O
zoisite	0.23	45.07	44.84	Ca2Al3(SiO4)3OH

BFS_TA_8

temp	22		
pH	8		
pe	4		
density	0.997		
Si	63.61141		
Al	723.88	umol/kgw	
Fe(2)	4.11		
Ca	362.95		
Mg	127.222		
Ti(4)	58.73861	umol/kgw	
Tartrate	0.0229	mol/kgw	
water	1	#	kg
Elements	Molality		
Al	7.24E-04		
Ca	9.06E-03		
Fe(2)	7.36E-05		
Mg	5.23E-03		
Si	1.06E-03		
Tartrate	2.29E-02		
Ti(4)	5.87E-05		

Distribution of species

	Species	Molality	Activity	log Molality	log Activity	log Gamma	mole V cm³/mol
	OH-	1.00E-06	7.95E-07	-6	-6.1	-0.1	0
	H+	1.26E-08	1.00E-08	-7.9	-8	-0.1	0
	H2O	5.55E+01	9.99E-01	1.744	0	0	18.06
Al 7.24E-04							
	Al(Tartrate)(HTartrate)2-7	6.89E-04	9.02E-09	-3.162	-8.045	-4.883	0
	Al(Tartrate)2(HTartrate)-8	2.77E-05	1.16E-11	-4.557	-10.935	-6.378	0
	Al(Tartrate)(HTartrate)-4	7.19E-06	1.83E-07	-5.143	-6.738	-1.594	0
	Al(Tartrate)3-9	2.74E-08	2.32E-16	-7.563	-15.635	-8.072	0
	Al(Tartrate)-	1.82E-08	1.44E-08	-7.741	-7.841	-0.1	0
	Al(OH)4-	1.45E-09	1.16E-09	-8.838	-8.937	-0.1	0
	Al(HTartrate)(H2Tartrate)-2	2.14E-10	8.56E-11	-9.669	-10.068	-0.399	0
	Al(HTartrate)	2.00E-11	2.04E-11	-10.699	-10.691	0.008	0
	Al(OH)3	1.12E-11	1.14E-11	-10.95	-10.942	0.008	0
	Al(OH)2+	1.14E-12	9.02E-13	-11.945	-12.045	-0.1	0
	Al2(Tartrate)2-2	3.22E-14	1.28E-14	-13.493	-13.891	-0.399	0
	Al(H2Tartrate)2-	5.65E-15	4.49E-15	-14.248	-14.348	-0.1	0

	AlOH+2	4.53E-15	1.81E-15	-14.344	-14.742	-0.399	0
	Al2(HTartrate)(Tartrate)-	1.09E-16	8.68E-17	-15.962	-16.061	-0.1	0
	Al(H2Tartrate)+	1.55E-17	1.23E-17	-16.811	-16.911	-0.1	0
	Al+3	1.42E-17	1.80E-18	-16.847	-17.744	-0.897	0
	Al2(HTartrate)2	1.38E-20	1.41E-20	-19.859	-19.851	0.008	0
	Al2(HTartrate)(H2Tartrate)+	7.22E-26	5.74E-26	-25.142	-25.241	-0.1	0
Ca	9.06E-03						
	Ca+2	7.43E-03	2.97E-03	-2.129	-2.528	-0.399	0
	Ca(H2Tartrate)	1.63E-03	1.66E-03	-2.788	-2.78	0.008	0
	Ca(OH)+	7.30E-08	5.80E-08	-7.137	-7.236	-0.1	0
	CaH(H2Tartrate)+	4.29E-08	3.41E-08	-7.368	-7.468	-0.1	0
Fe(2)	7.36E-05						
	Fe+2	4.84E-05	1.93E-05	-4.315	-4.714	-0.399	0
	Fe(H2Tartrate)	2.46E-05	2.51E-05	-4.609	-4.601	0.008	0
	Fe(OH)+	6.13E-07	4.87E-07	-6.213	-6.313	-0.1	0
	Fe(OH)2	2.91E-10	2.97E-10	-9.536	-9.528	0.008	0
	Fe(OH)3-	1.44E-12	1.15E-12	-11.841	-11.94	-0.1	0
	Fe(OH)4-2	4.83E-19	1.93E-19	-18.316	-18.715	-0.399	0
H(0)	1.43E-27						
	H2	7.16E-28	7.30E-28	-27.145	-27.137	0.008	0
Mg	5.23E-03						
	Mg+2	4.84E-03	1.94E-03	-2.315	-2.713	-0.399	0
	Mg(H2Tartrate)	3.91E-04	3.98E-04	-3.408	-3.4	0.008	0
	MgOH+	3.03E-07	2.41E-07	-6.519	-6.619	-0.1	0
	MgH(H2Tartrate)+	1.52E-08	1.21E-08	-7.818	-7.918	-0.1	0
O(0)	1.62E-39						
	O2	8.08E-40	8.23E-40	-39.093	-39.085	0.008	0
Si	1.06E-03						
	H4SiO4	1.04E-03	1.06E-03	-2.984	-2.976	0.008	0
	H3SiO4-	1.85E-05	1.47E-05	-4.732	-4.831	-0.1	0
	H5Si2O7-	1.12E-06	8.88E-07	-5.952	-6.051	-0.1	0
	H4Si2O7-2	2.80E-09	1.12E-09	-8.553	-8.951	-0.399	0
	H2SiO4-2	1.41E-10	5.63E-11	-9.851	-10.249	-0.399	0
	H5Si3O10-3	2.95E-12	3.74E-13	-11.53	-12.427	-0.897	0
	H5Si4O12-3	3.12E-13	3.96E-14	-12.506	-13.402	-0.897	0
	H3Si3O9-3	2.34E-13	2.97E-14	-12.63	-13.527	-0.897	0
	H4Si4O12-4	2.47E-15	6.28E-17	-14.608	-16.202	-1.594	0
Tartrate	2.29E-02						
	H2Tartrate-2	1.87E-02	7.47E-03	-1.728	-2.127	-0.399	0
	Ca(H2Tartrate)	1.63E-03	1.66E-03	-2.788	-2.78	0.008	0
	Al(Tartrate)(HTartrate)2-7	6.89E-04	9.02E-09	-3.162	-8.045	-4.883	0
	Mg(H2Tartrate)	3.91E-04	3.98E-04	-3.408	-3.4	0.008	0
	Al(Tartrate)2(HTartrate)-8	2.77E-05	1.16E-11	-4.557	-10.935	-6.378	0
	Fe(H2Tartrate)	2.46E-05	2.51E-05	-4.609	-4.601	0.008	0

	Al(Tartrate)(HTartrate)-4	7.19E-06	1.83E-07	-5.143	-6.738	-1.594	0
	HTartrate-3	2.19E-06	2.77E-07	-5.66	-6.557	-0.897	0
	H(H2Tartrate)-	8.82E-07	7.01E-07	-6.054	-6.154	-0.1	0
	CaH(H2Tartrate)+	4.29E-08	3.41E-08	-7.368	-7.468	-0.1	0
	Al(Tartrate)3-9	2.74E-08	2.32E-16	-7.563	-15.635	-8.072	0
	Al(Tartrate)-	1.82E-08	1.44E-08	-7.741	-7.841	-0.1	0
	MgH(H2Tartrate)+	1.52E-08	1.21E-08	-7.818	-7.918	-0.1	0
	Al(HTartrate)(H2Tartrate)-2	2.14E-10	8.56E-11	-9.669	-10.068	-0.399	0
	Tartrate-4	1.17E-10	2.97E-12	-9.933	-11.527	-1.594	0
	Al(HTartrate)	2.00E-11	2.04E-11	-10.699	-10.691	0.008	0
	H2(H2Tartrate)	4.61E-12	4.70E-12	-11.336	-11.328	0.008	0
	Al2(Tartrate)2-2	3.22E-14	1.28E-14	-13.493	-13.891	-0.399	0
	Al(H2Tartrate)2-	5.65E-15	4.49E-15	-14.248	-14.348	-0.1	0
	Al2(HTartrate)(Tartrate)-	1.09E-16	8.68E-17	-15.962	-16.061	-0.1	0
	Al(H2Tartrate)+	1.55E-17	1.23E-17	-16.811	-16.911	-0.1	0
	Al2(HTartrate)2	1.38E-20	1.41E-20	-19.859	-19.851	0.008	0
	Al2(HTartrate)(H2Tartrate)+	7.22E-26	5.74E-26	-25.142	-25.241	-0.1	0
Ti(4)	5.87E-05						
	Ti(OH)4	5.87E-05	5.98E-05	-4.231	-4.223	0.008	0

Saturation indices

Phase	SI**	log	IAP	log
afwillite	-12.63	34.46	47.1	Ca3Si2O4(OH)6
akermanite	-11.49	34.28	45.77	Ca2MgSi2O7
Al(OH)3(amorph.)	-4.74	6.26	11	Al(OH)3
amesite-14a	4.75	36.11	31.36	Mg2Al2SiO5(OH)4
amrph.silica	-0.25	-2.98	-2.72	SiO2
andalusite	-7.46	9.54	17	Al2SiO5
anorthite	-7.61	20.03	27.64	CaAl2(SiO4)2
anthophyllite	0.27	69.2	68.93	Mg7Si8O22(OH)2
antigorite	86.28	268.29	182	Mg24Si17O42.5(OH)31
beidellit-ca	-0.82	5.88	6.7	Ca0.165Al2.33Si3.67O10(OH)2
beidellit-mg	-0.84	5.85	6.69	Mg0.165Al2.33Si3.67O10(OH)2
boehmite	-3.56	6.26	9.81	AlO2H
Ca(H2Tartrate):4H2O	1.32	-4.66	-5.98	Ca(H2Tartrate):4H2O
Ca(OH)2	-9.57	13.47	23.04	Ca(OH)2
ca-al	-14.1	23.01	37.11	CaAl2SiO6
ca-p	-1.95	18.25	20.2	CaAl2Si2.6O12.4H6.4
Ca2Al2O13H16	-19.18	39.45	58.63	Ca2Al2O13H16
Ca2Al2SiO15H16	-12.57	36.48	49.05	Ca2Al2SiO15H16
Ca2SiO4	-13.94	23.97	37.91	Ca2SiO4
Ca3Al2O12H12	-26.45	52.93	79.38	Ca3Al2O12H12

Ca3Al2Si0.5O12H10	-22.03	51.44	73.47	Ca3Al2Si0.5O12H10
Ca3SiO5	-37.35	37.44	74.79	Ca3SiO5
Ca4Al2O20H26	-35.9	66.4	102.3	Ca4Al2O20H26
CaO	-19.5	13.47	32.97	CaO
chabazite	0.5	14.08	13.58	CaAl2Si4O12:6H2O
chalcedony	0.58	-2.98	-3.55	SiO2
chamosite-7a	0.42	32.11	31.69	Fe2Al2SiO5(OH)4
chrysotile	1.9	33.91	32.01	Mg3Si2O5(OH)4
chs(0.8)	-3.23	7.8	11.03	Ca0.8SiO5H4.4
clinochl-14a	1.45	70.02	68.57	Mg5Al2Si3O10(OH)8
clinochl-7a	-1.92	70.02	71.94	Mg5Al2Si3O10(OH)8
clinozoisite	-7.92	36.79	44.71	Ca2Al3Si3O12(OH)
cordier.anh	-18.35	36.72	55.07	Mg2Al4Si5O18
cordier.hydr	-15.84	36.72	52.55	Mg2Al4Si5O18:H2O
corundum	-9.33	12.51	21.84	Al2O3
crist.beta_amorph	0.04	-2.98	-3.02	SiO2
cristobalite	0.49	-2.98	-3.47	SiO2
csh(1.1)	-4.82	11.84	16.66	Ca1.1SiO7H7.8
csh(1.8)	-11.14	21.27	32.41	Ca1.8SiO9H10.4
daphnite-14a	8.7	60.01	51.31	Fe5Al2Si3O10(OH)8
daphnite-7a	5.31	60.01	54.71	Fe5Al2Si3O10(OH)8
diaspore	-2.69	6.26	8.95	AlHO2
diopside	-0.47	20.81	21.28	CaMgSi2O6
enstatite	-1.34	10.31	11.65	MgSiO3
epistilbite	2.63	8.13	5.5	CaAl2Si6O16:5H2O
fayalite	0.28	19.6	19.32	Fe2SiO4
Fe(H2Tartrate):3H2O	1.39	-6.84	-8.23	Fe(H2Tartrate):3H2O
Fe(OH)2	-2.72	11.29	14.01	Fe(OH)2
FeO	-2.37	11.29	13.66	FeO
ferrosilite	0.78	8.31	7.53	FeSiO3
forsterite	-4.96	23.6	28.55	Mg2SiO4
foshagite	-14.45	44.96	59.41	Ca4Si3O9(OH)2:0.5H2O
gehlenite	-21.05	36.48	57.53	Ca2Al2SiO7
gibbsite	-1.67	6.26	7.93	Al(OH)3
greenalite	5.03	27.91	22.87	Fe3Si2O5(OH)4
grossular	-10.11	44	54.11	Ca3Al2(SiO4)3
gyrolite	-2.52	18.02	20.54	Ca2Si3O7(OH)2:1.5H2O
H2(g)	-24	-27.14	-3.14	H2
halloy	-3.92	6.56	10.48	Al2Si2O9H4
hedenbergite	-1.03	18.81	19.84	CaFe(SiO3)2
hercynite	-3.88	23.8	27.68	FeAl2O4
heulandite-ca	3.69	5.15	1.47	CaAl2Si7O18:6H2O
hillebrandite	-8.59	23.97	32.55	Ca2SiO3(OH)2:0.167H2O
hydrogarnet	-27.87	52.93	80.8	Ca3Al2O6:6H2O

hydrotalcite	-9.69	65.65	75.34	Mg4Al2O17H20
jennite	-43.21	103.39	146.6	Ca9Si6O32H22
kaolinite	-2.54	-39.82	-37.29	Al2Si2O5(OH)4
katoite	-18.94	49.95	68.89	Ca3Al2SiO12H8
kyanite	-7.18	9.54	16.72	Al2SiO5
larnite	-15.45	23.97	39.42	Ca2SiO4
laumontite	-1.32	14.08	15.4	CaAl2Si4O12:4H2O
lawsonite	-3.15	20.03	23.19	CaAl2Si2O7(OH)2:H2O
margarite	-10.57	32.54	43.12	CaAl4Si2O10(OH)2
merwinite	-21.33	47.75	69.08	MgCa3(SiO4)2
Mg(H2Tartrate):5H2O	-1.81	-4.84	-3.03	Mg(H2Tartrate):5H2O
Mg(OH)2	-3.77	13.29	17.05	Mg(OH)2
minnesotaite	7.81	21.96	14.15	Fe3Si4O10(OH)2
monticellite	-6.35	23.78	30.13	CaMgSiO4
montmor-ca	1.8	5.15	3.35	Ca0.165Mg0.33Al1.67Si4O10(OH)2
montmor-mg	1.84	5.12	3.28	Mg0.495Al1.67Si4O10(OH)2
O2(g)	-36.14	-39.08	-2.95	O2
okenite	2.45	7.52	5.07	CaSi2O4(OH)2:H2O
phillipsite-ca	1.5	11.11	9.6	CaAl2Si5O14:5H2O
prehnite	-3.38	30.53	33.91	Ca2Al2Si3O10(OH)2
pseudo	-3.7	10.5	14.2	CaSiO3
pyrophyllite	-0.74	0.61	1.35	Al2Si4O10(OH)2
quartz	1.07	-2.98	-4.05	SiO2
rankinite	-18.11	34.47	52.57	Ca3Si2O7
ripidolit-14a	5.76	66.02	60.25	Mg3Fe2Al2Si3O10(OH)8
ripidolit-7a	2.37	66.02	63.64	Mg3Fe2Al2Si3O10(OH)8
saponite-ca	5.91	33.23	27.32	Ca0.165Mg3Al0.33Si3.67O10(OH)2
saponite-h	4.67	31	26.33	H0.33Mg3Al0.33Si3.67O10(OH)2
saponite-mg	5.9	33.2	27.3	Mg3.165Al0.33Si3.67O10(OH)2
scolecite	1.92	17.06	15.14	CaAl2Si3O10:3H2O
sepiolite	1.53	-38.75	-40.28	Mg2Si3O7.5OH:3H2O
sillimantite	-7.83	9.54	17.36	Al2SiO5
spinel	-13.28	25.8	39.07	Al2MgO4
stratlingite	-12.85	36.48	49.33	Ca2Al2Si1O15H16
talc	5.99	27.96	21.97	Mg3Si4O10(OH)2
tobermorite(11A)	-17.32	49.51	66.83	Ca5Si6H11O22.5
tobermorite(14A)	-14.82	49.51	64.32	Ca5Si6H21O27.5
tobermorite(9A)	-20.28	49.51	69.79	Ca5Si6H6O20
tremolite	6.9	69.57	62.67	Ca2Mg5Si8O22(OH)2
wairakite	-5.84	14.08	19.92	CaAl2Si4O12:2H2O
wollastonite	-3.29	10.5	13.79	CaSiO3
xonotlite	-4.47	62.98	67.45	Ca6Si6O17(OH)2
yugawaralite	-0.12	4.07	4.18	Ca0.5AlSi3O8:2H2O
zoisite	-8.05	36.79	44.84	Ca2Al3(SiO4)3OH

	temp		22					
	pH		6.4					
	pe		4					
	density		0.997					
	Si		78.17177					
	Al		889.5764	umol/kgw				
	Fe(2)		3.504252					
	Ca		2021.684					
	Mg		404.3367					
	Ti(4)		72.1836	umol/kgw				
	Citrate		0	mol/kgw				
	water		1	#	kg			
	Elements		Molality					
	Al		8.90E-04					
	Ca		5.04E-02					
	Fe(2)		6.28E-05					
	Mg		1.66E-02					
	Si		1.30E-03					
	Ti(4)		7.22E-05					
Distribution of species								
	Species		Molality	Activity	log Molality	log Activity	log Gamma	mole V cm³/mol
	H+		5.20E-07	3.98E-07	-6.284	-6.4	-0.116	0
	OH-		2.61E-08	2.00E-08	-7.584	-7.7	-0.116	0
	H2O		5.55E+01	9.99E-01	1.744	-0.001	0	18.06
Al		8.90E-04						
	Al(OH)2+		3.96E-04	3.03E-04	-3.403	-3.518	-0.116	0
	Al(OH)4-		3.19E-04	2.45E-04	-3.496	-3.612	-0.116	0
	Al(OH)3		9.35E-05	9.65E-05	-4.029	-4.016	0.013	0
	AlOH+2		7.04E-05	2.42E-05	-4.153	-4.616	-0.463	0
	Al+3		1.06E-05	9.62E-07	-4.975	-6.017	-1.042	0
Ca		5.04E-02						
	Ca+2		5.04E-02	1.74E-02	-1.297	-1.76	-0.463	0
	Ca(OH)+		1.11E-08	8.53E-09	-7.953	-8.069	-0.116	0
Fe(2)		6.28E-05						
	Fe+2		6.27E-05	2.16E-05	-4.203	-4.666	-0.463	0
	Fe(OH)+		1.78E-08	1.37E-08	-7.749	-7.864	-0.116	0
	Fe(OH)2		2.03E-13	2.09E-13	-12.693	-12.68	0.013	0
	Fe(OH)3-		2.65E-17	2.03E-17	-16.577	-16.693	-0.116	0
	Fe(OH)4-2		2.49E-25	8.56E-26	-24.605	-25.068	-0.463	0

H(0)		2.24E-24					
	H2	1.12E-24	1.16E-24	-23.95	-23.937	0.013	0
Mg		1.66E-02					
	Mg+2	1.66E-02	5.73E-03	-1.779	-2.242	-0.463	0
	MgOH+	2.34E-08	1.79E-08	-7.632	-7.747	-0.116	0
O(0)		0.00E+00					
	O2	0.00E+00	0.00E+00	-45.499	-45.485	0.013	0
Si		1.30E-03					
	H4SiO4	1.30E-03	1.34E-03	-2.886	-2.872	0.013	0
	H3SiO4-	6.13E-07	4.70E-07	-6.212	-6.328	-0.116	0
	H5Si2O7-	4.69E-08	3.59E-08	-7.329	-7.444	-0.116	0
	H4Si2O7-2	3.30E-12	1.14E-12	-11.481	-11.944	-0.463	0
	H2SiO4-2	1.31E-13	4.51E-14	-12.883	-13.346	-0.463	0
	H5Si3O10-3	1.34E-16	1.21E-17	-15.875	-16.916	-1.042	0
	H5Si4O12-3	1.80E-17	1.63E-18	-16.746	-17.788	-1.042	0
	H3Si3O9-3	1.06E-17	9.64E-19	-16.974	-18.016	-1.042	0
	H4Si4O12-4	4.62E-21	6.49E-23	-20.336	-22.188	-1.852	0
Ti(4)		7.22E-05					
	Ti(OH)4	7.22E-05	7.45E-05	-4.142	-4.128	0.013	0

Saturation indices

Phase	SI**	log	IAP	log
afwillite	-19.73	27.37	47.1	Ca3Si2O4(OH)6
akermanite	-18.88	26.89	45.77	Ca2MgSi2O7
Al(OH)3(amorph.)	2.18	13.18	11	Al(OH)3
amesite-14a	13.25	44.61	31.36	Mg2Al2SiO5(OH)4
amrph.silica	-0.15	-2.87	-2.72	SiO2
andalusite	6.5	23.49	17	Al2SiO5
anorthite	4.02	31.66	27.64	CaAl2(SiO4)2
anthophyllite	-18	50.93	68.93	Mg7Si8O22(OH)2
antigorite	22.55	204.56	182	Mg24Si17O42.5(OH)31
beidellit-ca	15.29	22	6.7	Ca0.165Al2.33Si3.67O10(OH)2
beidellit-mg	15.23	21.92	6.69	Mg0.165Al2.33Si3.67O10(OH)2
boehmite	3.37	13.18	9.81	AlO2H
Ca(OH)2	-12	11.04	23.04	Ca(OH)2
ca-al	-2.58	34.53	37.11	CaAl2SiO6
ca-p	9.74	29.94	20.2	CaAl2Si2.6O12.4H6.4
Ca2Al2O13H16	-10.19	48.44	58.63	Ca2Al2O13H16
Ca2Al2SiO15H16	-3.48	45.57	49.05	Ca2Al2SiO15H16
Ca2SiO4	-18.7	19.21	37.91	Ca2SiO4
Ca3Al2O12H12	-19.9	59.48	79.38	Ca3Al2O12H12
Ca3Al2Si0.5O12H10	-15.43	58.04	73.47	Ca3Al2Si0.5O12H10

Ca3SiO5	-44.55	30.25	74.79	Ca3SiO5
Ca4Al2O20H26	-31.78	70.52	102.3	Ca4Al2O20H26
CaO	-21.94	11.04	32.97	CaO
chabazite	12.33	25.92	13.58	CaAl2Si4O12:6H2O
chalcedony	0.68	-2.87	-3.55	SiO2
chamosite-7a	8.07	39.76	31.69	Fe2Al2SiO5(OH)4
chrysotile	-6.08	25.93	32.01	Mg3Si2O5(OH)4
chs(0.8)	-5.07	5.96	11.03	Ca0.8SiO5H4.4
clinochl-14a	1.97	70.54	68.57	Mg5Al2Si3O10(OH)8
clinochl-7a	-1.4	70.54	71.94	Mg5Al2Si3O10(OH)8
clinozoisite	8.3	53.01	44.71	Ca2Al3Si3O12(OH)
cordier.anh	4.42	59.49	55.07	Mg2Al4Si5O18
cordier.hydr	6.93	59.49	52.55	Mg2Al4Si5O18:H2O
corundum	4.53	26.36	21.84	Al2O3
crist.beta_amorph	0.15	-2.87	-3.02	SiO2
cristobalite	0.6	-2.87	-3.47	SiO2
csh(1.1)	-7.39	9.27	16.66	Ca1.1SiO7H7.8
csh(1.8)	-15.41	17	32.41	Ca1.8SiO9H10.4
daphnite-14a	7.11	58.42	51.31	Fe5Al2Si3O10(OH)8
daphnite-7a	3.71	58.42	54.71	Fe5Al2Si3O10(OH)8
diaspore	4.24	13.18	8.95	AlHO2
diopside	-5.42	15.85	21.28	CaMgSi2O6
enstatite	-3.97	7.69	11.65	MgSiO3
epistilbite	14.67	20.17	5.5	CaAl2Si6O16:5H2O
fayalite	-5.92	13.4	19.32	Fe2SiO4
Fe(OH)2	-5.88	8.13	14.01	Fe(OH)2
FeO	-5.53	8.13	13.66	FeO
ferrosilite	-2.27	5.26	7.53	FeSiO3
forsterite	-10.31	18.24	28.55	Mg2SiO4
foshagite	-23.87	35.54	59.41	Ca4Si3O9(OH)2:0.5H2O
gehlenite	-11.96	45.57	57.53	Ca2Al2SiO7
gibbsite	5.25	13.18	7.93	Al(OH)3
greenalite	-4.22	18.66	22.87	Fe3Si2O5(OH)4
grossular	-3.24	50.87	54.11	Ca3Al2(SiO4)3
gyrolite	-7.07	13.46	20.54	Ca2Si3O7(OH)2:1.5H2O
H2(g)	-20.8	-23.94	-3.14	H2
halloy	10.14	20.62	10.48	Al2Si2O9H4
hedenbergite	-6.41	13.43	19.84	CaFe(SiO3)2
hercynite	6.82	34.5	27.68	FeAl2O4
heulandite-ca	15.84	17.3	1.47	CaAl2Si7O18:6H2O
hillebrandite	-13.35	19.21	32.55	Ca2SiO3(OH)2:0.167H2O
hydrogarnet	-21.32	59.48	80.8	Ca3Al2O6:6H2O
hydrotalcite	-6.75	68.59	75.34	Mg4Al2O17H20
jennite	-64.48	82.12	146.6	Ca9Si6O32H22

kaolinite	11.52	-25.76	-37.29	Al2Si2O5(OH)4
katoite	-12.28	56.61	68.89	Ca3Al2SiO12H8
kyanite	6.77	23.49	16.72	Al2SiO5
larnite	-20.21	19.21	39.42	Ca2SiO4
laumontite	10.51	25.92	15.4	CaAl2Si4O12:4H2O
lawsonite	8.47	31.66	23.19	CaAl2Si2O7(OH)2:H2O
margarite	14.91	58.03	43.12	CaAl4Si2O10(OH)2
merwinite	-31.15	37.93	69.08	MgCa3(SiO4)2
Mg(OH)2	-6.5	10.56	17.05	Mg(OH)2
minnesotaite	-1.23	12.92	14.15	Fe3Si4O10(OH)2
monticellite	-11.41	18.73	30.13	CaMgSiO4
montmor-ca	12.48	15.83	3.35	Ca0.165Mg0.33Al1.67Si4O10(OH)2
montmor-mg	12.48	15.75	3.28	Mg0.495Al1.67Si4O10(OH)2
O2(g)	-42.54	-45.49	-2.95	O2
okenite	0.22	5.3	5.07	CaSi2O4(OH)2:H2O
phillipsite-ca	13.44	23.04	9.6	CaAl2Si5O14:5H2O
prehnite	5.92	39.83	33.91	Ca2Al2Si3O10(OH)2
pseudo	-6.03	8.17	14.2	CaSiO3
pyrophyllite	13.53	14.88	1.35	Al2Si4O10(OH)2
quartz	1.17	-2.87	-4.05	SiO2
rankinite	-25.2	27.37	52.57	Ca3Si2O7
ripidolit-14a	5.44	65.69	60.25	Mg3Fe2Al2Si3O10(OH)8

BFS_CA_6

temp		22		
ripidolit-7a	2.04	65.69	63.64	Mg3Fe2Al2Si3O10(OH)8
saponite-ca	-0.01	27.31	27.32	Ca0.165Mg3Al0.33Si3.67O10(OH)2
saponite-h	-0.85	25.48	26.33	H0.33Mg3Al0.33Si3.67O10(OH)2
saponite-mg	-0.07	27.23	27.3	Mg3.165Al0.33Si3.67O10(OH)2
scolecite	13.65	28.79	15.14	CaAl2Si3O10:3H2O
sepiolite	-3.62	-43.9	-40.28	Mg2Si3O7.5OH:3H2O
sillimantite	6.13	23.49	17.36	Al2SiO5
spinel	-2.15	36.92	39.07	Al2MgO4
stratlingite	-3.76	45.57	49.33	Ca2Al2Si1O15H16
talc	-1.78	20.19	21.97	Mg3Si4O10(OH)2
tobermorite(11A)	-28.87	37.96	66.83	Ca5Si6H11O22.5
tobermorite(14A)	-26.36	37.96	64.32	Ca5Si6H21O27.5
tobermorite(9A)	-31.82	37.97	69.79	Ca5Si6H6O20
tremolite	-10.78	51.89	62.67	Ca2Mg5Si8O22(OH)2
wairakite	6	25.92	19.92	CaAl2Si4O12:2H2O
wollastonite	-5.62	8.17	13.79	CaSiO3
xonotlite	-18.45	49.01	67.45	Ca6Si6O17(OH)2
yugawaralite	5.9	10.09	4.18	Ca0.5AlSi3O8:2H2O
zoisite	8.17	53.01	44.84	Ca2Al3(SiO4)3OH

	pH		6.4					
	pe		4					
	density		0.997					
	Si		90.99577					
	Al		1035.51	umol/kgw				
	Fe(2)		15.71745					
	Ca		2261.107					
	Mg		441.1916					
	Ti(4)		84.02525	umol/kgw				
	Citrate		0.048087	mol/kgw				
	water		1	#	kg			
	Elements		Molality					
	Al		1.04E-03					
	Ca		5.64E-02					
	Citrate		4.81E-02					
	Fe(2)		2.81E-04					
	Mg		1.82E-02					
	Si		1.51E-03					
	Ti(4)		8.40E-05					
Distribution of species								
	Species		Molality	Activity	log Molality	log Activity	log Gamma	mole V cm³/mol
	H+		5.22E-07	3.98E-07	-6.282	-6.4	-0.118	0
	OH-		2.62E-08	2.00E-08	-7.582	-7.7	-0.118	0
	H2O		5.55E+01	9.99E-01	1.744	-0.001	0	18.06
Al		1.04E-03						
	Al(Citrate)2-5		6.18E-04	7.07E-07	-3.209	-6.151	-2.942	0
	Al(Citrate)(HCitrate)-4		3.18E-04	4.16E-06	-3.498	-5.381	-1.883	0
	Al(OH)(Citrate)-2		5.15E-05	1.74E-05	-4.288	-4.759	-0.471	0
	Al(HCitrate)2-3		2.39E-05	2.09E-06	-4.622	-5.681	-1.059	0
	Al(Citrate)-		1.44E-05	1.10E-05	-4.841	-4.959	-0.118	0
	Al3(OH)4(HCitrate)3-4		2.78E-06	3.64E-08	-5.556	-7.439	-1.883	0
	Al2(OH)2(HCitrate)2-2		1.12E-06	3.78E-07	-5.952	-6.422	-0.471	0
	Al(HCitrate)		2.40E-08	2.48E-08	-7.62	-7.606	0.014	0
	Al3(OH)4(Citrate)3-7		6.12E-09	1.05E-14	-8.214	-13.979	-5.766	0
	Al(OH)2+		6.11E-11	4.66E-11	-10.214	-10.332	-0.118	0
	Al(OH)4-		4.93E-11	3.76E-11	-10.308	-10.425	-0.118	0
	Al(OH)3		1.43E-11	1.48E-11	-10.844	-10.829	0.014	0
	AlOH+2		1.10E-11	3.72E-12	-10.958	-11.429	-0.471	0
	AlH(HCitrate)+		5.11E-12	3.90E-12	-11.291	-11.409	-0.118	0
	Al+3		1.69E-12	1.48E-13	-11.771	-12.83	-1.059	0
Ca		5.64E-02						

	Ca+2		2.82E-02	9.53E-03	-1.55	-2.021	-0.471	0
	Ca(HCitrate)-		2.81E-02	2.15E-02	-1.551	-1.669	-0.118	0
	CaH(HCitrate)		1.40E-04	1.45E-04	-3.854	-3.84	0.014	0
	Ca(OH)+		6.13E-09	4.68E-09	-8.212	-8.33	-0.118	0
	CaH2(HCitrate)+		3.36E-13	2.57E-13	-12.473	-12.591	-0.118	0
Citrate		4.81E-02						
	Ca(HCitrate)-		2.81E-02	2.15E-02	-1.551	-1.669	-0.118	0
	HCitrate-3		8.58E-03	7.49E-04	-2.066	-3.125	-1.059	0
	Mg(HCitrate)-		8.56E-03	6.53E-03	-2.068	-2.185	-0.118	0
	Al(Citrate)2-5		6.18E-04	7.07E-07	-3.209	-6.151	-2.942	0
	H(HCitrate)-2		3.82E-04	1.29E-04	-3.418	-3.889	-0.471	0
	Al(Citrate)(HCitrate)-4		3.18E-04	4.16E-06	-3.498	-5.381	-1.883	0
	Fe(HCitrate)-		2.39E-04	1.82E-04	-3.622	-3.74	-0.118	0
	CaH(HCitrate)		1.40E-04	1.45E-04	-3.854	-3.84	0.014	0
	Al(OH)(Citrate)-2		5.15E-05	1.74E-05	-4.288	-4.759	-0.471	0
	MgH(HCitrate)		2.55E-05	2.64E-05	-4.593	-4.579	0.014	0
	Al(HCitrate)2-3		2.39E-05	2.09E-06	-4.622	-5.681	-1.059	0
	H4SiO4(HCitrate)-3		1.71E-05	1.49E-06	-4.767	-5.826	-1.059	0
	Al(Citrate)-		1.44E-05	1.10E-05	-4.841	-4.959	-0.118	0
	FeH(HCitrate)2-3		1.32E-05	1.15E-06	-4.88	-5.939	-1.059	0
	Al3(OH)4(HCitrate)3-4		2.78E-06	3.64E-08	-5.556	-7.439	-1.883	0
	Al2(OH)2(HCitrate)2-2		1.12E-06	3.78E-07	-5.952	-6.422	-0.471	0
	FeH(HCitrate)		9.60E-07	9.92E-07	-6.018	-6.004	0.014	0
	Fe2(Citrate)2-4		1.01E-07	1.32E-09	-6.998	-8.88	-1.883	0
	Al(HCitrate)		2.40E-08	2.48E-08	-7.62	-7.606	0.014	0
	Al3(OH)4(Citrate)3-7		6.12E-09	1.05E-14	-8.214	-13.979	-5.766	0
	AlH(HCitrate)+		5.11E-12	3.90E-12	-11.291	-11.409	-0.118	0
	H2(HCitrate)-		3.53E-12	2.69E-12	-11.452	-11.57	-0.118	0
	CaH2(HCitrate)+		3.36E-13	2.57E-13	-12.473	-12.591	-0.118	0
	MgH2(HCitrate)+		5.73E-14	4.37E-14	-13.242	-13.36	-0.118	0
	FeH2(HCitrate)+		4.30E-16	3.28E-16	-15.367	-15.485	-0.118	0
	H3(HCitrate)		3.70E-20	3.83E-20	-19.432	-19.417	0.014	0
Fe(2)		2.81E-04						
	Fe(HCitrate)-		2.39E-04	1.82E-04	-3.622	-3.74	-0.118	0
	Fe+2		2.86E-05	9.67E-06	-4.544	-5.015	-0.471	0
	FeH(HCitrate)2-3		1.32E-05	1.15E-06	-4.88	-5.939	-1.059	0
	FeH(HCitrate)		9.60E-07	9.92E-07	-6.018	-6.004	0.014	0
	Fe2(Citrate)2-4		1.01E-07	1.32E-09	-6.998	-8.88	-1.883	0
	Fe(OH)+		8.02E-09	6.11E-09	-8.096	-8.214	-0.118	0
	Fe(OH)2		9.05E-14	9.35E-14	-13.044	-13.029	0.014	0
	FeH2(HCitrate)+		4.30E-16	3.28E-16	-15.367	-15.485	-0.118	0
	Fe(OH)3-		1.19E-17	9.07E-18	-16.925	-17.042	-0.118	0
	Fe(OH)4-2		1.13E-25	3.83E-26	-24.947	-25.417	-0.471	0

H(0)		2.24E-24					
	H2	1.12E-24	1.16E-24	-23.951	-23.937	0.014	0
Mg		1.82E-02					
	Mg+2	9.57E-03	3.24E-03	-2.019	-2.49	-0.471	0
	Mg(HCitrate)-	8.56E-03	6.53E-03	-2.068	-2.185	-0.118	0
	MgH(HCitrate)	2.55E-05	2.64E-05	-4.593	-4.579	0.014	0
	MgOH+	1.33E-08	1.01E-08	-7.878	-7.995	-0.118	0
	MgH2(HCitrate)+	5.73E-14	4.37E-14	-13.242	-13.36	-0.118	0
O(0)		0.00E+00					
	O2	0.00E+00	0.00E+00	-45.5	-45.486	0.014	0
Si		1.51E-03					
	H4SiO4	1.50E-03	1.55E-03	-2.825	-2.811	0.014	0
	H4SiO4(HCitrate)-3	1.71E-05	1.49E-06	-4.767	-5.826	-1.059	0
	H3SiO4-	7.11E-07	5.42E-07	-6.148	-6.266	-0.118	0
	H5Si2O7-	6.27E-08	4.78E-08	-7.203	-7.32	-0.118	0
	H4Si2O7-2	4.47E-12	1.51E-12	-11.35	-11.82	-0.471	0
	H2SiO4-2	1.54E-13	5.20E-14	-12.813	-13.284	-0.471	0
	H5Si3O10-3	2.13E-16	1.86E-17	-15.671	-16.73	-1.059	0
	H5Si4O12-3	3.31E-17	2.89E-18	-16.481	-17.54	-1.059	0
	H3Si3O9-3	1.70E-17	1.48E-18	-16.771	-17.83	-1.059	0
	H4Si4O12-4	8.77E-21	1.15E-22	-20.057	-21.94	-1.883	0
Ti(4)		8.40E-05					
	Ti(OH)4	8.40E-05	8.69E-05	-4.076	-4.061	0.014	0

Saturation indices

Phase	SI**	log	IAP	log K(295 K,
afwillite	-20.39	26.71	47.1	Ca3Si2O4(OH)6
akermanite	-19.53	26.25	45.77	Ca2MgSi2O7
Al(OH)3(amorph.)	-4.63	6.37	11	Al(OH)3
amesite-14a	-0.81	30.55	31.36	Mg2Al2SiO5(OH)4
amrph.silica	-0.09	-2.81	-2.72	SiO2
andalusite	-7.07	9.93	17	Al2SiO5
anorthite	-9.74	17.9	27.64	CaAl2(SiO4)2
anthophyllite	-19.24	49.69	68.93	Mg7Si8O22(OH)2
antigorite	17.66	199.66	182	Mg24Si17O42.5(OH)31
beidellit-ca	-0.4	6.31	6.7	Ca0.165Al2.33Si3.67O10(OH)2
beidellit-mg	-0.46	6.23	6.69	Mg0.165Al2.33Si3.67O10(OH)2
boehmite	-3.44	6.37	9.81	AlO2H
Ca(OH)2	-12.26	10.78	23.04	Ca(OH)2
ca-al	-16.4	20.71	37.11	CaAl2SiO6
ca-p	-3.99	16.21	20.2	CaAl2Si2.6O12.4H6.4

Ca2Al2O13H16	-24.34	34.29	58.63	Ca2Al2O13H16
Ca2Al2SiO15H16	-17.57	31.48	49.05	Ca2Al2SiO15H16
Ca2SiO4	-19.16	18.75	37.91	Ca2SiO4
Ca3(HCitrate)2	4.72	-12.31	-17.03	Ca3(HCitrate)2
Ca3(HCitrate)2:4H2O	-0.48	-12.32	-11.84	Ca3(HCitrate)2:4H2O
Ca3Al2O12H12	-34.31	45.07	79.38	Ca3Al2O12H12
Ca3Al2Si0.5O12H10	-29.81	43.66	73.47	Ca3Al2Si0.5O12H10
Ca3SiO5	-45.27	29.53	74.79	Ca3SiO5
Ca4Al2O20H26	-46.46	55.84	102.3	Ca4Al2O20H26
CaH(HCitrate)	-0.16	-11.55	-11.39	CaH(HCitrate)
CaO	-22.2	10.78	32.97	CaO
chabazite	-1.31	12.28	13.58	CaAl2Si4O12:6H2O
chalcedony	0.75	-2.81	-3.55	SiO2
chamosite-7a	-6.19	25.5	31.69	Fe2Al2SiO5(OH)4
chrysotile	-6.7	25.31	32.01	Mg3Si2O5(OH)4
chs(0.8)	-5.22	5.81	11.03	Ca0.8SiO5H4.4
clinochl-14a	-12.71	55.86	68.57	Mg5Al2Si3O10(OH)8
clinochl-7a	-16.08	55.86	71.94	Mg5Al2Si3O10(OH)8
clinozoisite	-12.47	32.24	44.71	Ca2Al3Si3O12(OH)
cordier.anh	-23.02	32.05	55.07	Mg2Al4Si5O18
cordier.hydr	-20.51	32.05	52.55	Mg2Al4Si5O18:H2O
corundum	-9.1	12.74	21.84	Al2O3
crist.beta_amorph	0.21	-2.81	-3.02	SiO2
cristobalite	0.66	-2.81	-3.47	SiO2
csh(1.1)	-7.62	9.04	16.66	Ca1.1SiO7H7.8
csh(1.8)	-15.82	16.59	32.41	Ca1.8SiO9H10.4
daphnite-14a	-8.08	43.23	51.31	Fe5Al2Si3O10(OH)8
daphnite-7a	-11.48	43.23	54.71	Fe5Al2Si3O10(OH)8
diaspore	-2.58	6.37	8.95	AlHO2
diopside	-5.81	15.47	21.28	CaMgSi2O6
enstatite	-4.15	7.5	11.65	MgSiO3
epistilbite	1.15	6.66	5.5	CaAl2Si6O16:5H2O
fayalite	-6.56	12.76	19.32	Fe2SiO4
Fe(OH)2	-6.23	7.78	14.01	Fe(OH)2
FeO	-5.87	7.78	13.66	FeO
ferrosilite	-2.56	4.98	7.53	FeSiO3
forsterite	-10.74	17.81	28.55	Mg2SiO4
foshagite	-24.73	34.68	59.41	Ca4Si3O9(OH)2:0.5H2O
gehlenite	-26.04	31.49	57.53	Ca2Al2SiO7
gibbsite	-1.56	6.37	7.93	Al(OH)3
greenalite	-5.14	17.73	22.87	Fe3Si2O5(OH)4
grossular	-17.46	36.64	54.11	Ca3Al2(SiO4)3
gyrolite	-7.41	13.13	20.54	Ca2Si3O7(OH)2:1.5H2O
H2(g)	-20.8	-23.94	-3.14	H2

halloy	-3.36	7.12	10.48	Al2Si2O9H4
hedenbergite	-6.9	12.94	19.84	CaFe(SiO3)2
hercynite	-7.16	20.52	27.68	FeAl2O4
heulandite-ca	2.38	3.85	1.47	CaAl2Si7O18:6H2O
hillebrandite	-13.81	18.75	32.55	Ca2SiO3(OH)2:0.167H2O
hydrogarnet	-35.73	45.07	80.8	Ca3Al2O6:6H2O
hydrotalcite	-21.37	53.97	75.34	Mg4Al2O17H20
jennite	-66.46	80.14	146.6	Ca9Si6O32H22
kaolinite	-1.98	-39.27	-37.29	Al2Si2O5(OH)4
katoite	-26.63	42.26	68.89	Ca3Al2SiO12H8
kyanite	-6.79	9.93	16.72	Al2SiO5
larnite	-20.67	18.75	39.42	Ca2SiO4
laumontite	-3.13	12.28	15.4	CaAl2Si4O12:4H2O
lawsonite	-5.29	17.9	23.19	CaAl2Si2O7(OH)2:H2O
margarite	-12.48	30.63	43.12	CaAl4Si2O10(OH)2
merwinite	-32.06	37.03	69.08	MgCa3(SiO4)2
Mg(OH)2	-6.74	10.31	17.05	Mg(OH)2
minnesotaite	-2.03	12.12	14.15	Fe3Si4O10(OH)2
monticellite	-11.86	18.28	30.13	CaMgSiO4
montmor-ca	1.23	4.58	3.35	Ca0.165Mg0.33Al1.67Si4O10(OH)2
montmor-mg	1.22	4.5	3.28	Mg0.495Al1.67Si4O10(OH)2
O2(g)	-42.54	-45.49	-2.95	O2
okenite	0.09	5.16	5.07	CaSi2O4(OH)2:H2O
phillipsite-ca	-0.13	9.47	9.6	CaAl2Si5O14:5H2O
prehnite	-8.05	25.87	33.91	Ca2Al2Si3O10(OH)2
pseudo	-6.23	7.97	14.2	CaSiO3
pyrophyllite	0.15	1.5	1.35	Al2Si4O10(OH)2
quartz	1.24	-2.81	-4.05	SiO2
rankinite	-25.86	26.72	52.57	Ca3Si2O7
ripidolit-14a	-9.45	50.81	60.25	Mg3Fe2Al2Si3O10(OH)8
ripidolit-7a	-12.84	50.81	63.64	Mg3Fe2Al2Si3O10(OH)8
saponite-ca	-2.82	24.5	27.32	Ca0.165Mg3Al0.33Si3.67O10(OH)2
saponite-h	-3.61	22.72	26.33	H0.33Mg3Al0.33Si3.67O10(OH)2
saponite-mg	-2.88	24.42	27.3	Mg3.165Al0.33Si3.67O10(OH)2
scolecite	-0.05	15.09	15.14	CaAl2Si3O10:3H2O
sepiolite	-3.93	-44.21	-40.28	Mg2Si3O7.5OH:3H2O
sillimantite	-7.43	9.93	17.36	Al2SiO5
spinel	-16.03	23.05	39.07	Al2MgO4
stratlingite	-17.85	31.48	49.33	Ca2Al2Si1O15H16
talc	-2.27	19.69	21.97	Mg3Si4O10(OH)2
tobermorite(11A)	-29.8	37.03	66.83	Ca5Si6H11O22.5
tobermorite(14A)	-27.29	37.03	64.32	Ca5Si6H21O27.5
tobermorite(9A)	-32.75	37.03	69.79	Ca5Si6H6O20
tremolite	-12.04	50.63	62.67	Ca2Mg5Si8O22(OH)2

wairakite	-7.64	12.28	19.92	CaAl2Si4O12:2H2O
wollastonite	-5.82	7.97	13.79	CaSiO3
xonotlite	-19.64	47.81	67.45	Ca6Si6O17(OH)2
yugawaralite	-0.85	3.33	4.18	Ca0.5AlSi3O8:2H2O
zoisite	-12.6	32.24	44.84	Ca2Al3(SiO4)3OH

BFS_TA_6

temp	22		
pH	6.4		
pe	4		
density	0.997		
Si	160.0668		
Al	1821.522	umol/kgw	
Fe(2)	9.337229		
Ca	400.1669		
Mg	453.5225		
Ti(4)	147.8052	umol/kgw	
Tartrate	0.048087	mol/kgw	
water	1	#	kg

Elements	Molality
Al	1.82E-03
Ca	9.98E-03
Fe(2)	1.67E-04
Mg	1.87E-02
Si	2.66E-03
Tartrate	4.81E-02
Ti(4)	1.48E-04

Distribution of species

	Species	Molality	Activity	log Molality	log Activity	log Gamma	mole V cm³/mol
	H+	5.28E-07	3.98E-07	-6.278	-6.4	-0.122	0
	OH-	2.64E-08	2.00E-08	-7.578	-7.7	-0.122	0
	H2O	5.55E+01	9.99E-01	1.744	-0.001	0	18.06
Al	1.82E-03						
	Al(Tartrate)(HTartrate)2-7	1.74E-03	1.78E-09	-2.76	-8.749	-5.988	0
	Al(Tartrate)(HTartrate)-4	7.76E-05	8.60E-07	-4.11	-6.066	-1.955	0
	Al(Tartrate)2(HTartrate)-8	3.83E-06	5.77E-14	-5.417	-13.239	-7.822	0
	Al(Tartrate)-	2.14E-06	1.61E-06	-5.67	-5.792	-0.122	0
	Al(HTartrate)(H2Tartrate)-2	1.97E-06	6.38E-07	-5.707	-6.196	-0.489	0
	Al(HTartrate)	8.73E-08	9.07E-08	-7.059	-7.042	0.017	0
	Al(H2Tartrate)2-	1.77E-09	1.33E-09	-8.753	-8.876	-0.122	0
	Al2(Tartrate)2-2	4.95E-10	1.61E-10	-9.306	-9.795	-0.489	0
	Al(Tartrate)3-9	2.29E-10	2.89E-20	-9.64	-19.539	-9.899	0
	Al(OH)2+	7.98E-11	6.02E-11	-10.098	-10.22	-0.122	0

	Al(OH)4-		6.44E-11	4.86E-11	-10.191	-10.314	-0.122	0
	Al2(HTartrate)(Tartrate)-		5.72E-11	4.32E-11	-10.242	-10.365	-0.122	0
	Al(OH)3		1.84E-11	1.92E-11	-10.735	-10.718	0.017	0
	AlOH+2		1.48E-11	4.81E-12	-10.829	-11.318	-0.489	0
	Al(H2Tartrate)+		2.88E-12	2.18E-12	-11.54	-11.662	-0.122	0
	Al+3		2.40E-12	1.91E-13	-11.619	-12.719	-1.1	0
	Al2(HTartrate)2		2.68E-13	2.79E-13	-12.571	-12.555	0.017	0
	Al2(HTartrate)(H2Tartrate)+		5.99E-17	4.52E-17	-16.222	-16.345	-0.122	0
Ca		9.98E-03						
	Ca+2		7.73E-03	2.51E-03	-2.112	-2.601	-0.489	0
	Ca(H2Tartrate)		2.26E-03	2.35E-03	-2.647	-2.63	0.017	0
	CaH(H2Tartrate)+		2.54E-06	1.92E-06	-5.595	-5.717	-0.122	0
	Ca(OH)+		1.63E-09	1.23E-09	-8.787	-8.91	-0.122	0
Fe(2)		1.67E-04						
	Fe+2		9.97E-05	3.23E-05	-4.002	-4.49	-0.489	0
	Fe(H2Tartrate)		6.75E-05	7.02E-05	-4.171	-4.154	0.017	0
	Fe(OH)+		2.71E-08	2.05E-08	-7.567	-7.689	-0.122	0
	Fe(OH)2		3.01E-13	3.13E-13	-12.521	-12.504	0.017	0
	Fe(OH)3-		4.02E-17	3.04E-17	-16.395	-16.518	-0.122	0
	Fe(OH)4-2		3.95E-25	1.28E-25	-24.404	-24.892	-0.489	0
H(0)		2.22E-24						
	H2		1.11E-24	1.16E-24	-23.954	-23.937	0.017	0
Mg		1.87E-02						
	Mg+2		1.69E-02	5.47E-03	-1.773	-2.262	-0.489	0
	Mg(H2Tartrate)		1.81E-03	1.88E-03	-2.743	-2.726	0.017	0
	MgH(H2Tartrate)+		3.02E-06	2.28E-06	-5.521	-5.643	-0.122	0
	MgOH+		2.26E-08	1.71E-08	-7.646	-7.768	-0.122	0
O(0)		0.00E+00						
	O2		0.00E+00	0.00E+00	-45.502	-45.485	0.017	0
Si		2.66E-03						
	H4SiO4		2.66E-03	2.77E-03	-2.575	-2.558	0.017	0
	H3SiO4-		1.29E-06	9.70E-07	-5.891	-6.013	-0.122	0
	H5Si2O7-		2.03E-07	1.53E-07	-6.693	-6.815	-0.122	0
	H4Si2O7-2		1.49E-11	4.84E-12	-10.826	-11.315	-0.489	0
	H2SiO4-2		2.87E-13	9.31E-14	-12.542	-13.031	-0.489	0
	H5Si3O10-3		1.34E-15	1.07E-16	-14.872	-15.972	-1.1	0
	H5Si4O12-3		3.72E-16	2.96E-17	-15.429	-16.529	-1.1	0
	H3Si3O9-3		1.07E-16	8.48E-18	-15.972	-17.072	-1.1	0
	H4Si4O12-4		1.06E-19	1.18E-21	-18.974	-20.929	-1.955	0
Tartrate		4.81E-02						
	H2Tartrate-2		3.85E-02	1.25E-02	-1.414	-1.903	-0.489	0
	Ca(H2Tartrate)		2.26E-03	2.35E-03	-2.647	-2.63	0.017	0
	Mg(H2Tartrate)		1.81E-03	1.88E-03	-2.743	-2.726	0.017	0
	Al(Tartrate)(HTartrate)2-7		1.74E-03	1.78E-09	-2.76	-8.749	-5.988	0

	Al(Tartrate)(HTartrate)-4	7.76E-05	8.60E-07	-4.11	-6.066	-1.955	0
	Fe(H2Tartrate)	6.75E-05	7.02E-05	-4.171	-4.154	0.017	0
	H(H2Tartrate)-	6.19E-05	4.67E-05	-4.208	-4.33	-0.122	0
	Al(Tartrate)2(HTartrate)-8	3.83E-06	5.77E-14	-5.417	-13.239	-7.822	0
	MgH(H2Tartrate)+	3.02E-06	2.28E-06	-5.521	-5.643	-0.122	0
	CaH(H2Tartrate)+	2.54E-06	1.92E-06	-5.595	-5.717	-0.122	0
	Al(Tartrate)-	2.14E-06	1.61E-06	-5.67	-5.792	-0.122	0
	Al(HTartrate)(H2Tartrate)-2	1.97E-06	6.38E-07	-5.707	-6.196	-0.489	0
	HTartrate-3	1.47E-07	1.17E-08	-6.833	-7.933	-1.1	0
	Al(HTartrate)	8.73E-08	9.07E-08	-7.059	-7.042	0.017	0
	H2(H2Tartrate)	1.20E-08	1.25E-08	-7.921	-7.904	0.017	0
	Al(H2Tartrate)2-	1.77E-09	1.33E-09	-8.753	-8.876	-0.122	0
	Al2(Tartrate)2-2	4.95E-10	1.61E-10	-9.306	-9.795	-0.489	0
	Al(Tartrate)3-9	2.29E-10	2.89E-20	-9.64	-19.539	-9.899	0
	Al2(HTartrate)(Tartrate)-	5.72E-11	4.32E-11	-10.242	-10.365	-0.122	0
	Al(H2Tartrate)+	2.88E-12	2.18E-12	-11.54	-11.662	-0.122	0
	Tartrate-4	2.83E-13	3.14E-15	-12.548	-14.503	-1.955	0
	Al2(HTartrate)2	2.68E-13	2.79E-13	-12.571	-12.555	0.017	0
	Al2(HTartrate)(H2Tartrate)+	5.99E-17	4.52E-17	-16.222	-16.345	-0.122	0
Ti(4)	1.48E-04						
	Ti(OH)4	1.48E-04	1.54E-04	-3.83	-3.813	0.017	0

Saturation indices

Phase	SI**	log	IAP	log	K(295	K,
afwillite	-21.62	25.48	47.1	Ca3Si2O4(OH)6		
akermanite	-19.95	25.82	45.77	Ca2MgSi2O7		
Al(OH)3(amorph.)	-4.52	6.48	11	Al(OH)3		
amesite-14a	0.12	31.48	31.36	Mg2Al2SiO5(OH)4		
amrph.silica	0.16	-2.56	-2.72	SiO2		
andalusite	-6.59	10.4	17	Al2SiO5		
anorthite	-9.6	18.05	27.64	CaAl2(SiO4)2		
anthophyllite	-15.63	53.31	68.93	Mg7Si8O22(OH)2		
antigorite	27.42	209.42	182	Mg24Si17O42.5(OH)31		
beidellit-ca	0.69	7.4	6.7	Ca0.165Al2.33Si3.67O10(OH)2		
beidellit-mg	0.76	7.45	6.69	Mg0.165Al2.33Si3.67O10(OH)2		
boehmite	-3.33	6.48	9.81	AlO2H		
Ca(H2Tartrate):4H2O	1.47	-4.51	-5.98	Ca(H2Tartrate):4H2O		
Ca(OH)2	-12.84	10.2	23.04	Ca(OH)2		
ca-al	-16.51	20.6	37.11	CaAl2SiO6		
ca-p	-3.69	16.51	20.2	CaAl2Si2.6O12.4H6.4		
Ca2Al2O13H16	-25.28	33.35	58.63	Ca2Al2O13H16		
Ca2Al2SiO15H16	-18.25	30.8	49.05	Ca2Al2SiO15H16		

Ca2SiO4	-20.07	17.84	37.91	Ca2SiO4
Ca3Al2O12H12	-35.83	43.55	79.38	Ca3Al2O12H12
Ca3Al2Si0.5O12H10	-31.19	42.28	73.47	Ca3Al2Si0.5O12H10
Ca3SiO5	-46.75	28.04	74.79	Ca3SiO5
Ca4Al2O20H26	-48.55	53.75	102.3	Ca4Al2O20H26
CaO	-22.78	10.2	32.97	CaO
chabazite	-0.65	12.93	13.58	CaAl2Si4O12:6H2O
chalcedony	1	-2.56	-3.55	SiO2
chamosite-7a	-4.67	27.02	31.69	Fe2Al2SiO5(OH)4
chrysotile	-5.51	26.5	32.01	Mg3Si2O5(OH)4
chs(0.8)	-5.43	5.6	11.03	Ca0.8SiO5H4.4
clinochl-14a	-10.6	57.97	68.57	Mg5Al2Si3O10(OH)8
clinochl-7a	-13.97	57.97	71.94	Mg5Al2Si3O10(OH)8
clinozoisite	-12.54	32.17	44.71	Ca2Al3Si3O12(OH)
cordier.anh	-20.85	34.21	55.07	Mg2Al4Si5O18
cordier.hydr	-18.34	34.21	52.55	Mg2Al4Si5O18:H2O
corundum	-8.88	12.96	21.84	Al2O3
crist.beta_amorph	0.46	-2.56	-3.02	SiO2
cristobalite	0.91	-2.56	-3.47	SiO2
csh(1.1)	-8	8.66	16.66	Ca1.1SiO7H7.8
csh(1.8)	-16.61	15.8	32.41	Ca1.8SiO9H10.4
daphnite-14a	-4.48	46.83	51.31	Fe5Al2Si3O10(OH)8
daphnite-7a	-7.87	46.83	54.71	Fe5Al2Si3O10(OH)8
diaspore	-2.47	6.48	8.95	AlHO2
diopside	-5.66	15.62	21.28	CaMgSi2O6
enstatite	-3.67	7.98	11.65	MgSiO3
epistilbite	2.31	7.82	5.5	CaAl2Si6O16:5H2O
fayalite	-5.25	14.06	19.32	Fe2SiO4
Fe(H2Tartrate):3H2O	1.83	-6.4	-8.23	Fe(H2Tartrate):3H2O
Fe(OH)2	-5.7	8.31	14.01	Fe(OH)2
FeO	-5.35	8.31	13.66	FeO
ferrosilite	-1.78	5.75	7.53	FeSiO3
forsterite	-10.04	18.52	28.55	Mg2SiO4
foshagite	-26.29	33.12	59.41	Ca4Si3O9(OH)2:0.5H2O
gehlenite	-26.73	30.8	57.53	Ca2Al2SiO7
gibbsite	-1.45	6.48	7.93	Al(OH)3
greenalite	-3.06	19.81	22.87	Fe3Si2O5(OH)4
grossular	-18.22	35.89	54.11	Ca3Al2(SiO4)3
gyrolite	-7.81	12.73	20.54	Ca2Si3O7(OH)2:1.5H2O
H2(g)	-20.8	-23.94	-3.14	H2
halloy	-2.63	7.85	10.48	Al2Si2O9H4
hedenbergite	-6.45	13.39	19.84	CaFe(SiO3)2
hercynite	-6.41	21.27	27.68	FeAl2O4
heulandite-ca	3.79	5.26	1.47	CaAl2Si7O18:6H2O

hillebrandite	-14.71	17.84	32.55	Ca2SiO3(OH)2:0.167H2O
hydrogarnet	-37.25	43.55	80.8	Ca3Al2O6:6H2O
hydrotalcite	-20.24	55.1	75.34	Mg4Al2O17H20
jennite	-70.16	76.44	146.6	Ca9Si6O32H22
kaolinite	-1.25	-38.54	-37.29	Al2Si2O5(OH)4
katoite	-27.89	41	68.89	Ca3Al2SiO12H8
kyanite	-6.31	10.4	16.72	Al2SiO5
larnite	-21.58	17.84	39.42	Ca2SiO4
laumontite	-2.47	12.93	15.4	CaAl2Si4O12:4H2O
lawsonite	-5.14	18.04	23.19	CaAl2Si2O7(OH)2:H2O
margarite	-12.11	31.01	43.12	CaAl4Si2O10(OH)2
merwinite	-33.06	36.02	69.08	MgCa3(SiO4)2
Mg(H2Tartrate):5H2O	-1.14	-4.17	-3.03	Mg(H2Tartrate):5H2O
Mg(OH)2	-6.52	10.54	17.05	Mg(OH)2
minnesotaite	0.55	14.7	14.15	Fe3Si4O10(OH)2
monticellite	-11.95	18.18	30.13	CaMgSiO4
montmor-ca	2.4	5.75	3.35	Ca0.165Mg0.33Al1.67Si4O10(OH)2
montmor-mg	2.53	5.81	3.28	Mg0.495Al1.67Si4O10(OH)2
O2(g)	-42.54	-45.49	-2.95	O2
okenite	0.01	5.08	5.07	CaSi2O4(OH)2:H2O
phillipsite-ca	0.77	10.37	9.6	CaAl2Si5O14:5H2O
prehnite	-8.22	25.69	33.91	Ca2Al2Si3O10(OH)2
pseudo	-6.55	7.64	14.2	CaSiO3
pyrophyllite	1.38	2.73	1.35	Al2Si4O10(OH)2
quartz	1.49	-2.56	-4.05	SiO2
rankinite	-27.09	25.48	52.57	Ca3Si2O7
ripidolit-14a	-6.73	53.52	60.25	Mg3Fe2Al2Si3O10(OH)8
ripidolit-7a	-10.13	53.52	63.64	Mg3Fe2Al2Si3O10(OH)8
saponite-ca	-1.27	26.05	27.32	Ca0.165Mg3Al0.33Si3.67O10(OH)2
saponite-h	-1.97	24.37	26.33	H0.33Mg3Al0.33Si3.67O10(OH)2
saponite-mg	-1.2	26.1	27.3	Mg3.165Al0.33Si3.67O10(OH)2
scolecite	0.35	15.49	15.14	CaAl2Si3O10:3H2O
sepiolite	-2.72	-43	-40.28	Mg2Si3O7.5OH:3H2O
sillimantite	-6.96	10.4	17.36	Al2SiO5
spinel	-15.58	23.5	39.07	Al2MgO4
stratlingite	-18.53	30.8	49.33	Ca2Al2Si1O15H16
talc	-0.58	21.38	21.97	Mg3Si4O10(OH)2
tobermorite(11A)	-31.18	35.65	66.83	Ca5Si6H11O22.5
tobermorite(14A)	-28.67	35.65	64.32	Ca5Si6H21O27.5
tobermorite(9A)	-34.14	35.65	69.79	Ca5Si6H6O20
tremolite	-10.04	52.63	62.67	Ca2Mg5Si8O22(OH)2
wairakite	-6.99	12.93	19.92	CaAl2Si4O12:2H2O
wollastonite	-6.15	7.64	13.79	CaSiO3
xonotlite	-21.6	45.85	67.45	Ca6Si6O17(OH)2

yugawaralite	-0.27	3.91	4.18	Ca0.5AlSi3O8:2H2O
zoisite	-12.67	32.17	44.84	Ca2Al3(SiO4)3OH

References

(1) Trens, P.; Denoyel, R.; Guilloteau, E. Evolution of Surface Composition, Porosity, and Surface Area of Glass Fibers in a Moist Atmosphere. *Langmuir* **1996**, *12* (5), 1245–1250. https://doi.org/10.1021/la950531e.

(2) Majérus, O.; Lehuédé, P.; Biron, I.; Alloteau, F.; Narayanasamy, S.; Caurant, D. Glass Alteration in Atmospheric Conditions: Crossing Perspectives from Cultural Heritage, Glass Industry, and Nuclear Waste Management. *npj Materials Degradation* **2020**, *4* (1), 1–16. https://doi.org/10.1038/s41529-020-00130-9.

(3) Blotevogel, S.; Ehrenberg, A.; Steger, L.; Doussang, L.; Kaknics, J.; Patapy, C.; Cyr, M. Ability of the R3 Test to Evaluate Differences in Early Age Reactivity of 16 Industrial Ground Granulated Blast Furnace Slags (GGBS). *Cement and Concrete Research* **2020**, *130*. https://doi.org/10.1016/j.cemconres.2020.105998.

(4) Alves, J. O.; Espinosa, D. C. R.; Tenório, J. A. S.; Alves, J. O.; Espinosa, D. C. R.; Tenório, J. A. S. Recovery of Steelmaking Slag and Granite Waste in the Production of Rock Wool. *Materials Research* **2015**, *18* (1), 204–211. https://doi.org/10.1590/1516-1439.324414.

(5) Moesgaard, M.; Pedersen, H. D.; Yue, Y. Z.; Nielsen, E. R. Crystallization in Stone Wool Fibres. *Journal of Non-Crystalline Solids* **2007**, *353* (11), 1101–1108. https://doi.org/10.1016/j.jnoncrysol.2006.12.026.

(6) Descostes, M.; Mercier, F.; Thromat, N.; Beaucaire, C.; Gautier-Soyer, M. Use of XPS in the Determination of Chemical Environment and Oxidation State of Iron and Sulfur Samples: Constitution of a Data Basis in Binding Energies for Fe and S Reference Compounds and Applications to the Evidence of Surface Species of an Oxidized Pyrite in a Carbonate Medium. *Applied Surface Science* **2000**, *165* (4), 288–302. https://doi.org/10.1016/S0169-4332(00)00443-8.

(7) Seyama, H.; Soma, M. Fe 2p Spectra of Silicate Minerals. *Journal of Electron Spectroscopy and Related Phenomena* **1987**, *42* (1), 97–101. https://doi.org/10.1016/0368-2048(87)85010-7.

(8) Kirkegaard, L. F.; Korsgaard, M.; Yue, Y.; Mørup, S. Redox Behaviour of Iron Bearing Glass Fibres during Heat Treatment under Atmospheric Conditions. *Physics and Chemistry of Glasses* **2005**, *78* (1), 1–6.

(9) Bernal, S. A.; Rose, V.; Provis, J. L. The Fate of Iron in Blast Furnace Slag Particles during Alkali-Activation. *Materials Chemistry and Physics* **2014**, *146* (1–2), 1–5. https://doi.org/10.1016/j.matchemphys.2014.03.017.

(10) Mancini, A.; Lothenbach, B.; Geng, G.; Grolimund, D.; Sanchez, D. F.; Fakra, S. C.; Dähn, R.; Wehrli, B.; Wieland, E. Iron Speciation in Blast Furnace Slag Cements. *Cement and Concrete Research* **2021**, *140*, 106287. https://doi.org/10.1016/j.cemconres.2020.106287.

(11) Bingham, P. A.; Connelly, A. J.; Hand, R. J.; Hyatt, N. C.; Northrup, P. A.; Alonso Mori, R.; Glatzel, P.; Kavčič, M.; Žitnik, M.; Bučar, K.; Edge, R. A Multi-Spectroscopic Investigation of Sulphur Speciation in Silicate Glasses and Slags. *Glass Technology: European Journal of Glass Science and Technology Part A* **2010**, *51* (2), 63–80.

(12) Zhu, Y.; Elzinga, E. J. Formation of Layered Fe(II)-Hydroxides during Fe(II) Sorption onto Clay and Metal-Oxide Substrates. *Environ Sci Technol* **2014**, *48* (9), 4937–4945. https://doi.org/10.1021/es500579p.
